# The causal relationship between immune cells and Sjögren’s syndrome: a univariate, multivariate, bidirectional Mendelian randomized study

**DOI:** 10.3389/fmed.2024.1408562

**Published:** 2024-07-02

**Authors:** Wen Zeng, Mu Huang, Yuanyuan Zeng, Jie Pan, Fang Qin, Xiaoling Liao, Leting Zheng, Ling Lei

**Affiliations:** Department of Rheumatology and Immunology, The First Affiliated Hospital of Guangxi Medical University, Nanning, China

**Keywords:** Mendelian randomization, immune cells, Sjögren’s syndrome, causal relationship, B cell

## Abstract

**Introduction:**

Immune cells are involved in the onset and progression of Sjögren’s syndrome (SS). This study explored the causal relationship between immune signature cells and SS, which has not been fully elucidated.

**Methods:**

We conducted univariate, multivariate, and bidirectional Mendelian randomization to investigate the causal relationship between 731 immunological feature characteristic cells and SS pairs and explore the interaction of immune cells in SS.

**Results:**

After false discovery rate correction, six immune cells were significantly associated with SS risk. Among them, four contributed to SS (CD24 on memory B cell, CD27 on IgD + CD24 + B cell, CD28 on CD39+ secreting CD4 Treg cell, and CD80 on CD62L + mDC); two appeared to reduce SS risk (CD3 on CD39 + CD8 + T cell and CD38 on IgD + CD38 + B cell). Pleiotropy and heterogeneity were not observed. Three immune cells exerted independent effects for SS (CD27 on IgD + CD24 + B cell, CD80 on CD62L + mDC, and CD38 on IgD + CD38 + B cell); two were risk factors (CD27 on IgD + CD24 + B cell and CD80 on CD62L + mDC); and one was a protective factor (CD38 on IgD + CD38 + B cell). Twenty-three immune cells showed a reverse causal relationship with SS.

**Conclusion:**

These findings demonstrate the influence of immune cells on SS risk and the effects of SS on immune cells, providing new clues for further research on the mechanisms underlying SS.

## Introduction

Sjögren’s syndrome (SS) is a heterogeneous, etiological, systemic autoimmune disease characterized by chronic inflammation and dysfunction of the exocrine glands. SS can affect different organs and tissues and is usually accompanied by sicca symptoms, such as fatigue, chronic pain, and multiple organ-related symptoms (1, 2). SS is the second most common autoimmune rheumatic disease, affecting between 0.4 and 3.1 million individuals (3). SS causes a health burden for patients and substantial social disease costs, necessitating early diagnosis and active intervention to improve the prognosis of SS.

The pathogenesis of SS is very complex and has not been fully elucidated. Increasingly more evidence has shown that immune system dysfunction plays a crucial role in the etiology of SS, and the interaction between inflammation and genetic and environmental variables influences the occurrence, development, and resulting tissue damage of SS (4). Studies have shown that disturbances of the innate immune barrier involving the interferon (IFN) pathway in the early stages of SS disease are involved in the etiology of SS (5, 6). Vogelsang et al. reported a significant reduction in myeloid dendritic cells (mDCs) and plasmacytoid dendritic cells (pDCs) in the peripheral blood as well as the presence of pDC in salivary glands of SS patients compared to healthy controls (7). Zhao et al. reported that plasma cell-type DCs were enriched in the minor salivary gland of SS, inducing CXCR5(+)CD19(+) B cells to accumulate by secreting type I IFN (8). In addition, the adaptive immune system plays an integral role in the development of SS, and polyclonal overactivation of B cells and proliferation of Th1 and Th17 cells contribute to the progression of the disease (9). Epithelial function is also involved in the complex etiology of SS (10). However, the results of studies on the causal relationship between SS and immune cells have been inconsistent to date, which may be due to insufficient sample sizes, confounding factors, and biases.

Recent advances in large-scale genome-wide association studies (GWASs) and Mendelian randomization (MR) methods have made it possible to assess causal relationships between immune cells and disease outcomes. Compared to other statistical methods, MR can reduce the bias caused by confounding and reverse causation (11, 12). In this study, our research applied univariate, multivariate, and bidirectional MR analyses to investigate the impact of multiple variables and the causal relationship between immune cells and SS risk to clarify the association between immune cell characteristics and SS.

## Materials and methods

### Study design

**Figure 1:**
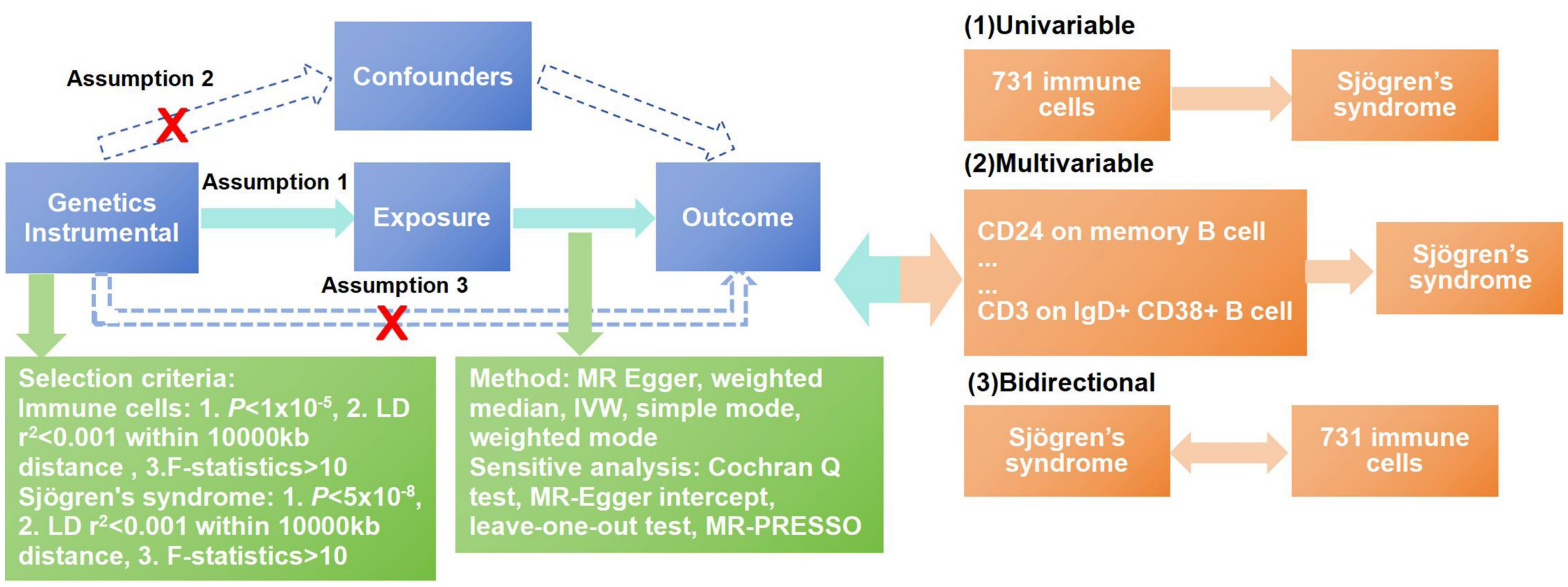
Workflow of the MR analysis. In this study, univariate Mendelian randomization (MR) was conducted to investigate the causal associations between immune cells and SS, and five methods were adopted to ensure the reliability of the result, including MR-Egger, weighted median, inverse-variance weighting (IVW), weighted mode and simple mode. The MR-PRESSO test, Cochran’s Q-test, MR-Egger intercept analysis, and leave-one-out test were used to observe the pleiotropy and heterogeneity of the results. To overcome the interference between immune cells, we used multivariable MR analysis to further correct the independent effects of these associated immune cells for SS. At last, a bidirectional MR analysis was conducted to explore the reverse causal relationships between immune cells and SS.

Our study investigated the causal associations between 731 immune cells and SS based on MR analysis. In the process of our research, MR analysis was used to observe three core assumptions to ensure unbiased causal effects: (1) Genetic variants are closely related to the exposure, (2) genetic variants are not associated with potential confounders, and (3) genetic variants affect outcomes only by the exposure pathway (13). Data summaries about immune cells and SS were acquired from publicly accessible GWAS. Therefore, there was no need to obtain ethical approval. The workflow of our research is shown in Figure 1.

### GWAS data source for 731 immune cells

The GWAS data for the 731 immune cells, which were acquired from a public catalog (GCST90001391-GCST90002121), were based on a study of the genetic characteristics of immune cells. The study aimed to examine the effects of 22 million genetic variations on 731 immune cell cells among 3,757 Sardinians and further verify the relationships among autoimmune illnesses and immunological characteristics. According to this study, we could understand the types of GWAS data, which include 389 median fluorescence intensity measurements representing surface antigen levels, 192 relative cell counts, 118 absolute cell counts, and 32 morphological characteristics (14).

### GWAS data source for Sjögren’s syndrome

We chose the GWAS data for SS from the FinnGen dataset^1^ for MR analysis (dataset finn-b-M13_SJOGREN), including 16,380,454 single-nucleotide polymorphisms (SNPs), and the GWAS was performed on 214,435 Europeans (nCase = 1,290, nControl = 213,145).

### Instrumental variable selection

Recent research indicated that when selecting important SNPs for various immune trait cells, we should choose a loose cutoff value of *p* < 1 × 10^−5^ (15). According to the 1,000 Genomes Projects reference panel, the linkage disequilibrium *r*^2^ threshold was set to be <0.001 within a 10,000-kb distance to remove the genetic linkage imbalance effect. In the reverse MR analysis, we chose a stricter standard value of *p* < 5 × 10^−8^ and *r*^2^ < 0.001 within a 10,000-kb distance. Finally, SNPs with low F statistics (<10) were removed to avoid weak instrumental bias, and the rest were used as instrumental variables for MR analysis.

### Statistical analysis

Our study mainly adopted the inverse-variance weighting (IVW) method to observe the causal relationship between the 731 immune cell types and SS, while four additional supplemental methods were used, including MR-Egger, weighted median, weighted mode, and simple mode. We comprehensively analyzed the results of five methods to ensure the reliability of the results. At the same time, multiple methods were used to observe for possible pleiotropy and heterogeneity among the results. Cochran’s Q-test was utilized to assess for heterogeneity, the MR-Egger intercept was utilized to address and account for pleiotropy, the leave-one-out analysis was utilized to assess robustness, and the MR Pleiotropy RESidual Sum and Outlier (MR-PRESSO) test was used to assess for horizontal pleiotropy and detect value bias.

In our study, R statistical software (version 4.3.2) was used to complete all statistical analyses using “Two Sample MR” (version 0.5.8) packages and the MR-PRESSO package (version 1.0).

## Results

### The causal effect of immune cells on Sjögren’s syndrome

Through univariable MR analysis, 22 immune cells were found to be causally related to SS risk. After correcting for the false discovery rate (FDR), we identified six immune cells that have a causal relationship with SS, and they were, respectively, distributed in three B cells, two regulatory T cells (Tregs), and one classical DC (cDC). The result of applying the IVW method indicated that CD24 on memory B cell (odds ratio [OR] = 1.094, 95% confidence interval (CI) 1.026–1.165, *p* = 0.06, positive FDR [*P*_FDR_] = 0.031) increased the risk of SS, and the results of the other four methods were similar: MR-Egger (OR = 1.138, 95%CI 1.048–1.236, *p* = 0.005), weighted median (OR = 1.105, 95%CI 1.000–1.220, *p* = 0.049), simple mode (OR = 1.051, 95%CI 0.881–1.254, *p* = 0.584), and weighted mode (OR = 1.122, 95%CI 1.032–1.219, *p* = 0.011). CD27 on IgD+ CD24+ B cell (OR = 1.115, 95%CI 1.044–1.191, *p* = 0.001, *P*_FDR_ = 0.027) also increased the risk of SS, and the results of the other four methods were similar: MR-Egger (OR = 1.153, 95%CI 1.053–1.262, *p* = 0.004), weighted median (OR = 1.151, 95%CI 1.041–1.271, *p* = 0.006), simple mode (OR = 1.005,95%CI 0.839–1.204, *p* = 0.958), and weighted mode (OR = 1.167,95%CI 1.064–1.280, *p* = 0.003). CD28 on CD39+ secreting CD4 regulatory T cell (OR = 1.096, 95%CI 1.006–1.195, *p* = 0.036, *P*_FDR_ = 0.050) was a risk factor for SS, and the results of the other four methods were similar: MR-Egger (OR = 1.070, 95%CI 0.946–1.210, *p* = 0.295), weighted median (OR = 1.049, 95%CI 0.929–1.183, *p* = 0.440), simple mode (OR = 1.156, 95%CI 0.953–1.403, *p* = 0.155), and weighted mode (OR = 1.046, 95%CI 0.933–1.172, *p* = 0.451). CD80 on CD62L+ mDC (OR = 1.102, 95%CI 1.033–1.176, *p* = 0.003, *P*_FDR_ = 0.023) was also a risk factor, and the results of the other four methods were similar: MR-Egger (OR = 1.090, 95%CI 0.998–1.189, *p* = 0.067), weighted median (OR = 1.078, 95%CI 0.987–1.178, *p* = 0.097), simple mode (OR = 1.066, 95%CI 0.916–1.241, *p* = 0.414), and weighted mode (OR = 1.086, 95%CI 1.002–1.177, *p* = 0.055). In addition, CD3 on CD39+ CD8+ T cell (OR = 0.884, 95%CI 0.782–1.000, *p* = 0.049, *P*_FDR_ = 0.049) reduced the risk of SS, and the results of the other four methods were similar: MR-Egger (OR = 0.874, 95%CI 0.645–1.185, *p* = 0.402), weighted median (OR = 0.894, 95%CI 0.750–1.065, *p* = 0.208), simple mode (OR = 0.953, 95%CI 0.752–1.208, *p* = 0.699), and weighted mode (OR = 0.886, 95%CI 0.736–1.066, *p* = 0.220). CD38 on IgD+ CD38+ B cell (OR = 0.861, 95%CI 0.781–0.949, *p* = 0.003, *P*_FDR_ = 0.028) reduced the risk of SS, and the results of the other four methods were similar: MR-Egger (OR = 0.833, 95%CI 0.707–0.981, *p* = 0.045), weighted median (OR = 0.867, 95%CI 0.754–0.998, *p* = 0.046), simple mode (OR = 0.789, 95%CI 0.633–0.983, *p* = 0.051), and weighted mode (OR = 0.859, 95%CI 0.748–0.987, *p* = 0.048) (Table 1). Furthermore, we used Cochran’s Q-test to assess heterogeneity and the MR-Egger intercept to account for pleiotropy; and the *p*-values of all results were greater than 0.05 (Table 2). The leave-one-out analysis showed that these data were stable. Finally, we did not observe any horizontal pleiotropy nor biased values through the MR-PRESSO test (Table 2) (Supplementary material S1).

**Table 1:** Causal effects of immune cells on Sjögren’s syndrome.

Exposure	Outcome	Method	OR (95% CI)	*p-*value	Adjust *p*
CD3 on CD39+ CD8br	Sjögren’s syndrome	MR-Egger	0.874 (0.645–1.185)	0.40214	
		Weighted median	0.894 (0.750–1.065)	0.20806	
		IVW	0.884 (0.782–1.000)	0.04949	0.04949
		Simple mode	0.953 (0.752–1.208)	0.69922	
		Weighted mode	0.886(0.736–1.066)	0.22004	
CD38 on IgD+ CD38br		MR-Egger	0.833 (0.707–0.981)	0.04509	
		Weighted median	0.867 (0.754–0.998)	0.04612	
		IVW	0.861 (0.781–0.949)	0.00251	0.02759
		Simple mode	0.789 (0.633–0.983)	0.05086	
		Weighted mode	0.859(0.748–0.987)	0.04764	
CD80 on CD62L+ myeloid DC		MR-Egger	1.090 (0.998–1.189)	0.06653	
		Weighted median	1.078(0.987–1.178)	0.09682	
		IVW	1.102 (1.033–1.176)	0.00315	0.02311
		Simple mode	1.066(0.916–1.241)	0.41419	
		Weighted mode	1.086 (1.002–1.177)	0.05544	
CD27 on IgD+ CD24+		MR-Egger	1.153(1.053–1.262)	0.00445	
		Weighted median	1.151 (1.041–1.271)	0.00586	
		IVW	1.115(1.044–1.191)	0.00124	0.02718
		Simple mode	1.005 (0.839–1.204)	0.95787	
		Weighted mode	1.167(1.064–1.280)	0.00268	
CD28 on CD39+ secreting Treg		MR-Egger	1.070(0.946–1.210)	0.29487	
		Weighted median	1.049 (0.929–1.183)	0.43962	
		IVW	1.096 (1.006–1.195)	0.03611	0.04965
		Simple mode	1.156(0.953–1.403)	0.15501	
		Weighted mode	1.046 (0.933–1.172)	0.45098	
CD24 on memory B cell		MR-Egger	1.138 (1.048–1.236)	0.00462	
		Weighted median	1.105 (1.000–1.220)	0.04889	
		IVW	1.094 (1.026–1.165)	0.00562	0.03090
		Simple mode	1.051 (0.881–1.254)	0.58393	
		Weighted mode	1.122 (1.032–1.219)	0.01125	

MR, Mendelian randomization; IVW, inverse-variance weighting; CI, confidence interval; DC, dendritic cell; br, bright; Treg, regulatory T.

**Table 2:** Sensitivity analysis results of causal effects of immune cells on Sjögren’s syndrome.

Immune cell	Egger intercept	Cochran’s Q-test		MR-PRESSO
		MR-Egger	IVW	
CD24 on memory B cell	0.152	0.965	0.940	0.943
CD27 on IgD+ CD24+ B cell	0.300	0.757	0.748	0.773
CD28 on CD39+ secreting CD4 regulatory T cell	0.580	0.225	0.255	0.228
CD3 on CD39+ CD8+ T cell	0.937	0.408	0.485	0.529
CD38 on IgD+ CD38+ B cell	0.627	0.403	0.456	0.536
CD80 on CD62L+ myeloid dendritic cell	0.696	0.241	0.279	0.296

MR, Mendelian randomization; IVW, inverse-variance weighting.

### Multivariable MR analysis

**Figure 2:**
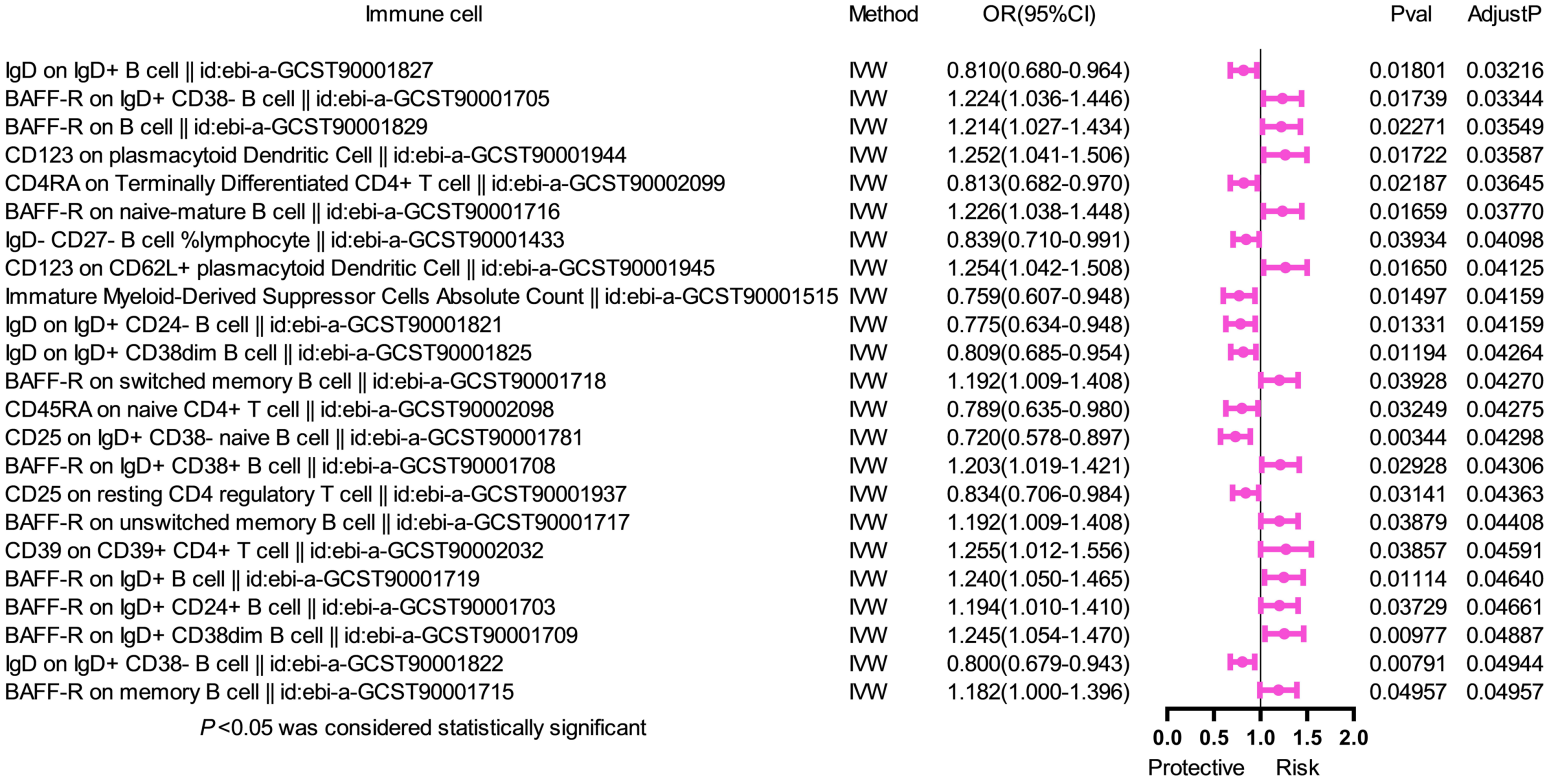
Forest plot of multivariable MR analysis between six identified immune cells and Sjögren’s syndrome. Multivariate MR analysis showed that three immunophenotypes remained statistically significant, while the other phenotypes lost significance. Two immunophenotypes (CD27 on IgD+ CD24+ B cell and CD80 on CD62L+ myeloid dendritic cell) were risk factors, and one immunophenotype (CD38 on IgD+ CD38+ B cell) was a protective factor.

To further assess the independent causal effect of immune cells on SS, we conducted a multivariate MR analysis (Figure 2). We found that only three immune cells exerted independent effects for SS: CD27 on IgD+ CD24+ B cell (OR = 1.111, 95%CI 1.038–1.189, *p* = 0.002); CD80 on CD62L+ mDC (OR = 1.105, 95%CI 1.034–1.181, *p* = 0.003) increased the risk of SS; and CD38 on IgD+ CD38+ B cell (OR = 0.893, 95%CI 0.818–0.975, *p* = 0.011) reduced the risk of SS. Remarkably, our results of multivariate MR analysis were broadly consistent with previous analyses, indicating that our results were highly reliable.

### Exploration of the causal effect of SS on immune cells

**Figure 3:**
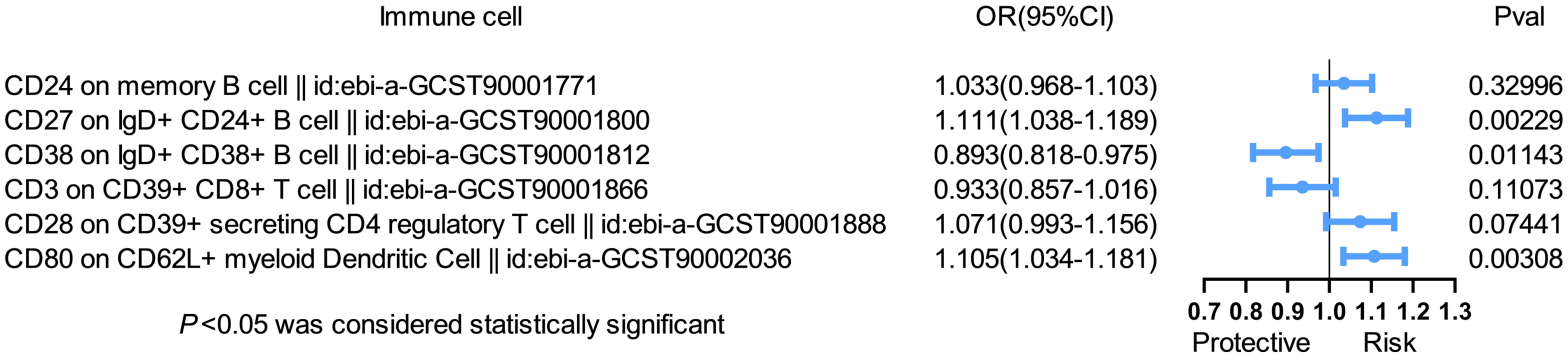
Forest plot with MR analysis showed that SS affected 25 immune cells. MR analysis showed that SS affected 25 immune cells. After correcting for FDR, 23 immune cells, belonging to B cell (16 cells), Treg (2 cells), cDC (2 cells), the maturation stage of T cells (2 cells), and myeloid cells (1 cell) were identified to have a reverse causal relationship with SS.

We conducted the MR analysis to explore the causal effect of SS on immune cells. We found that 25 immune cells had important relationships with SS. After correcting for FDR, 23 immune cells (Figure 3) were identified to have reverse causal associations with SS, including B cell (16 cells), Treg (2 cells), cDC (2 cells), the maturation stage of T cells (2 cells), and mDC (1 cell). However, we did not observe a bidirectional causal relationship between immune cells and SS. SS was a risk factor for 13 types of immune cells, including BAFF-R on IgD+ CD38- B cell (OR = 1.224, 95%CI 1.036–1.446, *p* = 0.017, *P*_FDR_ = 0.033), BAFF-R on B cell (OR = 1.214, 95%CI 1.027–1.434, *p* = 0.023, *P*_FDR_ = 0.035), CD123 on pDC (OR = 1.252, 95%CI 1.041–1.506, *p* = 0.017, *P*_FDR_ = 0.036), BAFF-R on naive-mature B cell (OR = 1.226, 95%CI 1.038–1.448, *p* = 0.017, *P*_FDR_ = 0.038), CD123 on CD62L+ pDC (OR = 1.254, 95%CI 1.042–1.508, *p* = 0.017, *P*_FDR_ = 0.041), BAFF-R on switched memory B cell (OR = 1.192, 95%CI 1.009–1.408, *p* = 0.039, *P*_FDR_ = 0.043), BAFF-R on IgD+ CD38+ B cell (OR = 1.203, 95%CI 1.019–1.421, *p* = 0.029, *P*_FDR_ = 0.043), BAFF-R on unswitched memory B cell (OR = 1.192,95%CI 1.009–1.408, *p* = 0.039, P_FDR_ = 0.044), CD39 on CD39+ CD4+ T cell (OR = 1.255,95%CI 1.012–1.556, *p* = 0.039, *P*_FDR_ = 0.046), BAFF-R on IgD+ B cell (OR = 1.240,95%CI 1.050–1.465, *p* = 0.011, *P*_FDR_ = 0.046), BAFF-R on IgD+ CD24+ B cell (OR = 1.194,95%CI 1.010–1.410, *p* = 0.037, *P*_FDR_ = 0.047), BAFF-R on IgD+ CD38dim B cell (OR = 1.245,95%CI 1.054–1.470, *p* = 0.010, *P*_FDR_ = 0.049), and BAFF-R on memory B cell (OR = 1.182,95%CI 1.000–1.396, *p* = 0.050, *P*_FDR_ = 0.050). In addition, SS was a protective factor for 10 types of immune cells, including IgD on IgD+ B cell (OR = 0.810, 95%CI 0.680–0.964, *p* = 0.018, *P*_FDR_ = 0.032), CD4RA on terminally differentiated CD4+ T cell (OR = 0.813, 95%CI 0.682–0.970, *p* = 0.022, *P*_FDR_ = 0.036), IgD- CD27- B cell %lymphocyte (OR = 0.839,95%CI 0.710–0.991, *p* = 0.039, *P*_FDR_ = 0.041), immature myeloid-derived suppressor cells absolute count (OR = 0.759,95%CI 0.607–0.948, *p* = 0.015, *P*_FDR_ = 0.042), IgD on IgD+ CD24- B cell (OR = 0.775,95%CI 0.634–0.948, *p* = 0.013, *P*_FDR_ = 0.042), IgD on IgD+ CD38dim B cell (OR = 0.809,95%CI 0.685–0.954, *p* = 0.012, *P*_FDR_ = 0.043), CD45RA on naive CD4+ T cell (OR = 0.789,95%CI 0.635–0.980, *p* = 0.032, *P*_FDR_ = 0.043), CD25 on IgD+ CD38- naive B cell (OR = 0.720,95%CI 0.578–0.897, *p* = 0.003, *P*_FDR_ = 0.043), CD25 on resting CD4 regulatory T cell (OR = 0.834,95%CI 0.706–0.984, *p* = 0.031, *P*_FDR_ = 0.044), and IgD on IgD+ CD38-B cell (OR = 0.800,95%CI 0.679–0.943, *p* = 0.008, *P*_FDR_ = 0.049). In the same way, the results of the other four methods were similar to those of the IVW method (Supplementary material S2). Furthermore, we found no evidence of heterogeneity and pleiotropy via Cochran’s Q-test and MR-Egger intercept analysis (Table 3), so we could assume that there was no horizontal pleiotropy. Finally, the leave-one-out test suggested that the result of reverse MR analysis was also stable (Supplementary material S2).

**Table 3:** Sensitivity analysis results of causal effects of Sjögren’s syndrome on immune cells.

Immune cell	Egger intercept	Cochran’s Q-test	
		MR-Egger	IVW
IgD on IgD+ B cell	0.655	0.202	0.330
BAFF-R on IgD+ CD38- B cell	0.601	0.990	0.769
BAFF-R on B cell	0.451	0.611	0.445
CD123 on plasmacytoid dendritic cell	0.939	0.668	0.908
CD4RA on terminally differentiated CD4+ T cell	0.678	0.607	0.752
BAFF-R on naive-mature B cell	0.464	0.535	0.441
IgD- CD27- B cell % lymphocyte	0.922	0.882	0.982
CD123 on CD62L+ plasmacytoid dendritic cell	0.952	0.634	0.890
Immature myeloid-derived suppressor cells’ absolute count	0.581	0.826	0.723
IgD on IgD+ CD24- B cell	0.642	0.144	0.225
IgD on IgD+ CD38dim B cell	0.644	0.313	0.491
BAFF-R on switched memory B cell	0.631	0.791	0.779
CD45RA on naive CD4+ T cell	0.798	0.090	0.204
CD25 on IgD+ CD38- naive B cell	0.858	0.165	0.363
BAFF-R on IgD+ CD38+ B cell	0.666	0.930	0.843
CD25 on resting CD4 regulatory T cell	0.722	0.552	0.751
BAFF-R on unswitched memory B cell	0.644	0.905	0.816
CD39 on CD39+ CD4+ T cell	0.889	0.104	0.256
BAFF-R on IgD+ B cell	0.445	0.572	0.419
BAFF-R on IgD+ CD24+ B cell	0.597	0.878	0.755
BAFF-R on IgD+ CD38dim B cell	0.429	0.676	0.419
IgD on IgD+ CD38- B cell	0.627	0.466	0.615
BAFF-R on memory B cell	0.594	0.954	0.759

MR, Mendelian randomization; IVW, inverse-variance weighting.

## Discussion

In our study, we conducted MR to investigate the causal relationship between 731 immune cells and SS. In this study, we found a strong causal relationship between six immune cells for SS (*P*_FDR_ < 0.05) and 23 immune cells for SS (*P*_FDR_ < 0.05). Our results provide further insight into the causal relationship between immune cells and SS. As far as we know, this is the first univariate, bivariate, and bidirectional MR analysis performed to investigate the link between immune cells and SS.

We observed that two types of T cells were significantly associated with the risk of SS, with elevated CD28 on CD39+ secreting CD4 regulatory T cells increasing the risk of SS, and elevated CD3 on CD39+ CD8+ T cells decreasing the risk of SS. CD39 plays an important role in the immune system. CD39+ CD8+ T cells inhibit the production of IFN-γ by CD39- CD8+ T cells through paracrine secretion of adenosine, which operates through the A2A receptor (16). Early disturbance of the innate immune barrier involving the IFN pathway in SS disease is associated with the etiology of SS (6). IFN-γ induces salivary gland epithelial cell ferroptosis in SS (17). An increase in the number of CD39+ CD8+ T cells found in Crohn’s patients is correlated with enhanced signal transduction of reactive oxygen species (ROS) (16). The ROS/pSTAT4/important protein aquaporin 5 axis affects salivary dysfunction in SS (18). Although some studies have focused on Tregs and SS, the role of Tregs in the occurrence and development of SS remains elusive. Sarigul et al. reported that the increase of Foxp3+ Treg cells in the peripheral blood of SS patients was positively correlated with a higher grade of infiltration at the salivary glands (19). Alunno et al. reported an expansion of CD4+ CD25- GITR+ regulatory T cell subsets in the peripheral blood of patients with primary SS (pSS)’, which was correlated with the degree of disease activity (20). The correlation between CD28 on CD39+ secreting CD4 regulatory T cell and SS has not been clarified at present. In other autoimmune diseases, an increase in peripheral CD39-expressing T regulatory cells has been associated with relapsing–remitting multiple sclerosis (21). In addition, in patients with type 2 diabetes, elevated CD39+ Treg cells are associated with hyperglycemia, overweight, and obesity (22). The collaboration of CD39 and CD73 results in the conversion of ATP to ADP and adenosine 5′-monophosphate (cAMP), ultimately generating adenosine (23, 24). Adenosine plays an immunosuppressive role when interacting with A2A and A2B receptors but stimulates an immune response when interacting with A1 and A3 receptors (25–27). These findings may be the reason why CD28 on CD39+ secreting CD4 regulatory T cell is a risk factor for SS, whereas CD3 on CD39+ CD8+ T cell plays a protective role in SS. However, further experiments are needed to clarify the relevant mechanisms. cAMP can activate the mitogen-activated protein kinase (MAPK) pathway, thereby inhibiting nicotinamide adenine dinucleotide phosphate oxidase and alleviating TGF-B1-induced salivary gland fibrosis. CD28 on CD39+ secreting CD4 regulatory T cell and CD3 on CD39+ CD8+ T cell may affect the pathogenesis and development of SS through adenosine (and its derivatives) or IFN, which needs to be clarified by validation and functional evaluation experiments.

In addition, our results showed that three phenotypes of B cells were associated with SS risk. Elevations of CD24 on memory B cells and CD27 on IgD+ CD24+ B cells increased the risk of SS, while CD38 on IgD+ CD38+ B cells decreased the risk of SS. Polyclonal over-proliferation of B cells is one of the immunological features of SS (9). CD24 expression on pro-B cells plays a role in the selection and development of B cells in bone marrow. In memory B cells, there was a strong positive correlation between CD24 expression and phosphorylation flow (phosphorylation of AMPK-pAMPK), especially in IgD+ IgM+ memory B cells (28). MAPKs are downstream of many immune and cytokine receptors, such as toll-like receptors, interleukin (IL)-1R, tumor necrosis factor receptor, colony-stimulating factor 1 receptor, IL-17R, epidermal cell growth factor, fibroblast growth factor, and vascular endothelial growth factor (29). These immune and cellular factors are involved in the occurrence or development of SS and its complications, to varying degrees (30–34).

Peripheral blood IgD+ CD38+ B cells, also known as naïve B cells, were significantly higher in SS patients than in healthy donors and higher in women than men (35, 36). An increased proportion of CD38 high IgD+ B cells in pSS is involved in IgG overproduction, including autoantibodies, and correlates with disease progression (37). In patients with pSS treated with iguratimod, CD38+ IgD+ B cells and BAFF-R were significantly reduced, while disease activity scores were decreased (38). This is not consistent with our findings, suggesting that IgD+ CD38+ B cells reduce the risk of SS, but the results of MR analysis could not fully reveal the relationship between immune cells and SS and need to be confirmed in combination with experiments. It is also possible that interference between immune cells causes their true role in SS to be obscured.

Furthermore, our results showed that the elevation of CD80 on CD62L+ mDCs was positively correlated with the risk of SS and remained significantly correlated after adjusting for multivariate MR. mDCs undergo many activities that contribute to the initiation of immunity. There are two main subtypes of human DCs: mDCs and pDCs. pDCs primarily drive innate inflammatory responses to pathogens by secreting large amounts of IFN-alpha (IFNα), whereas mDCs are specifically used for antigen presentation to guide adaptive responses (39, 40). CD80 on CD62L+ is the more mature mDCs phenotype, and mature DCs are stimulators of T cell immune response, whereas immature DCs support T cell tolerance (41). Compared to healthy controls, pDC and mDC2 in the peripheral blood of pSS patients were significantly reduced (40). Compared to non-S’S dry eye and healthy volunteers, SS’ dry eye showed significantly higher DC density, larger DC size, and more DC dendrites with a larger DC field. Moreover, DC density and morphological parameters were significantly correlated with the degree of salivary gland pathology, serum antibody titer, and ocular surface damage (42). However, there is no literature on the change in CD80 on CD62L+ mDC expression level in SS, which is worthy of further exploration.

Our results showed that SS was a risk factor or protective factor for 23 immunophenotypes, including 16 B cells, 4 T cells, 2 cDC cells, and 1 mDC. Our results confirm that SS is a B cell-associated disease at the genetic level. BAFF is a member of the TNF superfamily by binding to the transmembrane activator, calcium modulator, and cyclophilin ligand interactor or BAFF-R on B cells. BAFF supports B cell development, differentiation, and survival, especially for plasma and plasma cells, and plays a key role in the pathogenesis of B cell-associated autoimmune diseases. BAFF therapy targeting B cells in SS’ helps reduce disease activity in SS and restores normalization of B cell frequency, phenotype, and function (43, 44). The results of this study provide potential indicators and clues for the early diagnosis and activity assessment of SS, which can be further explored and validated in future clinical cases.

Considered together, our findings demonstrate that immune cells play a potential causal role in SS, providing an important auxiliary role for clarifying diagnosis and therapeutic strategies, as well as providing directions for the development of new drugs. Our MR analysis offered several advantages. First, we used univariate, multivariate, and bidirectional MR to mitigate confounding factors and reverse causation. Second, our study adopted five methods to ensure the reliability of the result, including MR-Egger, weighted median, IVW, weighted mode, and simple mode. At the same time, multiple methods were used to observe pleiotropy and heterogeneity of the results, such as the MR-PRESSO test, Cochran’s Q-test, MR-Egger intercept analysis, and leave-one-out test, to ensure that our MR results were robust and reliable, with no apparent bias from other sources of pleiotropy. However, our study has limitations. First, in this study, multifactor, bidirectional MR analysis was performed, and the results showed that six types of immune cells may be associated with the risk of SS. However, MR analysis could not reveal the causal relationship between immune cells and SS, but it provides new avenues for studying the mechanisms of SS. Further experiments are needed to evaluate and validate the function of the selected immune cells by analyzing patient clinical data and biological samples. Second, the GWAS data used in this study were derived from European populations, so may not be directly applicable to other populations. Third, GWAS data were currently unable to distinguish between pSS and secondary SS, which made it impossible to perform subgroup stratification analyses of the SS population. Fourth, the limited sample size could introduce bias, so a larger sample is needed to obtain reliable results.

## Conclusion

This study used univariate, multivariate, and bidirectional MR analysis to investigate the causal relationship between several immune cells and SS and to clarify that immune cells affect the progression of SS in a complex pattern. These findings improve our understanding of the interaction between immune cells and SS risk, providing new avenues for studying the prevention, diagnosis, and treatment of SS. Nevertheless, further experiments are needed to elucidate the underlying mechanisms.

## Data availability statement

The original contributions presented in the study are included in the article/Supplementary material, further inquiries can be directed to the corresponding author.

## Ethics statement

Ethical approval was not required for the study involving humans in accordance with the local legislation and institutional requirements. Written informed consent to participate in this study was not required from the participants or the participants’ legal guardians/next of kin in accordance with the national legislation and the institutional requirements.

## Author contributions

WZ: Conceptualization, Data curation, Formal analysis, Funding acquisition, Investigation, Methodology, Project administration, Resources, Software, Supervision, Validation, Visualization, Writing – original draft, Writing – review & editing. MH: Writing – original draft, Conceptualization, Data curation, Formal analysis, Funding acquisition, Investigation, Methodology, Project administration, Resources, Software, Supervision, Validation, Visualization. YZ: Conceptualization, Data curation, Formal analysis, Funding acquisition, Investigation, Methodology, Project administration, Resources, Software, Supervision, Validation, Visualization, Writing – review & editing. JP: Conceptualization, Software, Writing – original draft. FQ: Methodology, Project administration, Writing – original draft. XL: Conceptualization, Investigation, Writing – review & editing. LZ: Data curation, Formal analysis, Writing – original draft. LL: Conceptualization, Software, Writing – original draft, Writing – review & editing.

## Footnotes

^1^ https://gwas.mrcieu.ac.uk/datasets/finn-b-M13_SJOGREN/
