# Supplementary material for: The causal relationship between immune cells and Sjögren’s syndrome: a univariate, multivariate, bidirectional Mendelian randomized study
Source: Front Med (Lausanne). 2024 Jul 2;11:1408562. doi: 10.3389/fmed.2024.1408562 (PMC11249722; doi:10.3389/fmed.2024.1408562)
Supplement: Supplementary file 1 [file Data_Sheet_1.ZIP › CD123 on CD62L+ plasmacytoid Dendritic Cell.forest.pdf]

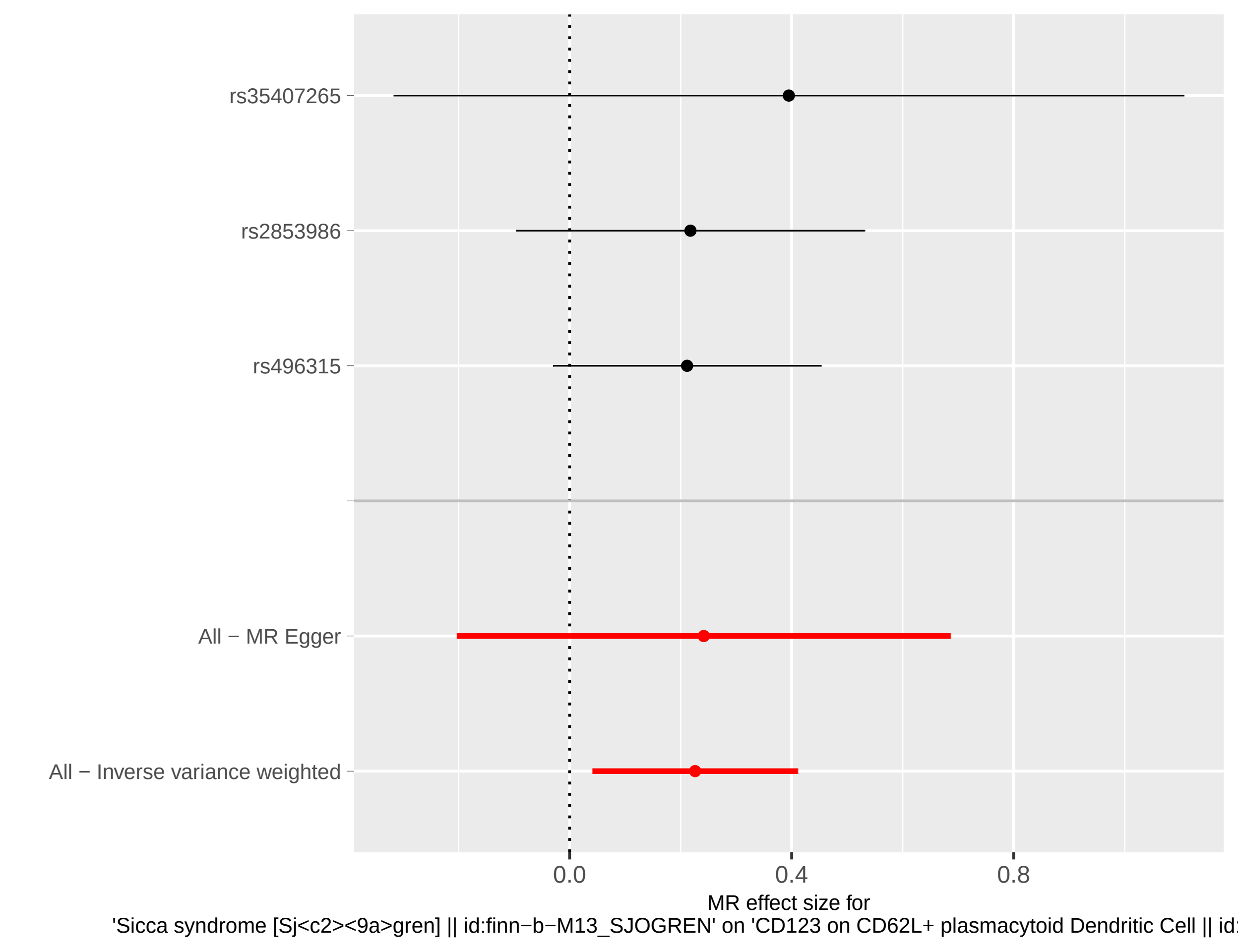

rs35407265
rs2853986
rs496315
All − MR Egger
All − Inverse variance weighted
0.0
0.4
0.8
MR effect size for
'Sicca syndrome [Sj<c2><9a>gren] || id:finn−b−M13_SJOGREN' on 'CD123 on CD62L+ plasmacytoid Dendritic Cell || id:
