# Supplementary material for: The causal relationship between immune cells and Sjögren’s syndrome: a univariate, multivariate, bidirectional Mendelian randomized study
Source: Front Med (Lausanne). 2024 Jul 2;11:1408562. doi: 10.3389/fmed.2024.1408562 (PMC11249722; doi:10.3389/fmed.2024.1408562)
Supplement: Supplementary file 1 [file Data_Sheet_1.ZIP › CD25 on resting CD4 regulatory T cell.scatter_plot.pdf]

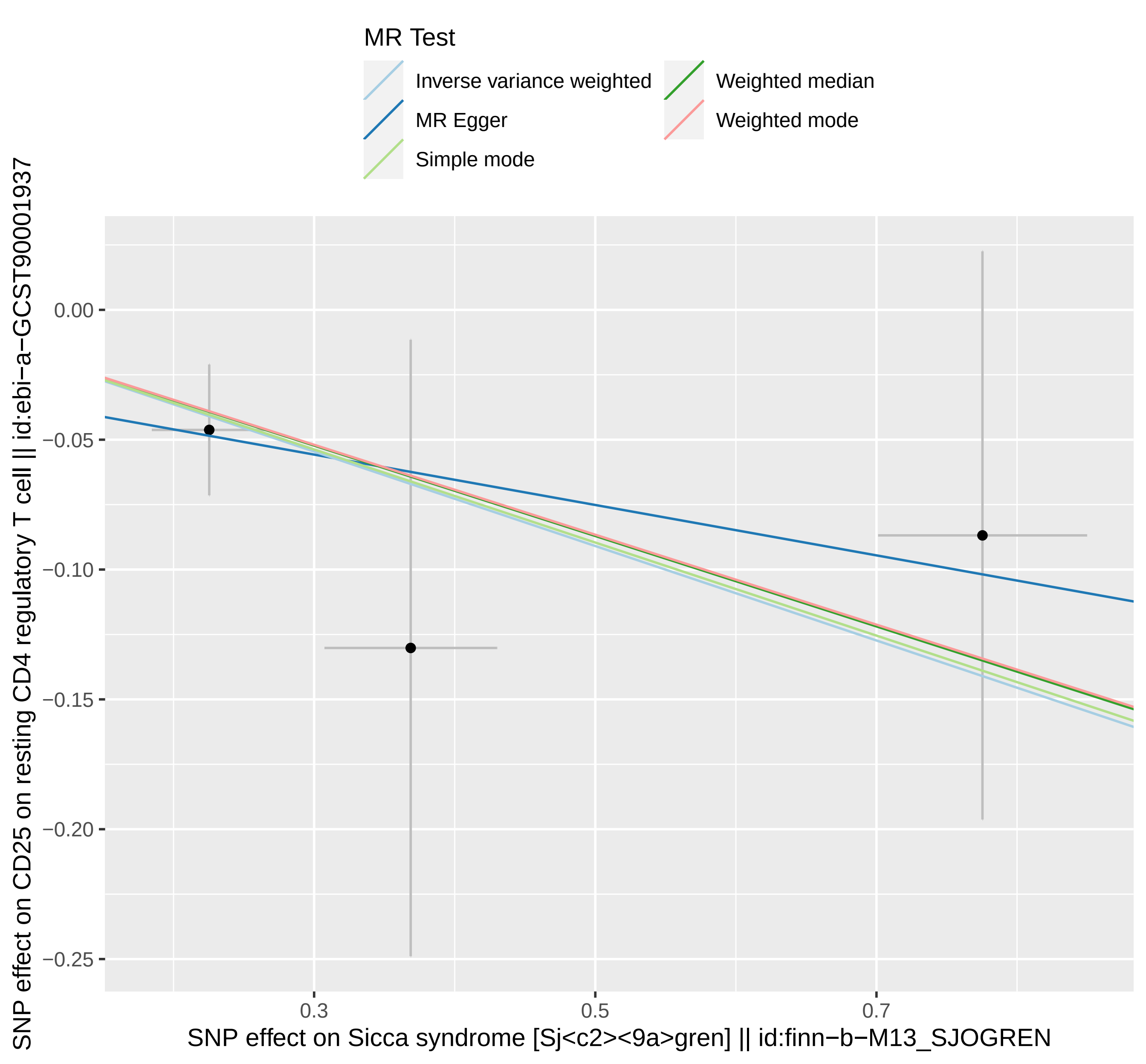
MR Test
Inverse variance weighted
MR Egger
Simple mode
Weighted median
Weighted mode
0.00
−0.05
−0.10
−0.15
−0.20
−0.25
0.3
0.5
0.7
SNP effect on CD25 on resting CD4 regulatory T cell || id:ebi−a−GCST90001937
SNP effect on Sicca syndrome [Sj<c2><9a>gren] || id:finn−b−M13_SJOGREN
