# Supplementary figures and images for: The causal relationship between immune cells and Sjögren’s syndrome: a univariate, multivariate, bidirectional Mendelian randomized study
Source: Front Med (Lausanne). 2024 Jul 2;11:1408562. doi: 10.3389/fmed.2024.1408562 (PMC11249722; doi:10.3389/fmed.2024.1408562)

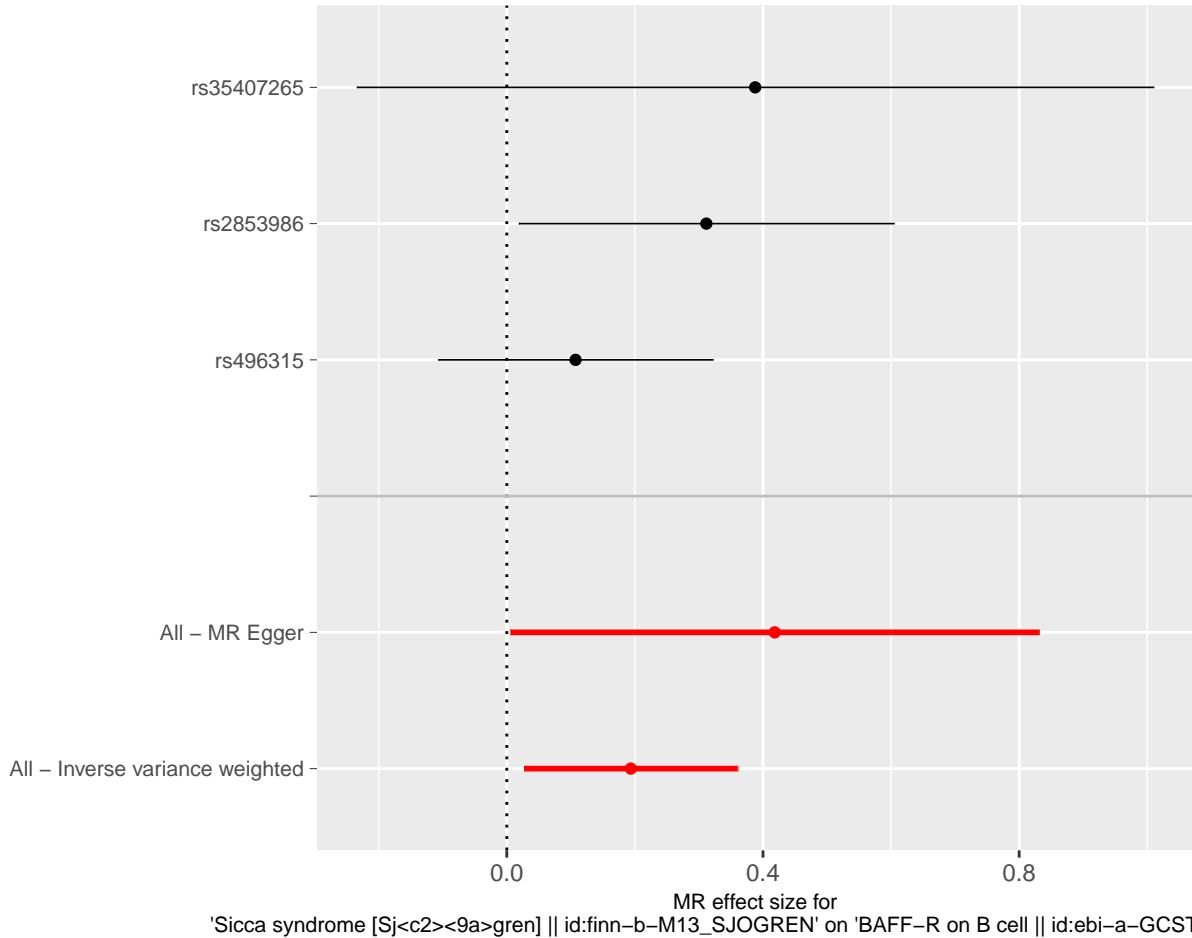

Supplement: Supplementary file 1 [file Data_Sheet_1.ZIP › BAFF-R on B cell.forest.pdf]

# MR Method

- Inverse variance weighted
- MR Egger

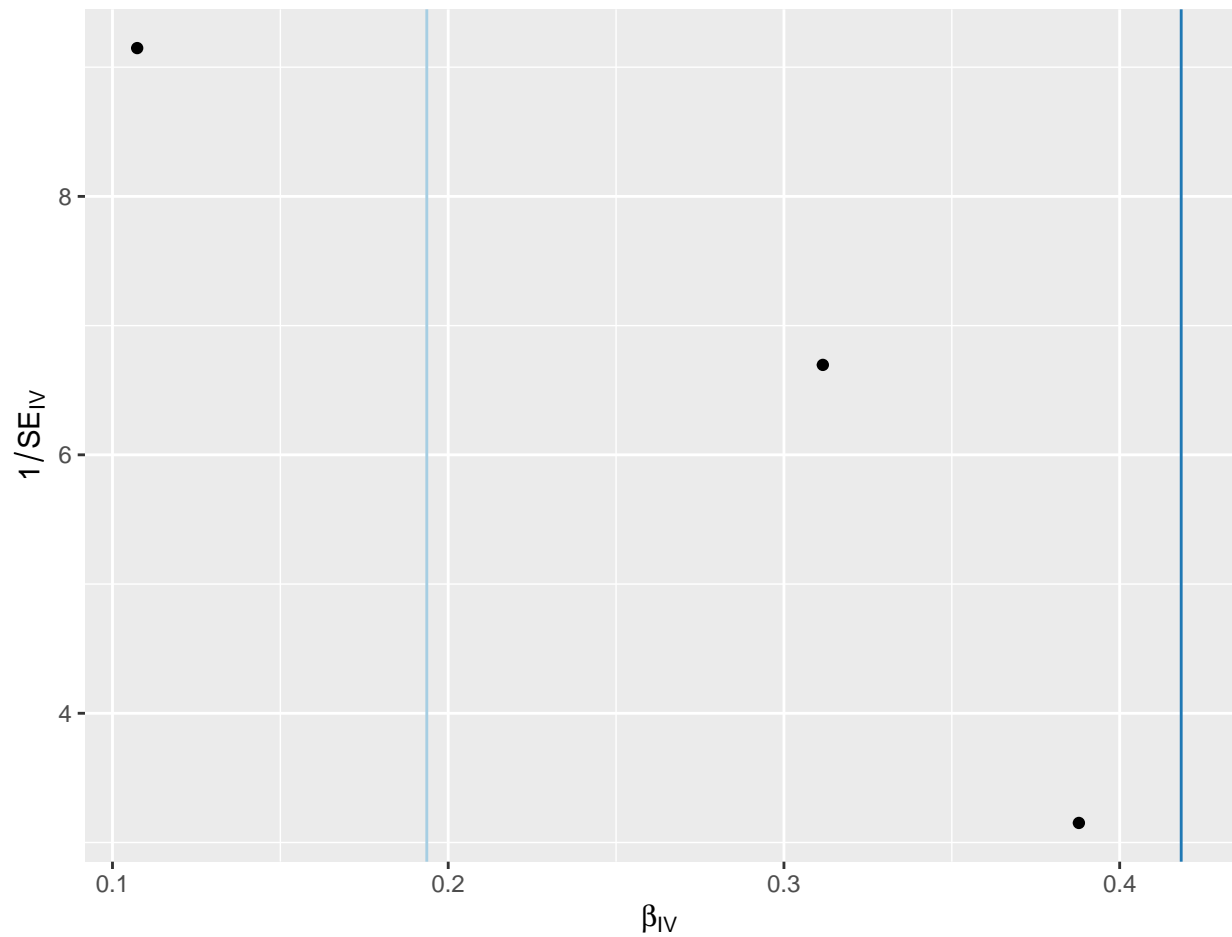

Supplement: Supplementary file 1 [file Data_Sheet_1.ZIP › BAFF-R on B cell.funnel_plot.pdf]

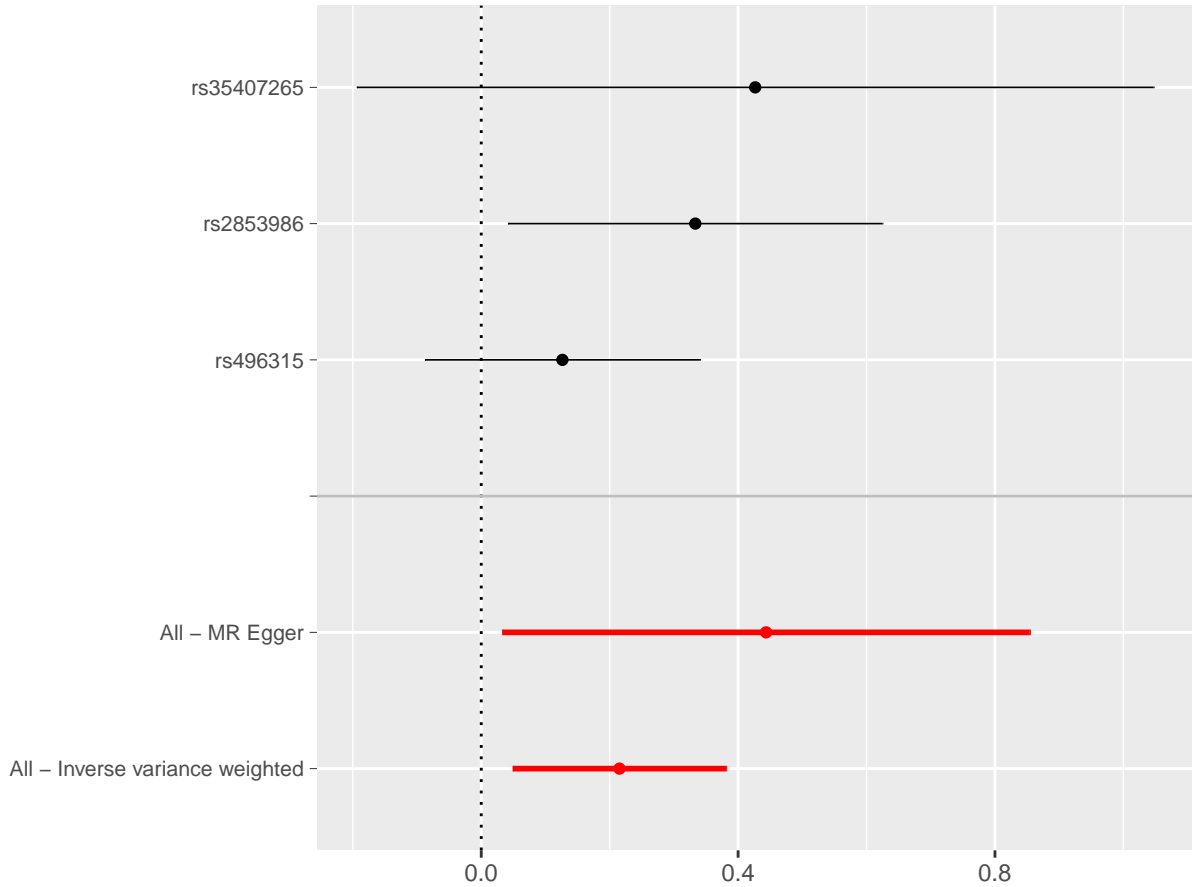

MR effect size for  
'Sicca syndrome [Sj<c2><9a>gren] || id:finn-b-M13\_SJOGREN' on 'BAFF-R on IgD+ B cell || id:ebi-a-GCS

Supplement: Supplementary file 1 [file Data_Sheet_1.ZIP › BAFF-R on IgD+ B cell.forest.pdf]

# MR Method

- Inverse variance weighted
- MR Egger

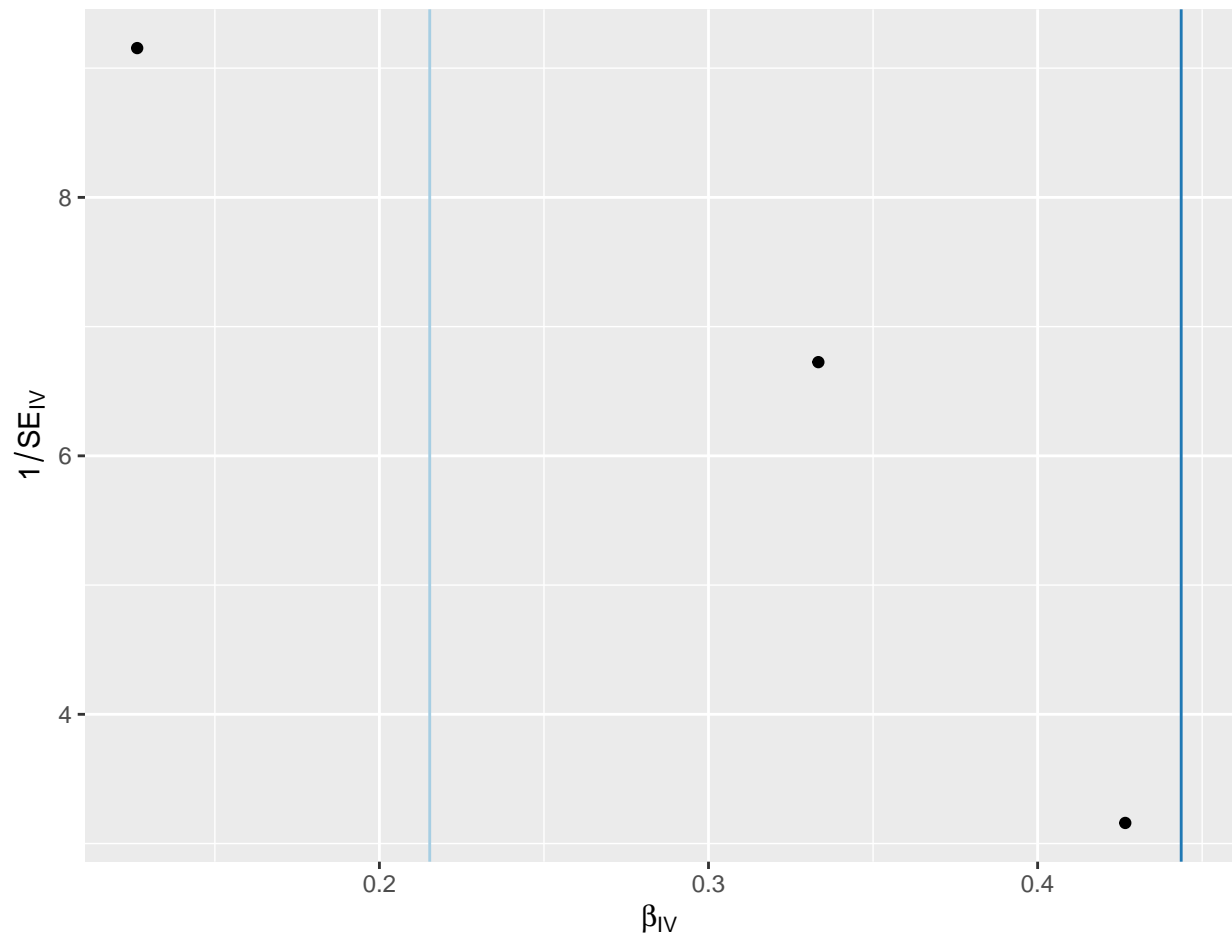

Supplement: Supplementary file 1 [file Data_Sheet_1.ZIP › BAFF-R on IgD+ B cell.funnel_plot.pdf]

# MR Test

- Inverse variance weighted
- MR Egger
- Simple mode
- Weighted median
- Weighted mode

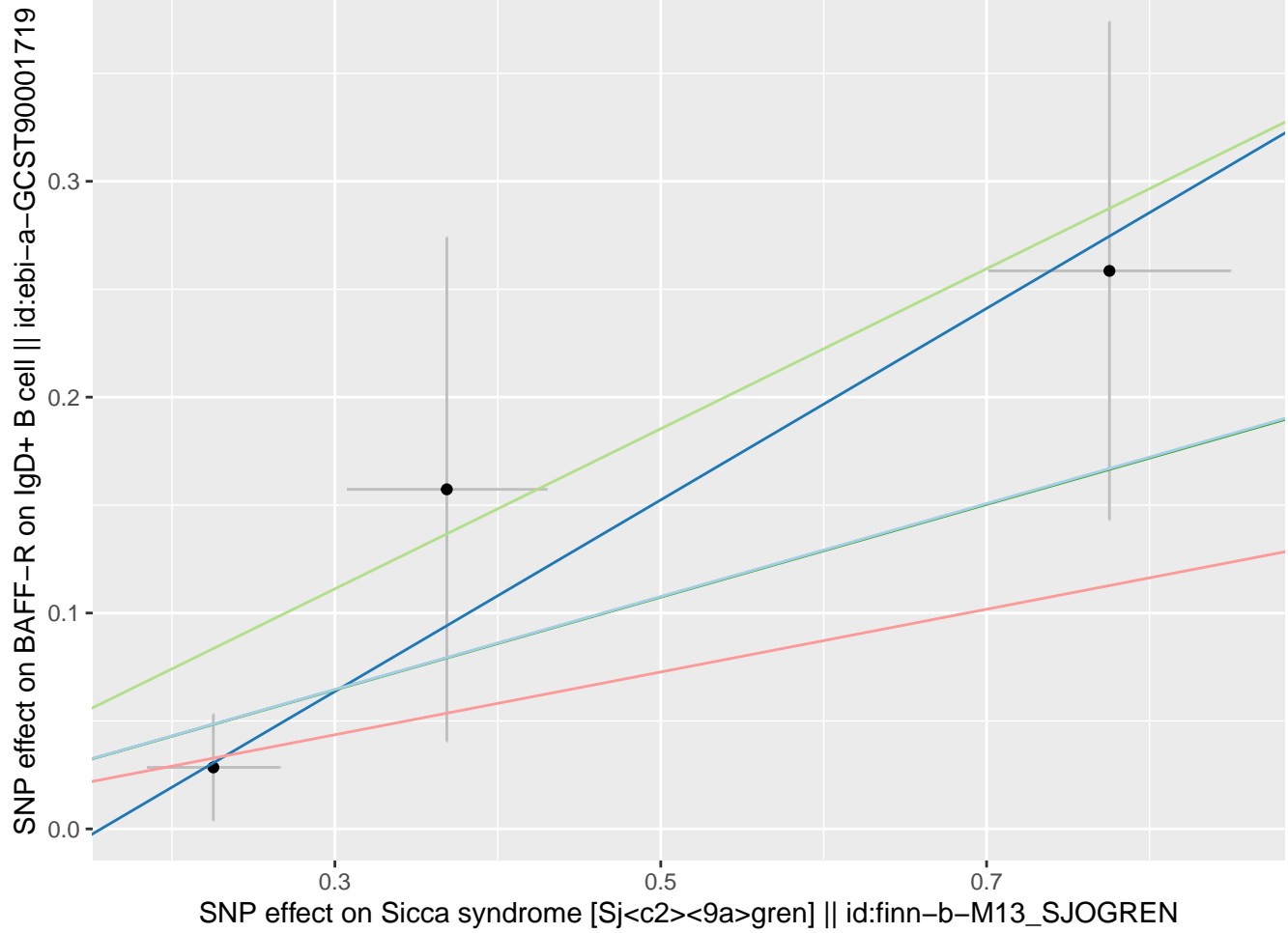

Supplement: Supplementary file 1 [file Data_Sheet_1.ZIP › BAFF-R on IgD+ B cell.scatter_plot.pdf]

# MR Method

- Inverse variance weighted
- MR Egger

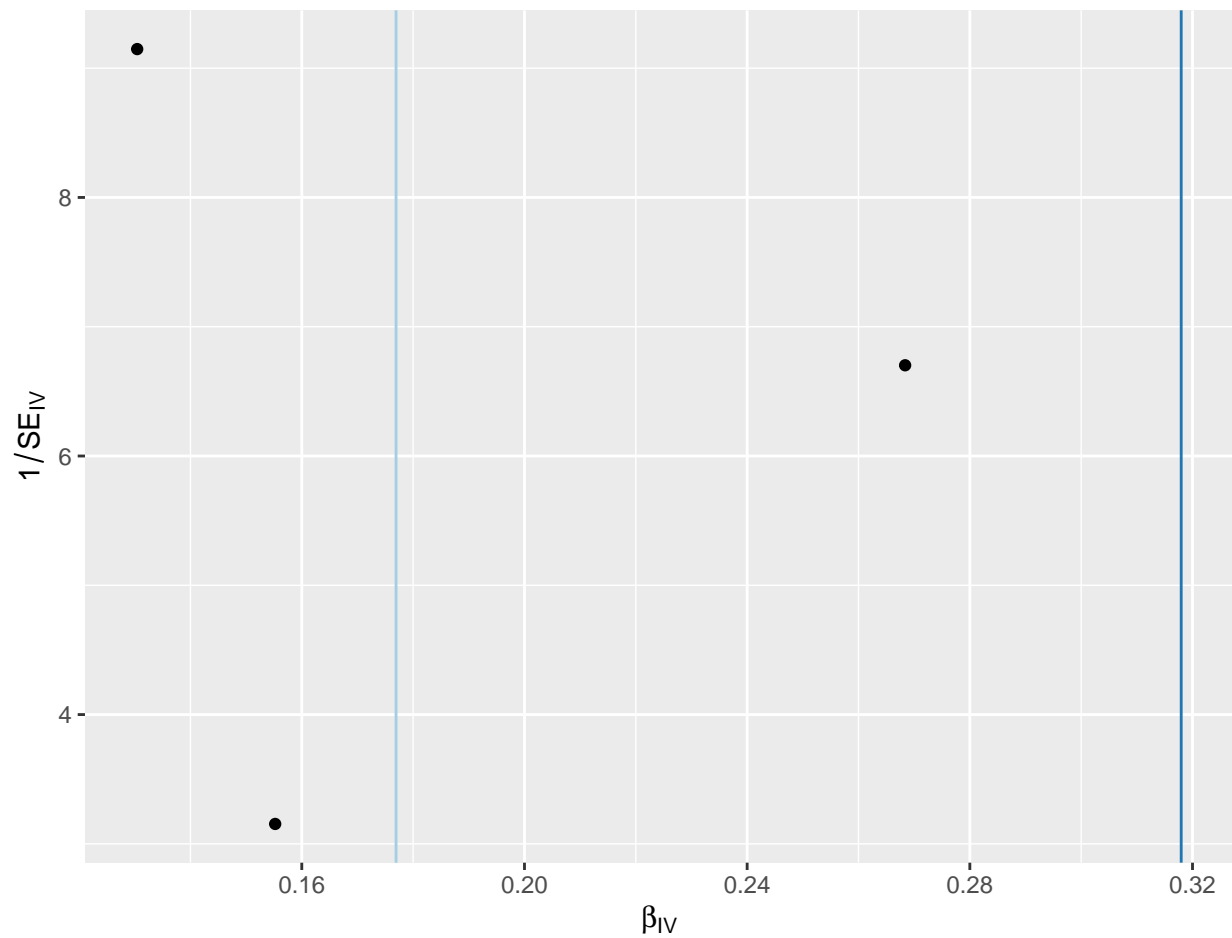

Supplement: Supplementary file 1 [file Data_Sheet_1.ZIP › BAFF-R on IgD+ CD24+ B cell.funnel_plot.pdf]

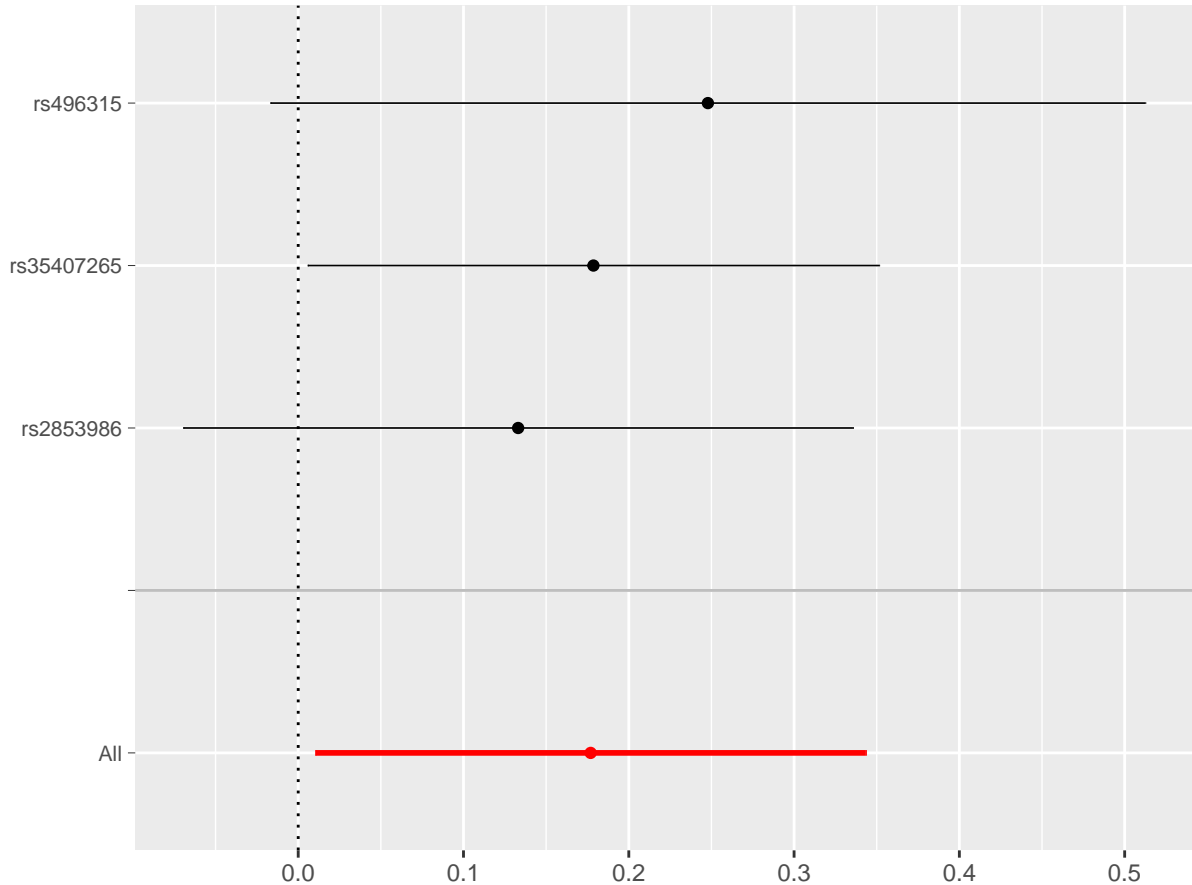

Supplement: Supplementary file 1 [file Data_Sheet_1.ZIP › BAFF-R on IgD+ CD24+ B cell.leaveoneout.pdf]

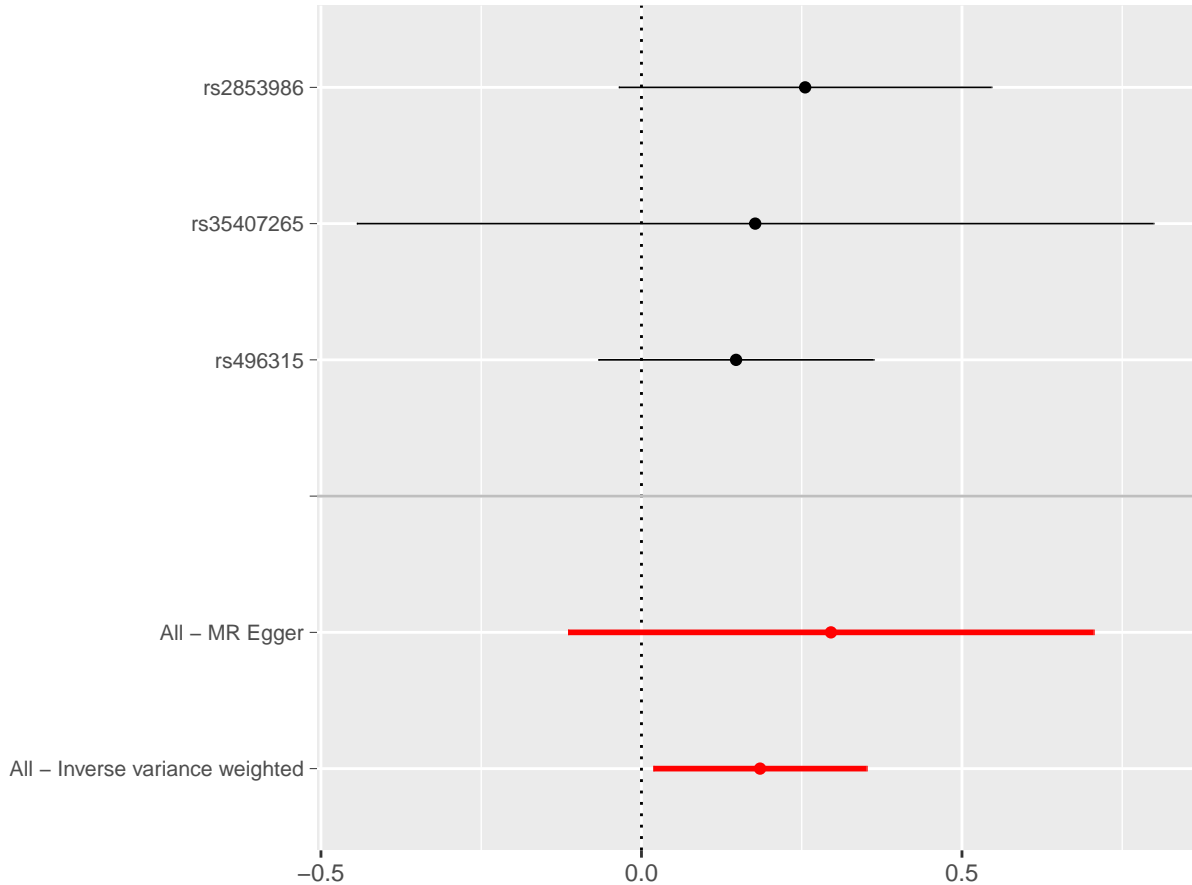

MR effect size for  
'Sicca syndrome [Sj<c2><9a>gren] || id:finn-b-M13\_SJOGREN' on 'BAFF-R on IgD+ CD38+ B cell || id:ebi-a-C

Supplement: Supplementary file 1 [file Data_Sheet_1.ZIP › BAFF-R on IgD+ CD38+ B cell.forest.pdf]

# MR Method

- Inverse variance weighted
- MR Egger

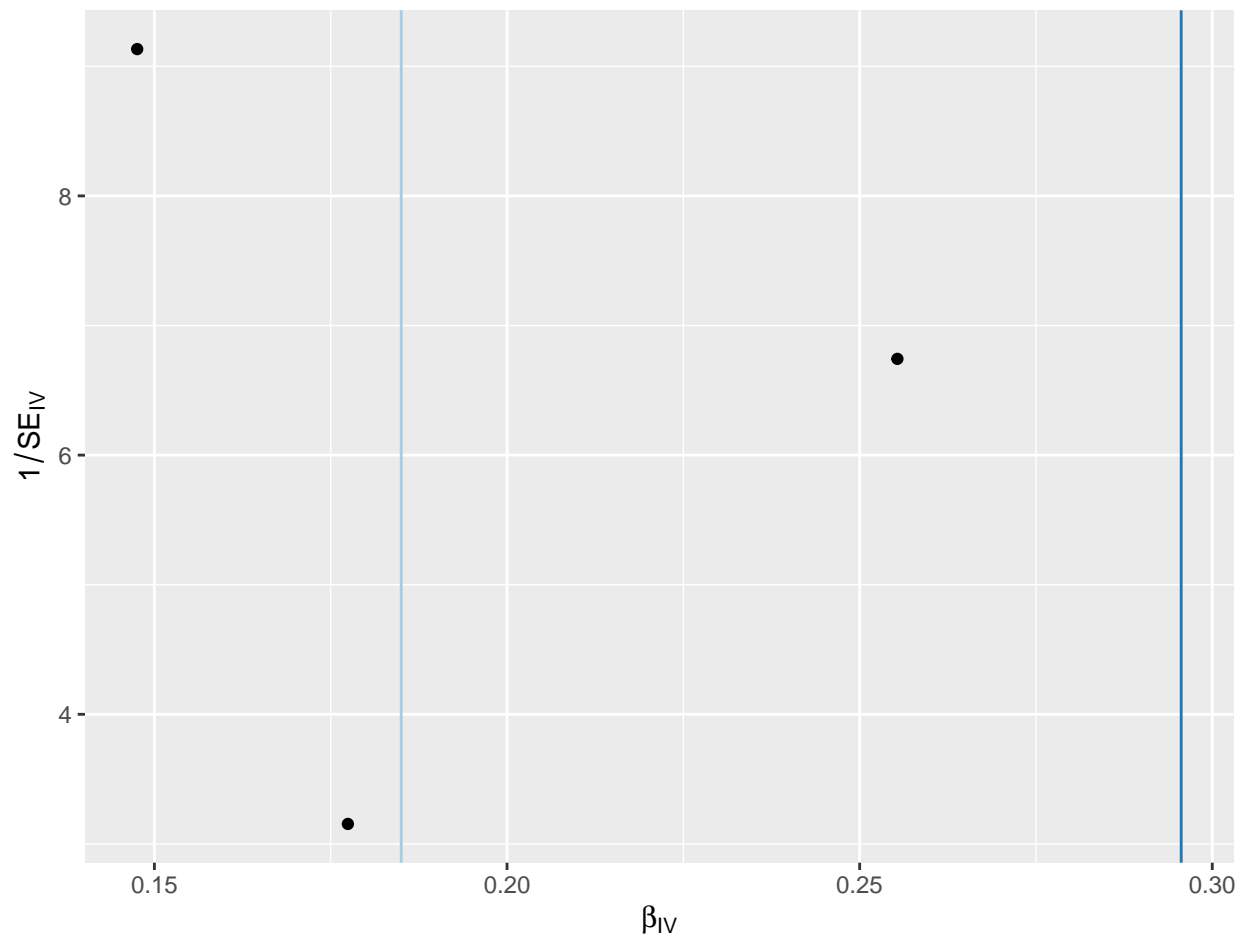

Supplement: Supplementary file 1 [file Data_Sheet_1.ZIP › BAFF-R on IgD+ CD38+ B cell.funnel_plot.pdf]

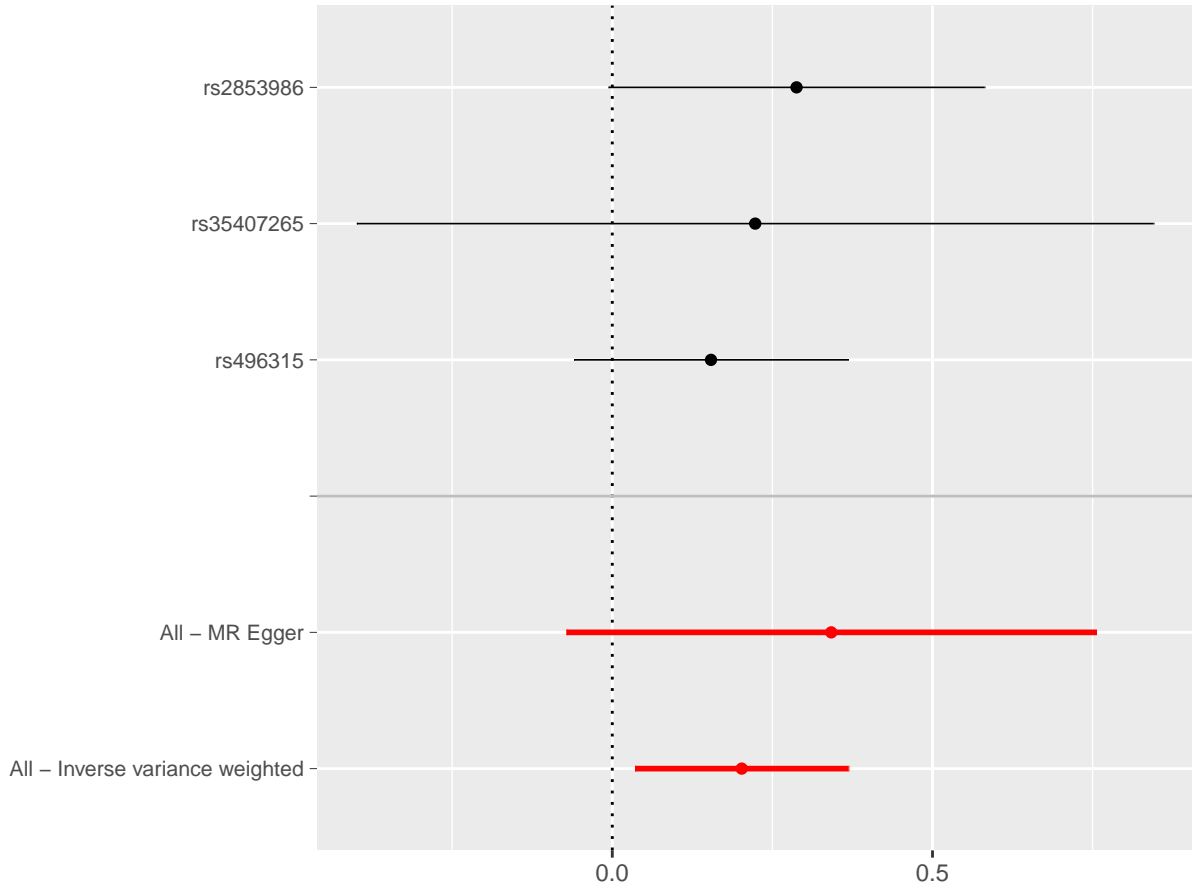

MR effect size for  
'Sicca syndrome [Sj<c2><9a>gren] || id:finn-b-M13\_SJOGREN' on 'BAFF-R on IgD+ CD38- B cell || id:ebi-a-C

Supplement: Supplementary file 1 [file Data_Sheet_1.ZIP › BAFF-R on IgD+ CD38- B cell.forest.pdf]

# MR Method

- Inverse variance weighted
- MR Egger

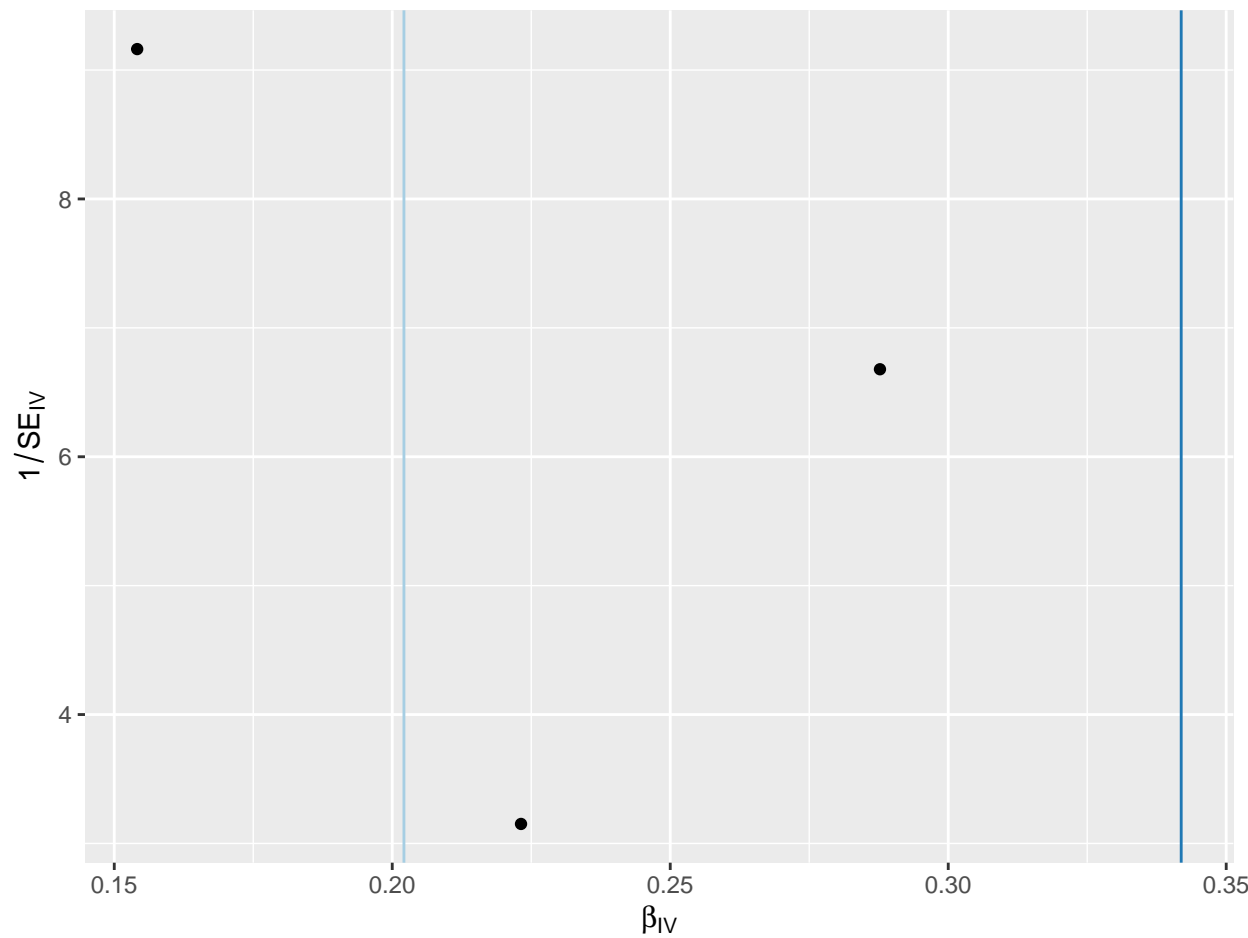

Supplement: Supplementary file 1 [file Data_Sheet_1.ZIP › BAFF-R on IgD+ CD38- B cell.funnel_plot.pdf]

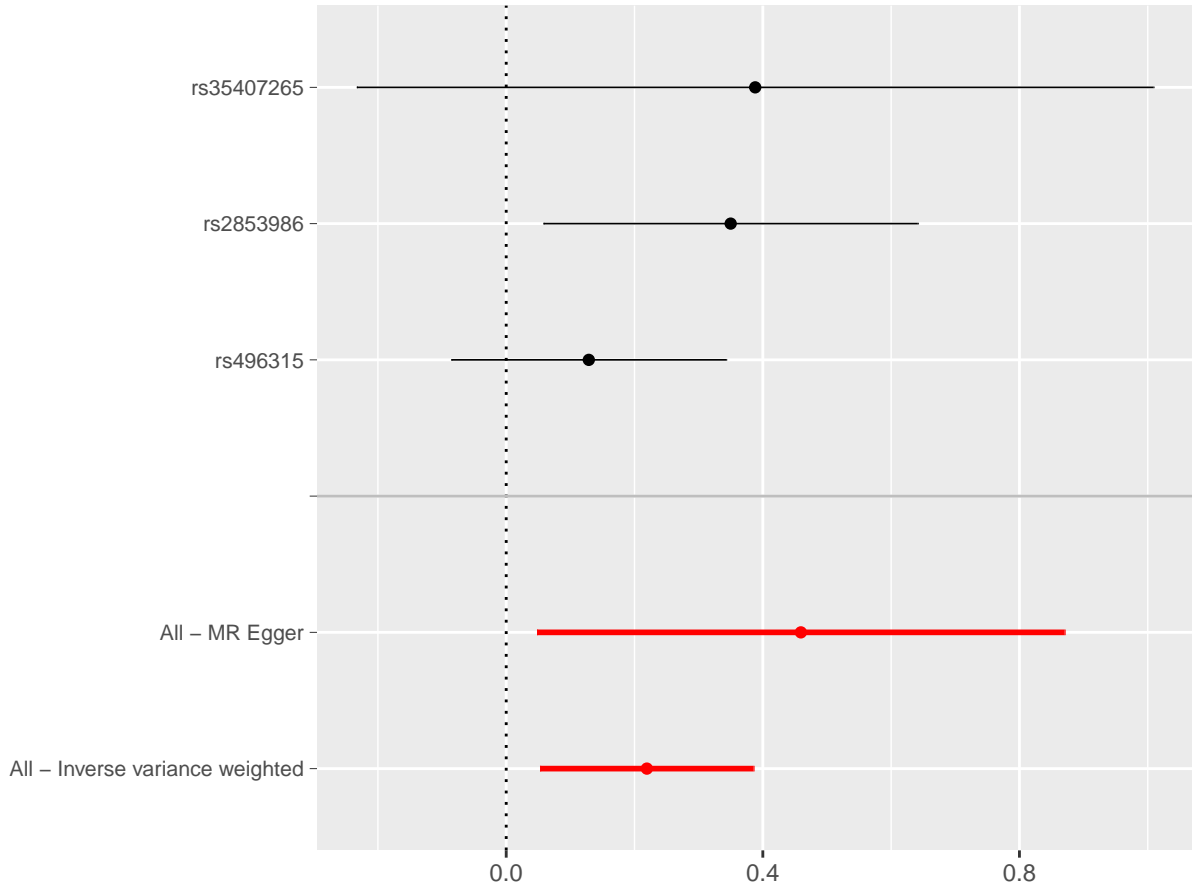

MR effect size for  
'Sicca syndrome [Sj<c2><9a>gren] || id:finn-b-M13\_SJOGREN' on 'BAFF-R on IgD+ CD38dim B cell || id:ebi-a-

Supplement: Supplementary file 1 [file Data_Sheet_1.ZIP › BAFF-R on IgD+ CD38dim B cell.forest.pdf]

# MR Method

- Inverse variance weighted
- MR Egger

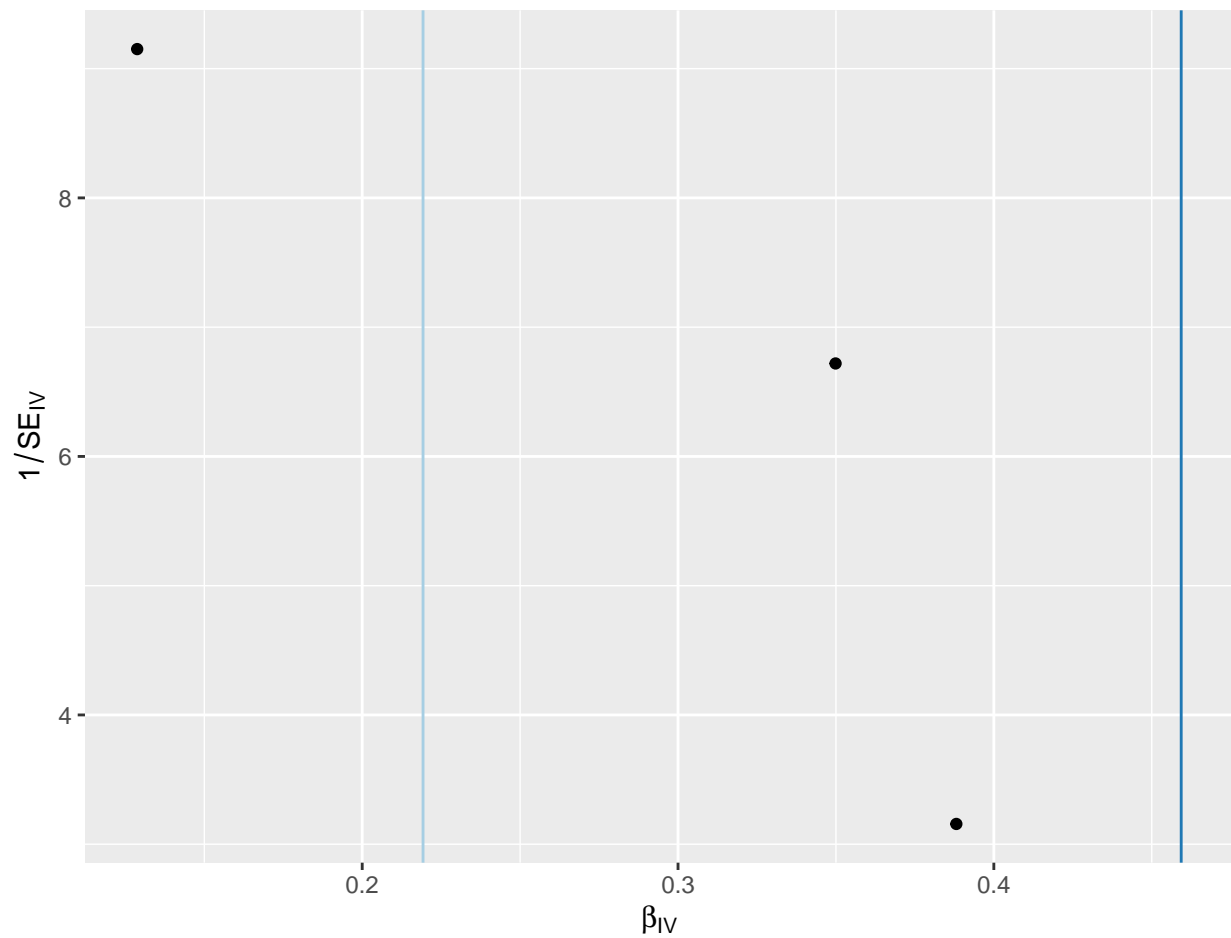

Supplement: Supplementary file 1 [file Data_Sheet_1.ZIP › BAFF-R on IgD+ CD38dim B cell.funnel_plot.pdf]

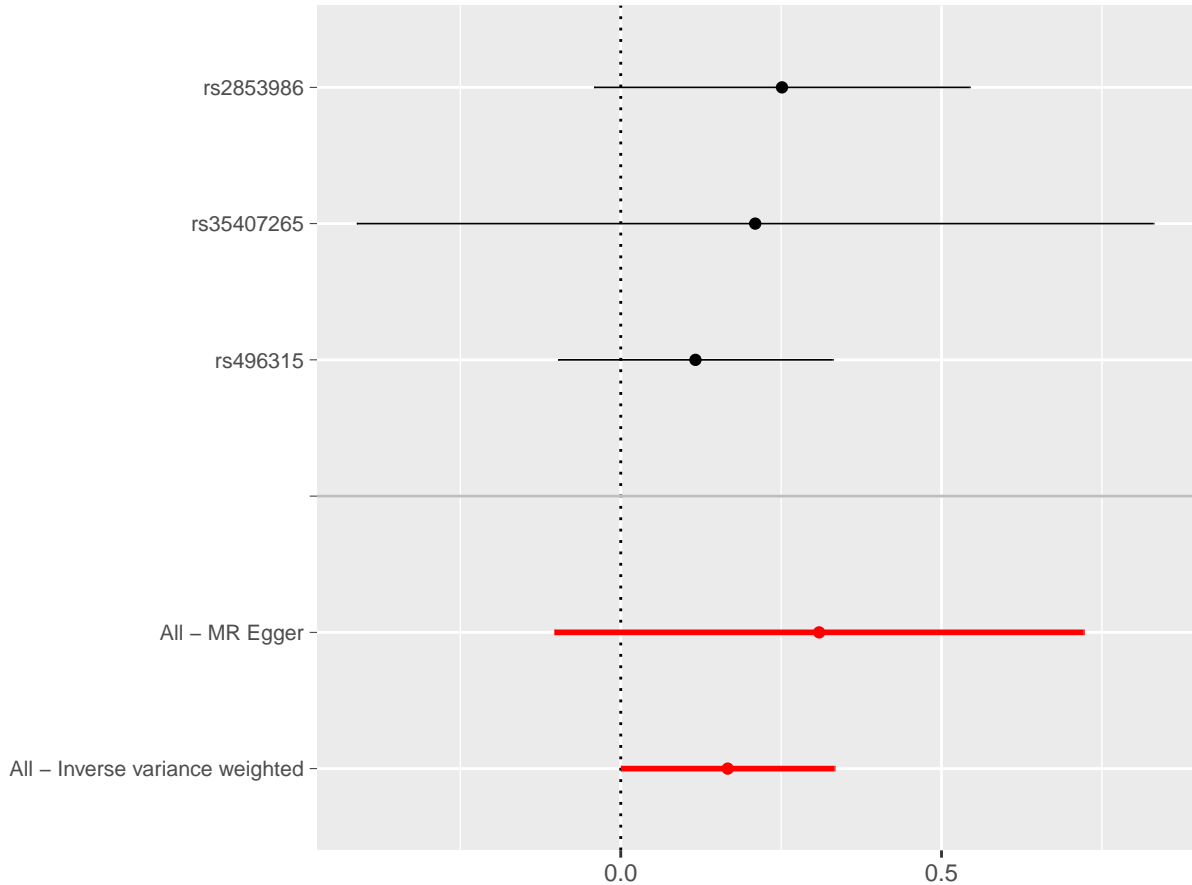

MR effect size for  
'Sicca syndrome [Sj<c2><9a>gren] || id:finn-b-M13\_SJOGREN' on 'BAFF-R on memory B cell || id:ebi-a-GC

Supplement: Supplementary file 1 [file Data_Sheet_1.ZIP › BAFF-R on memory B cell.forest.pdf]

# MR Method

- Inverse variance weighted
- MR Egger

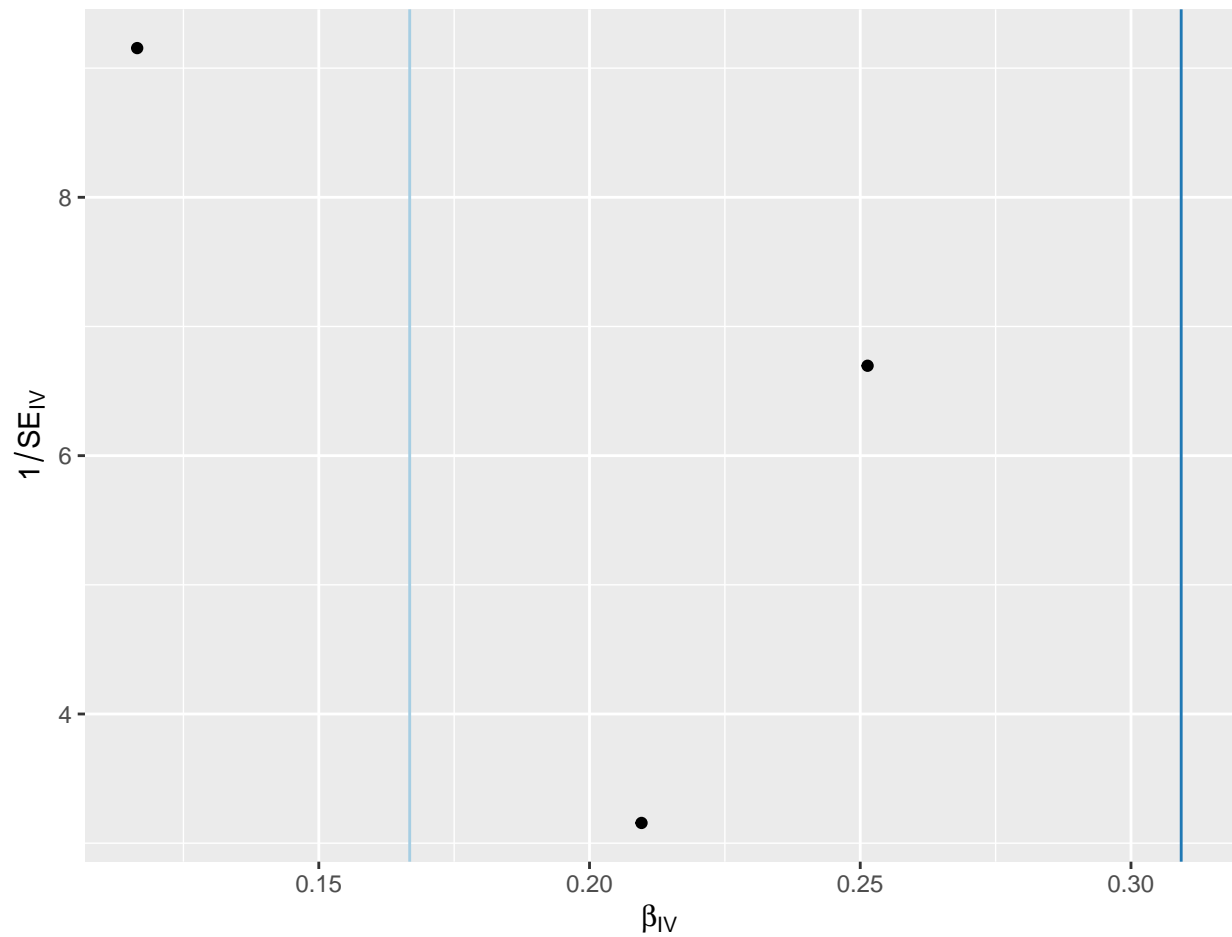

Supplement: Supplementary file 1 [file Data_Sheet_1.ZIP › BAFF-R on memory B cell.funnel_plot.pdf]

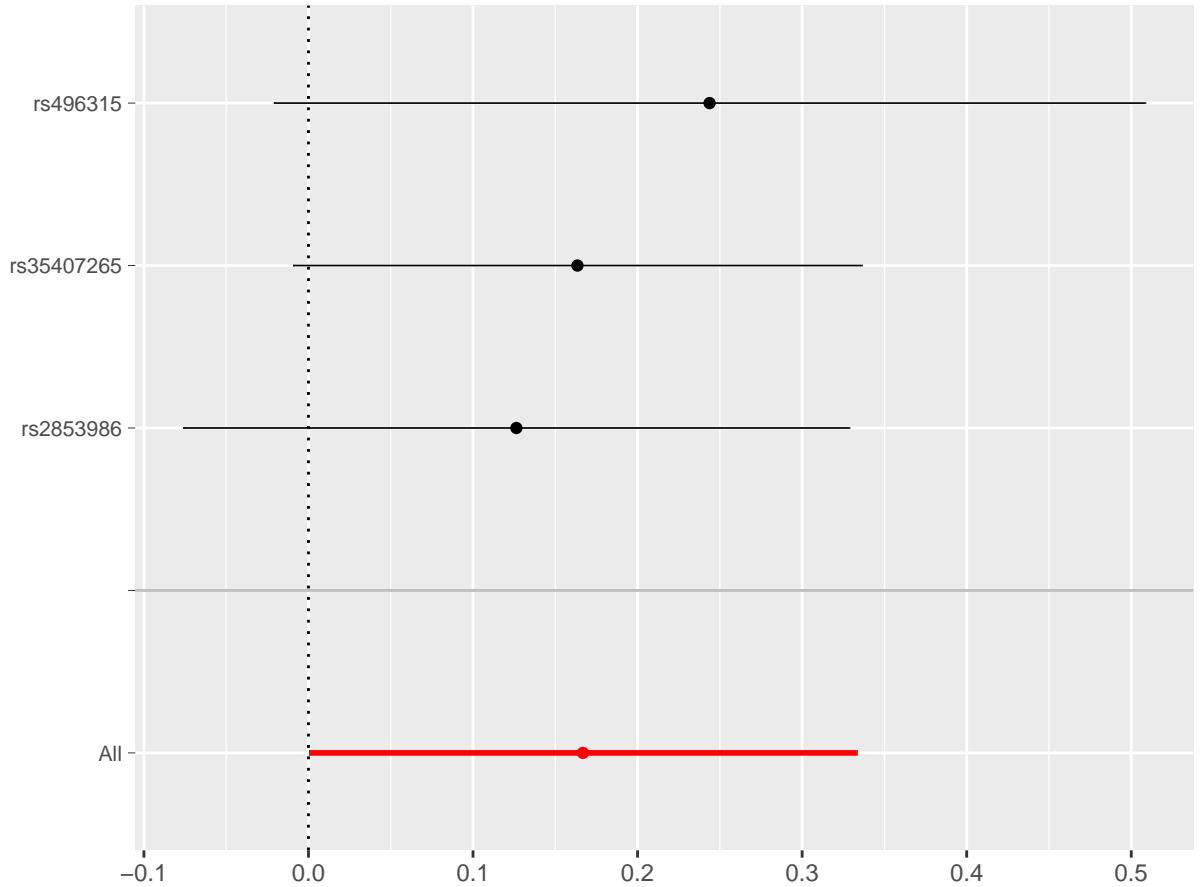

Supplement: Supplementary file 1 [file Data_Sheet_1.ZIP › BAFF-R on memory B cell.leaveoneout.pdf]

# MR Method

- Inverse variance weighted
- MR Egger

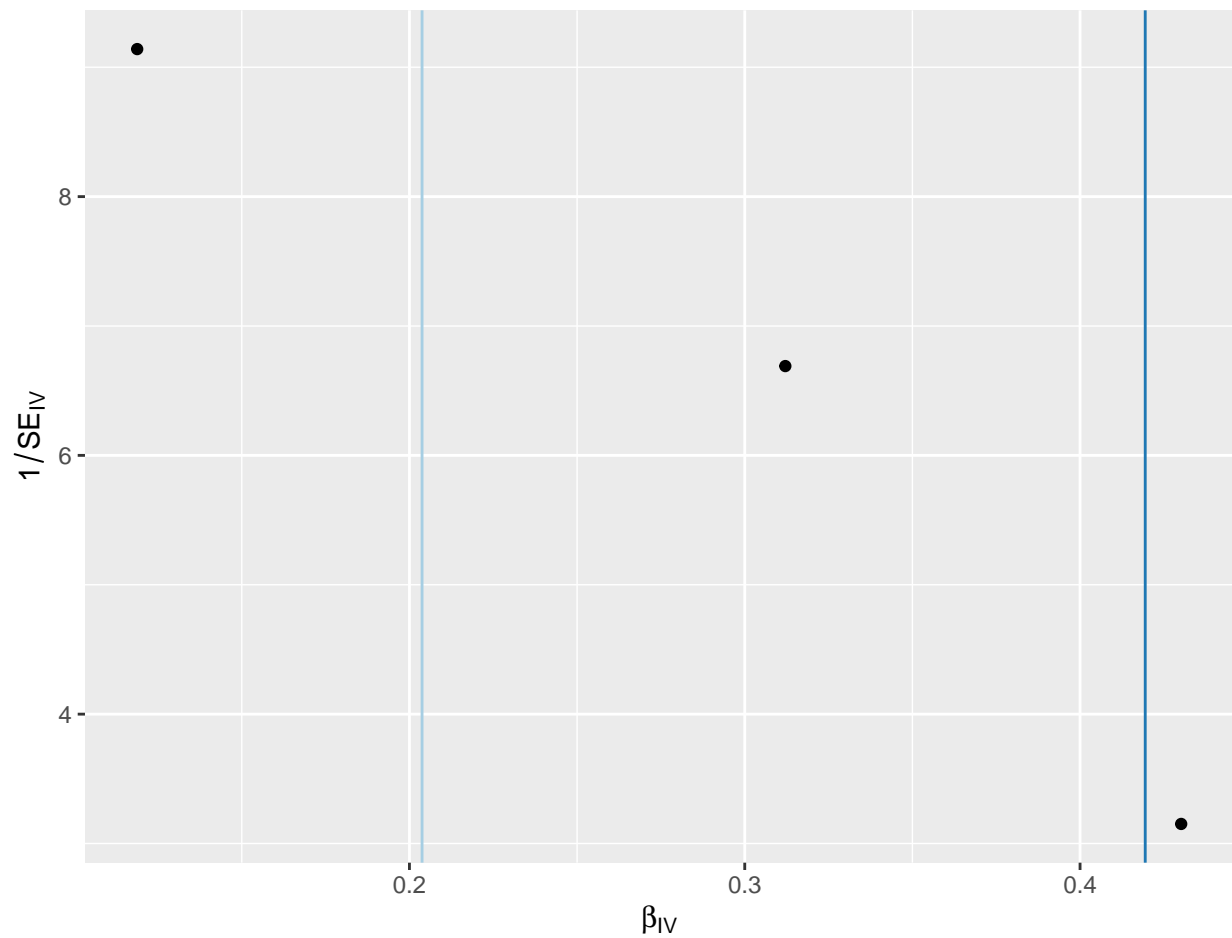

Supplement: Supplementary file 1 [file Data_Sheet_1.ZIP › BAFF-R on naive-mature B cell.funnel_plot.pdf]

# MR Method

- Inverse variance weighted
- MR Egger

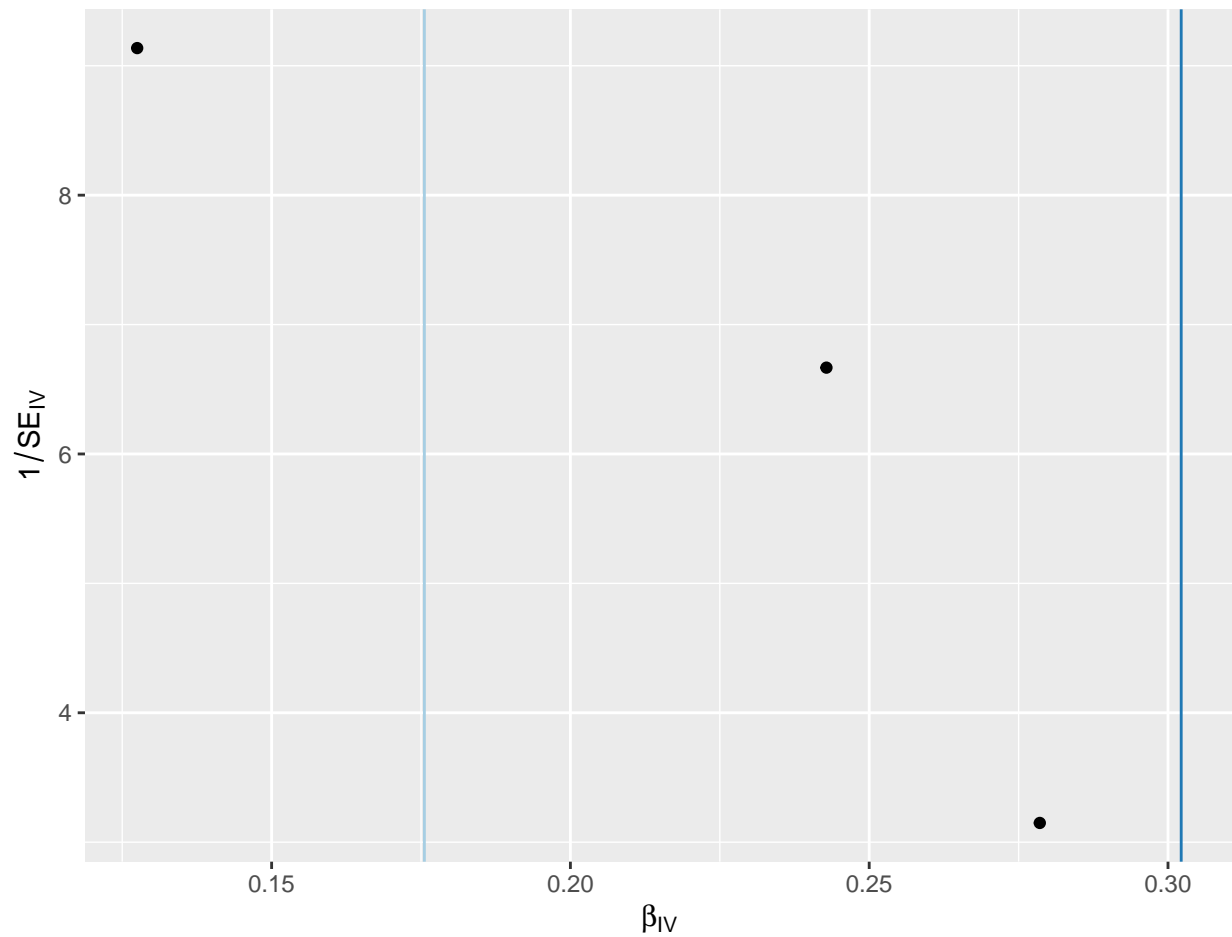

Supplement: Supplementary file 1 [file Data_Sheet_1.ZIP › BAFF-R on switched memory B cell.funnel_plot.pdf]

# MR Method

- Inverse variance weighted
- MR Egger

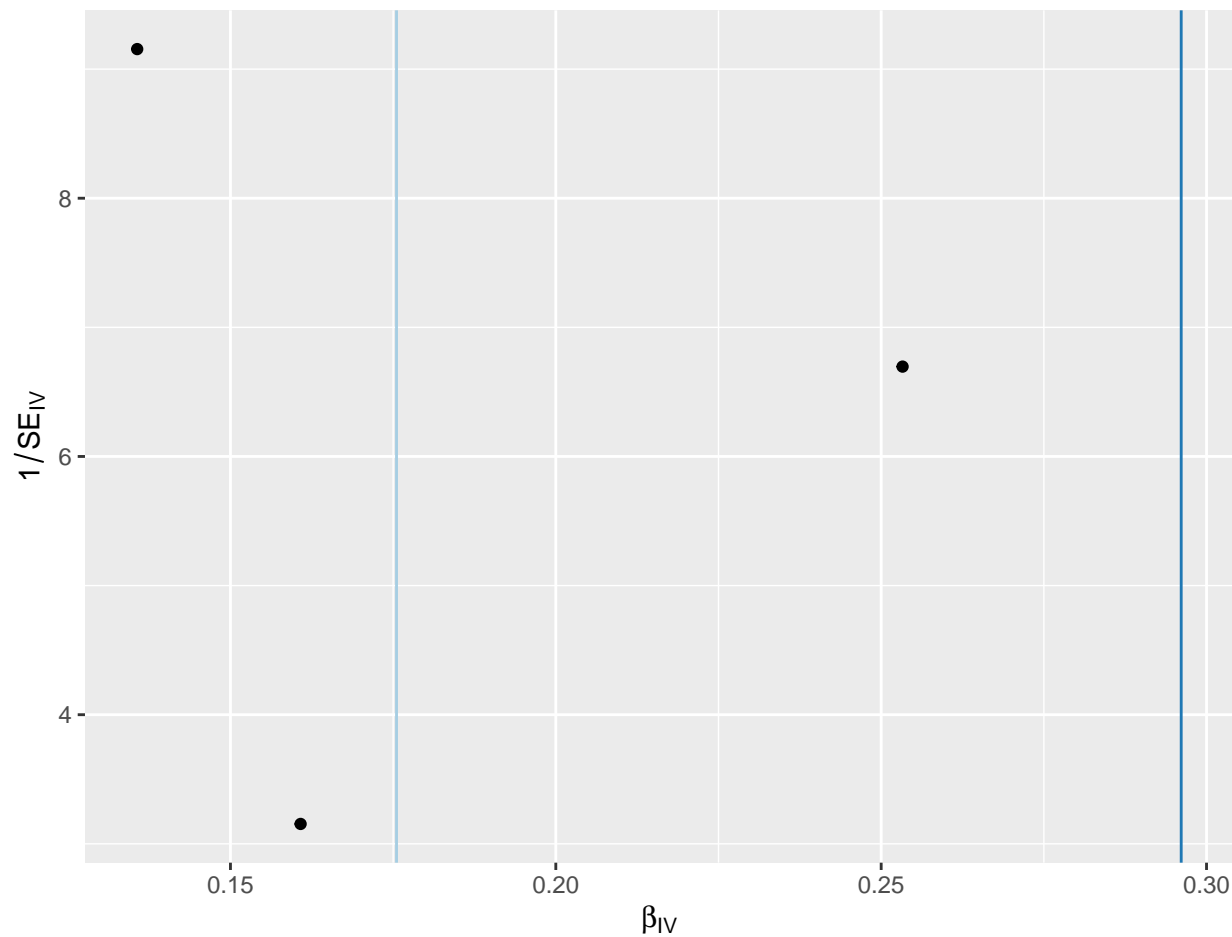

Supplement: Supplementary file 1 [file Data_Sheet_1.ZIP › BAFF-R on unswitched memory B cell.funnel_plot.pdf]

# MR Method

- Inverse variance weighted
- MR Egger

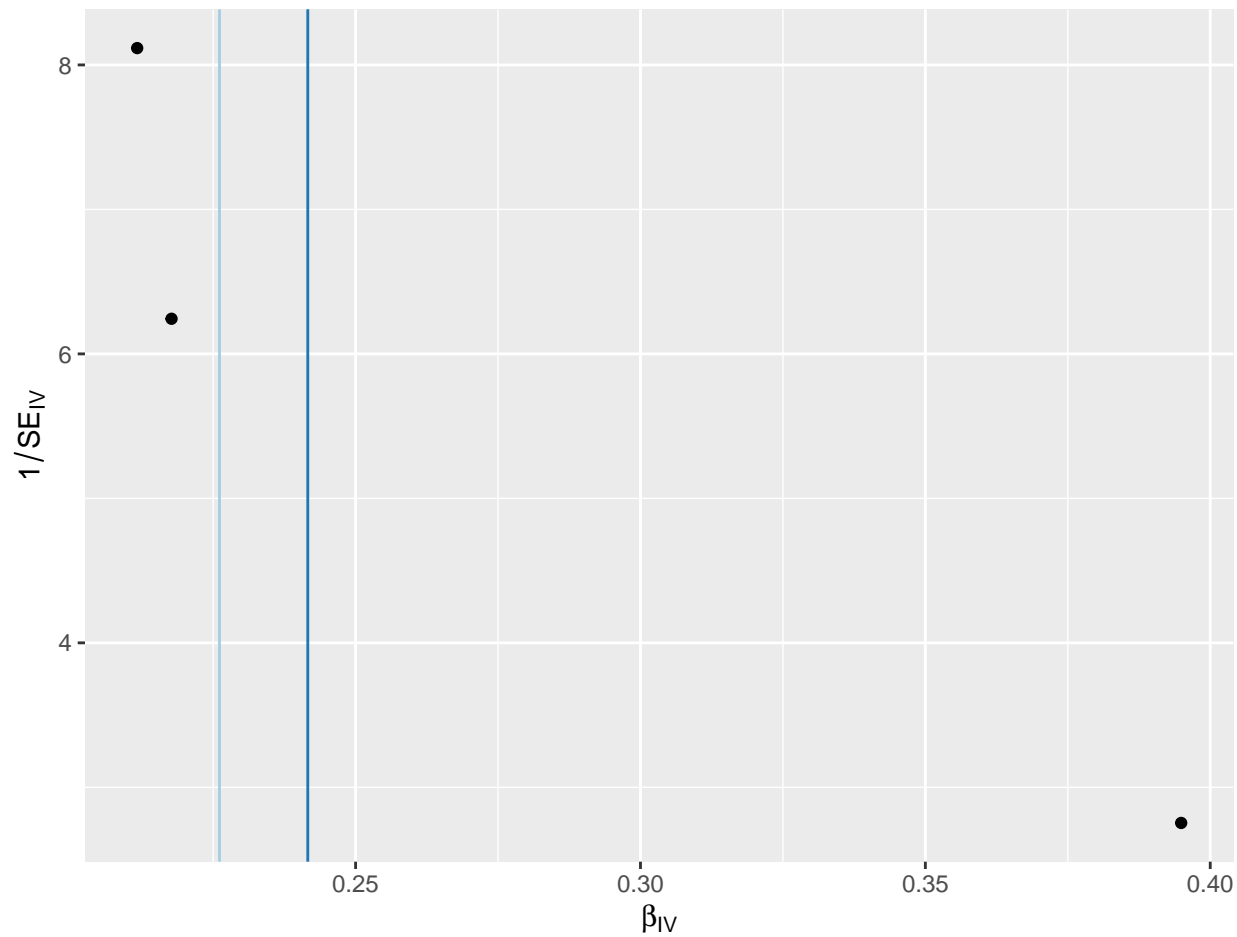

Supplement: Supplementary file 1 [file Data_Sheet_1.ZIP › CD123 on CD62L+ plasmacytoid Dendritic Cell.funnel_plot.pdf]

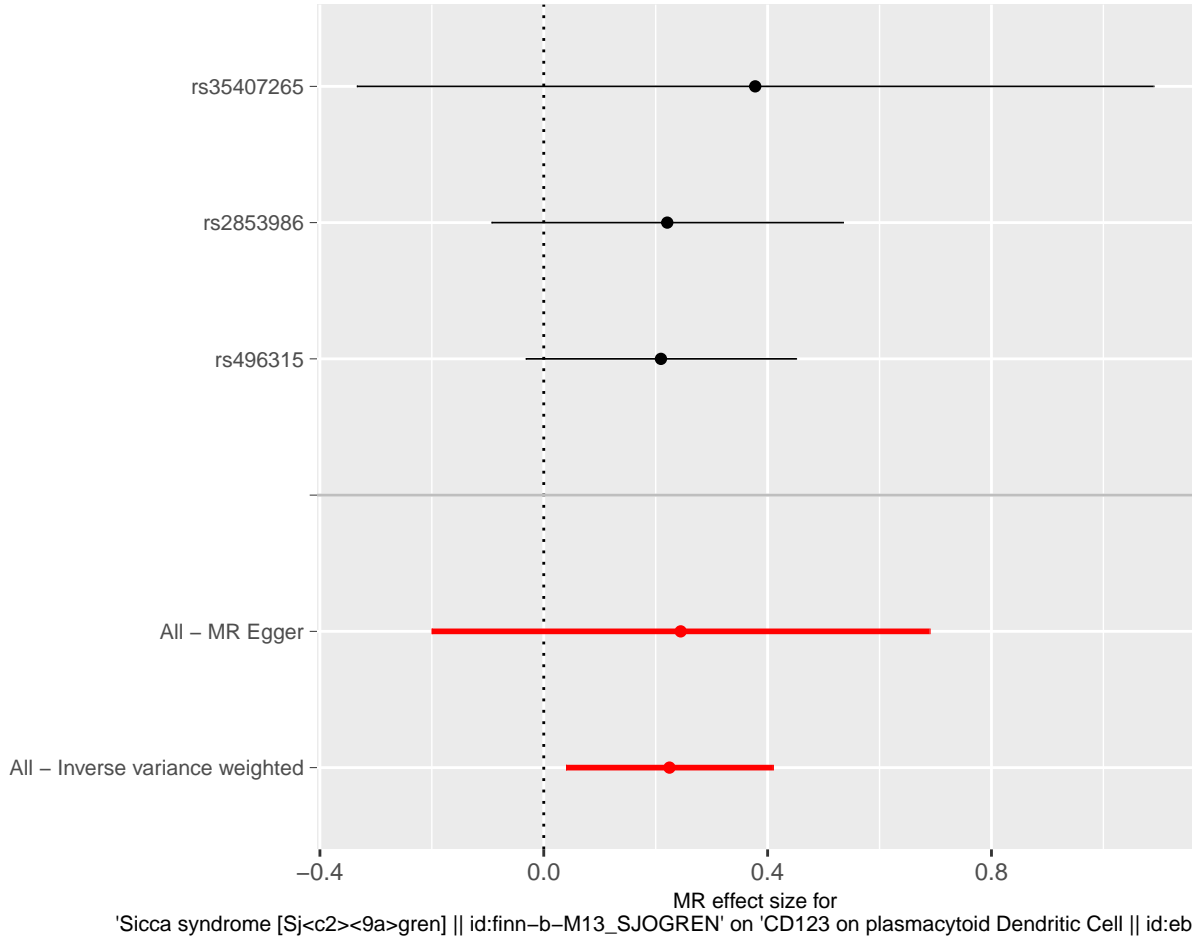

Supplement: Supplementary file 1 [file Data_Sheet_1.ZIP › CD123 on plasmacytoid Dendritic Cell.forest.pdf]

# MR Method

- Inverse variance weighted
- MR Egger

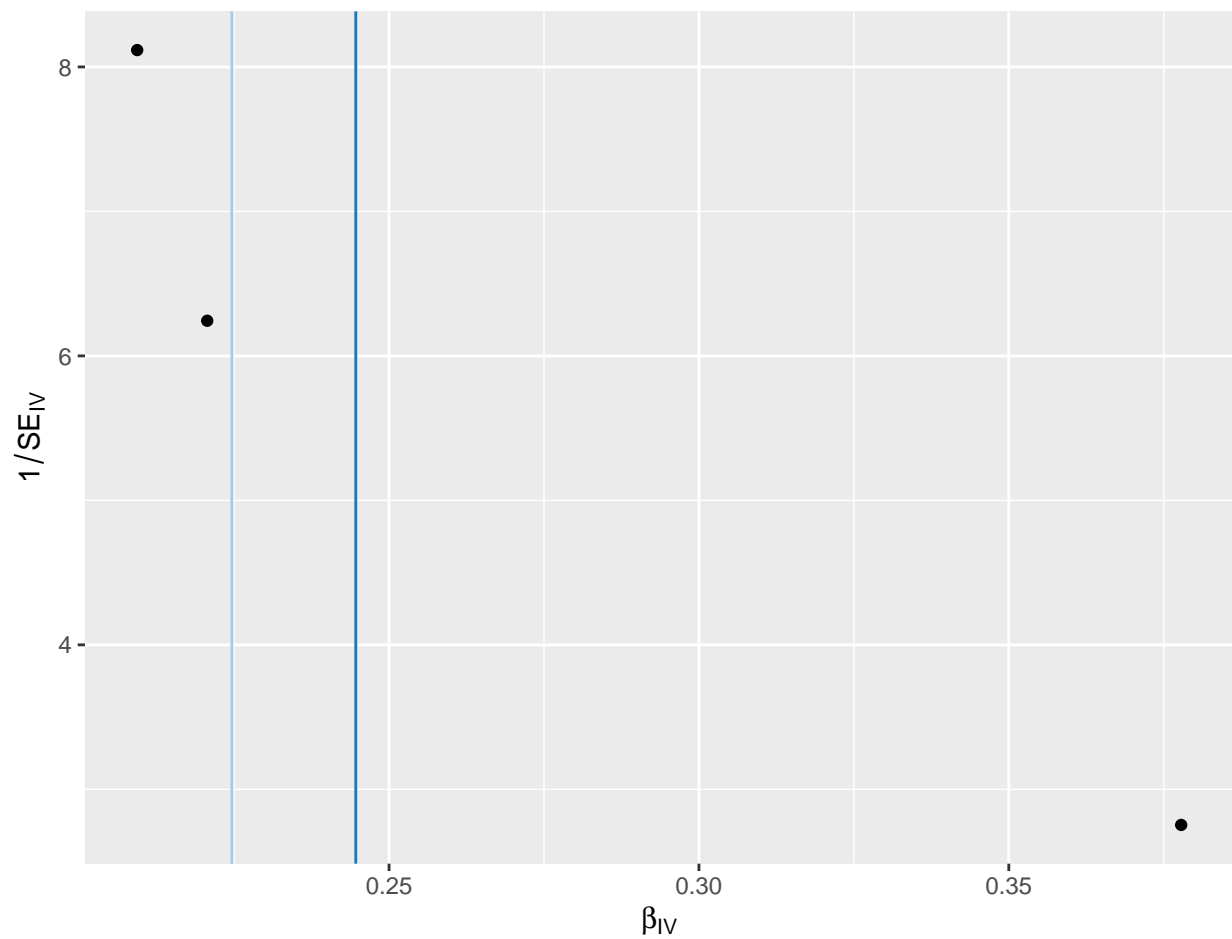

Supplement: Supplementary file 1 [file Data_Sheet_1.ZIP › CD123 on plasmacytoid Dendritic Cell.funnel_plot.pdf]

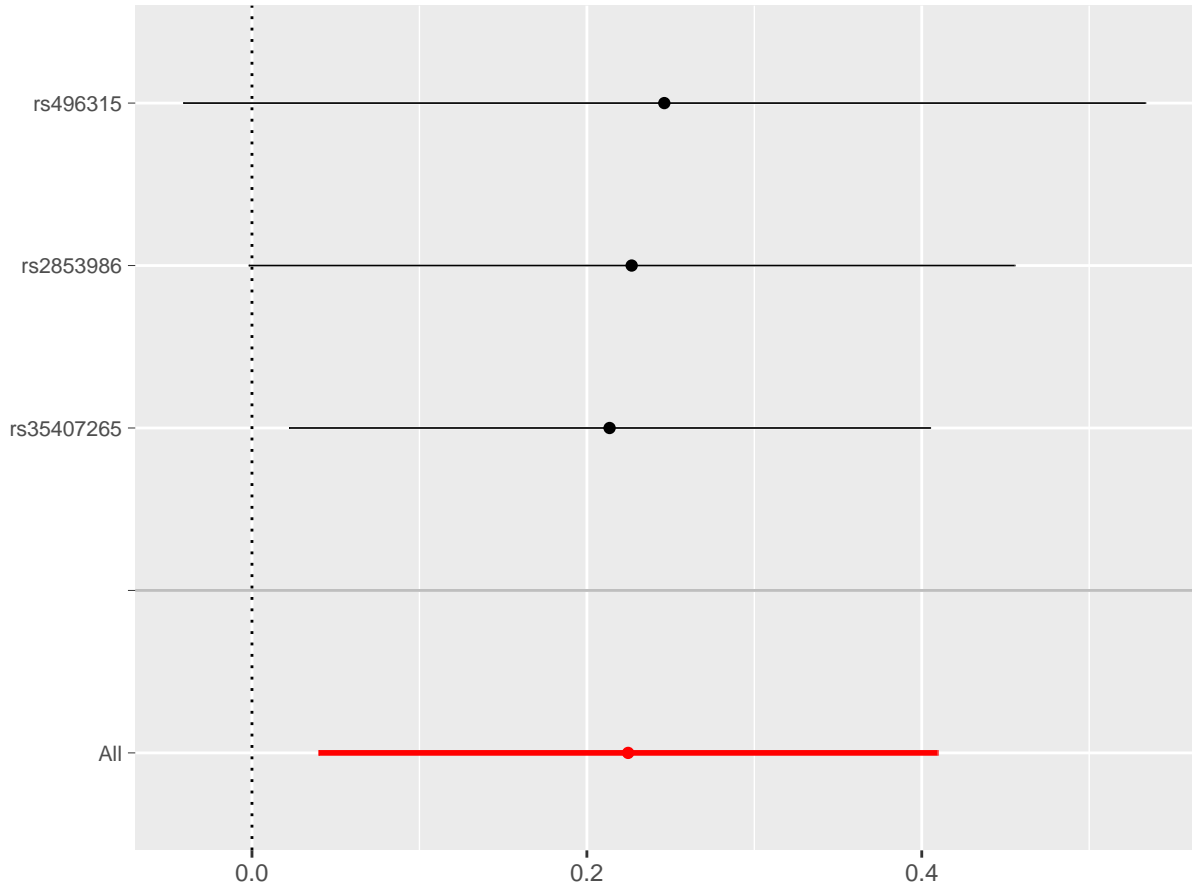

Supplement: Supplementary file 1 [file Data_Sheet_1.ZIP › CD123 on plasmacytoid Dendritic Cell.leaveoneout.pdf]

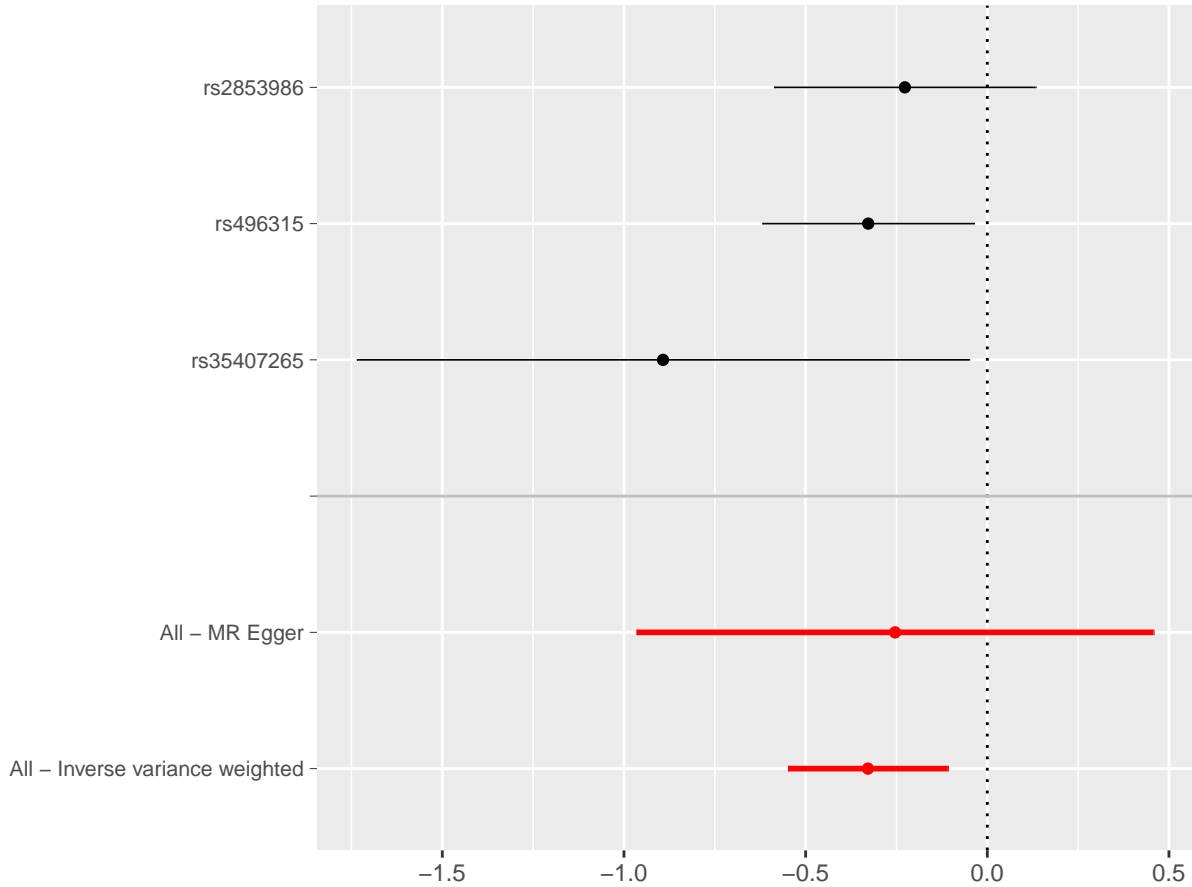

MR effect size for  
'Sicca syndrome [Sj<c2><9a>gren] || id:finn-b-M13\_SJOGREN' on 'CD25 on IgD+ CD38- naive B cell || id:ebi-a-

Supplement: Supplementary file 1 [file Data_Sheet_1.ZIP › CD25 on IgD+ CD38- naive B cell.forest.pdf]

# MR Method

- Inverse variance weighted
- MR Egger

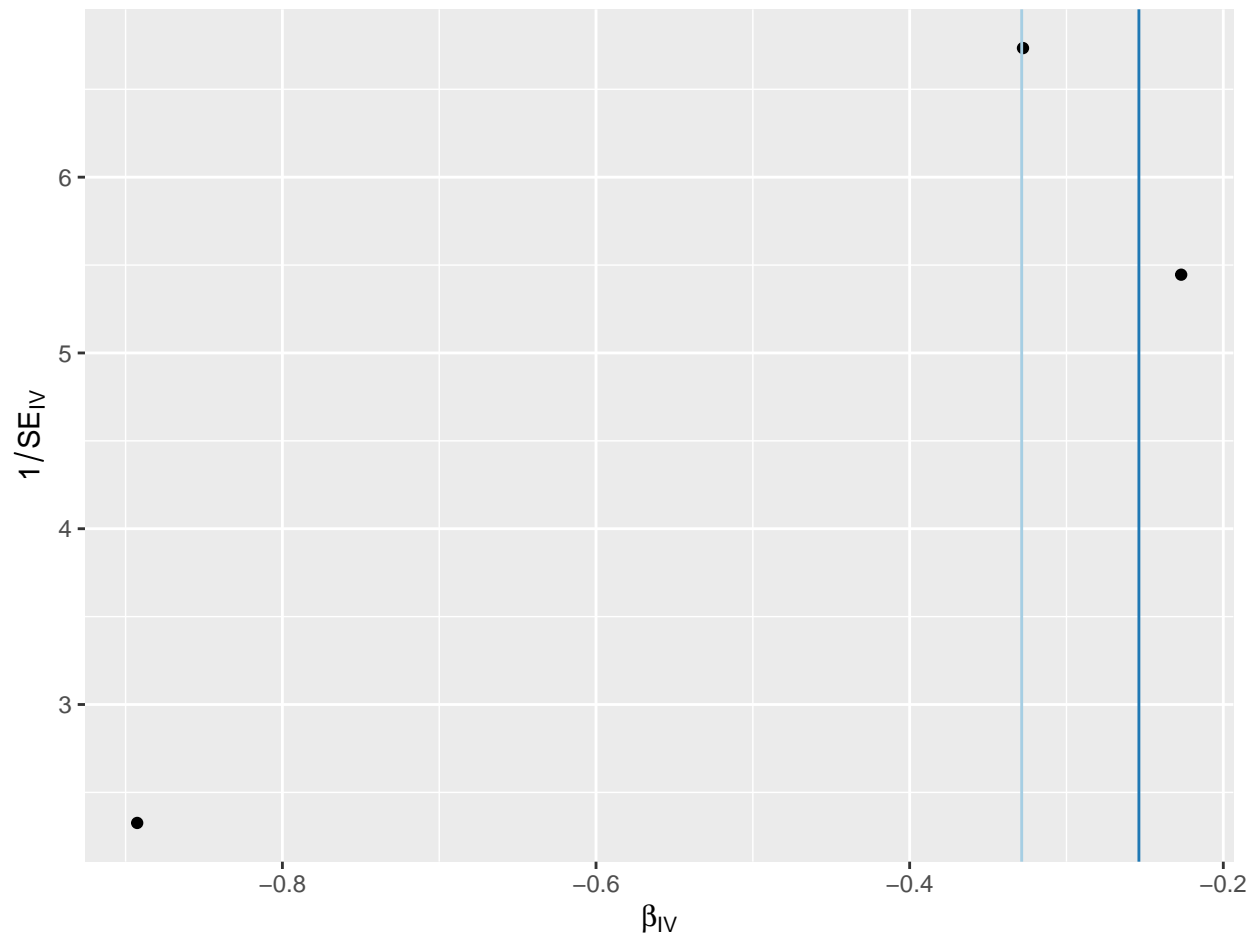

Supplement: Supplementary file 1 [file Data_Sheet_1.ZIP › CD25 on IgD+ CD38- naive B cell.funnel_plot.pdf]

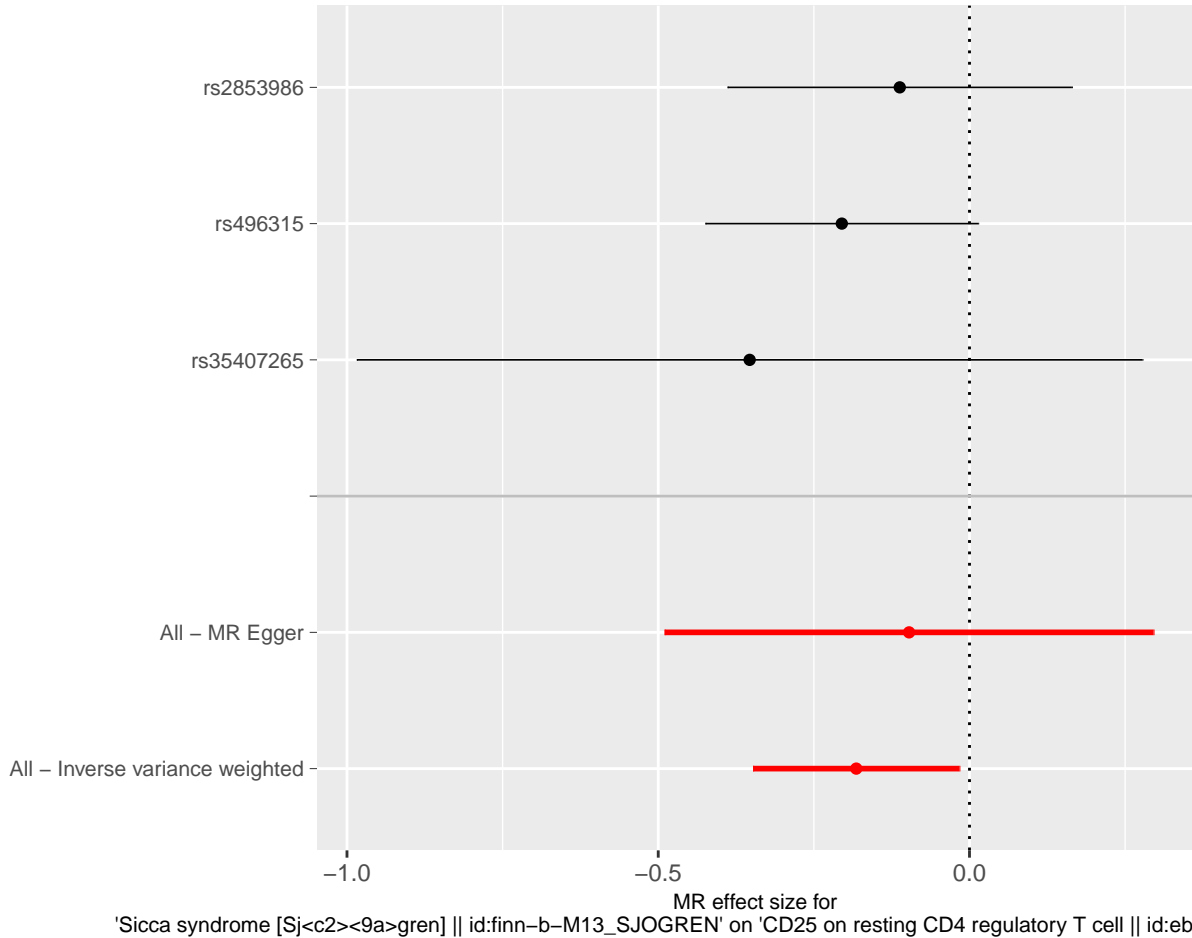

Supplement: Supplementary file 1 [file Data_Sheet_1.ZIP › CD25 on resting CD4 regulatory T cell.forest.pdf]

# MR Method

- Inverse variance weighted
- MR Egger

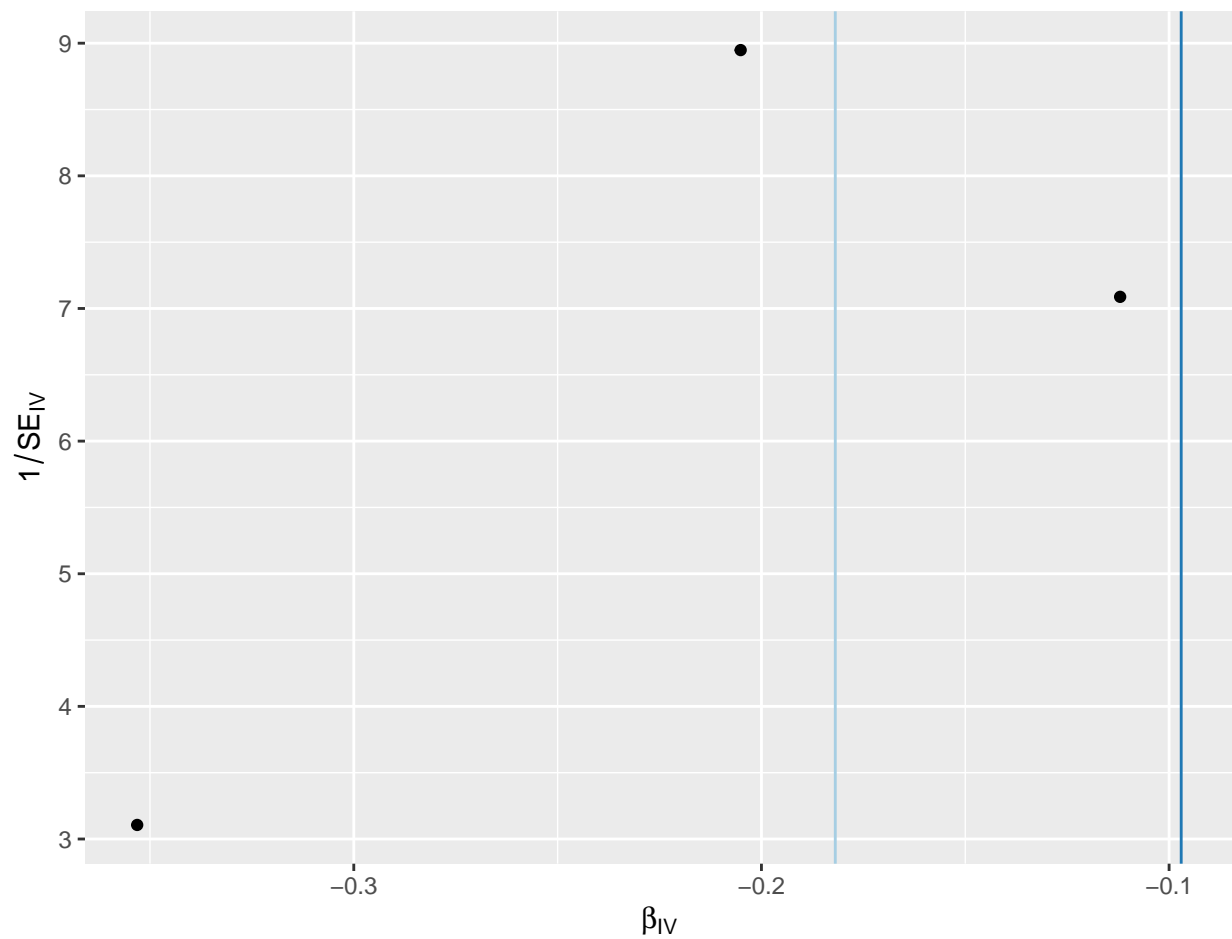

Supplement: Supplementary file 1 [file Data_Sheet_1.ZIP › CD25 on resting CD4 regulatory T cell.funnel_plot.pdf]

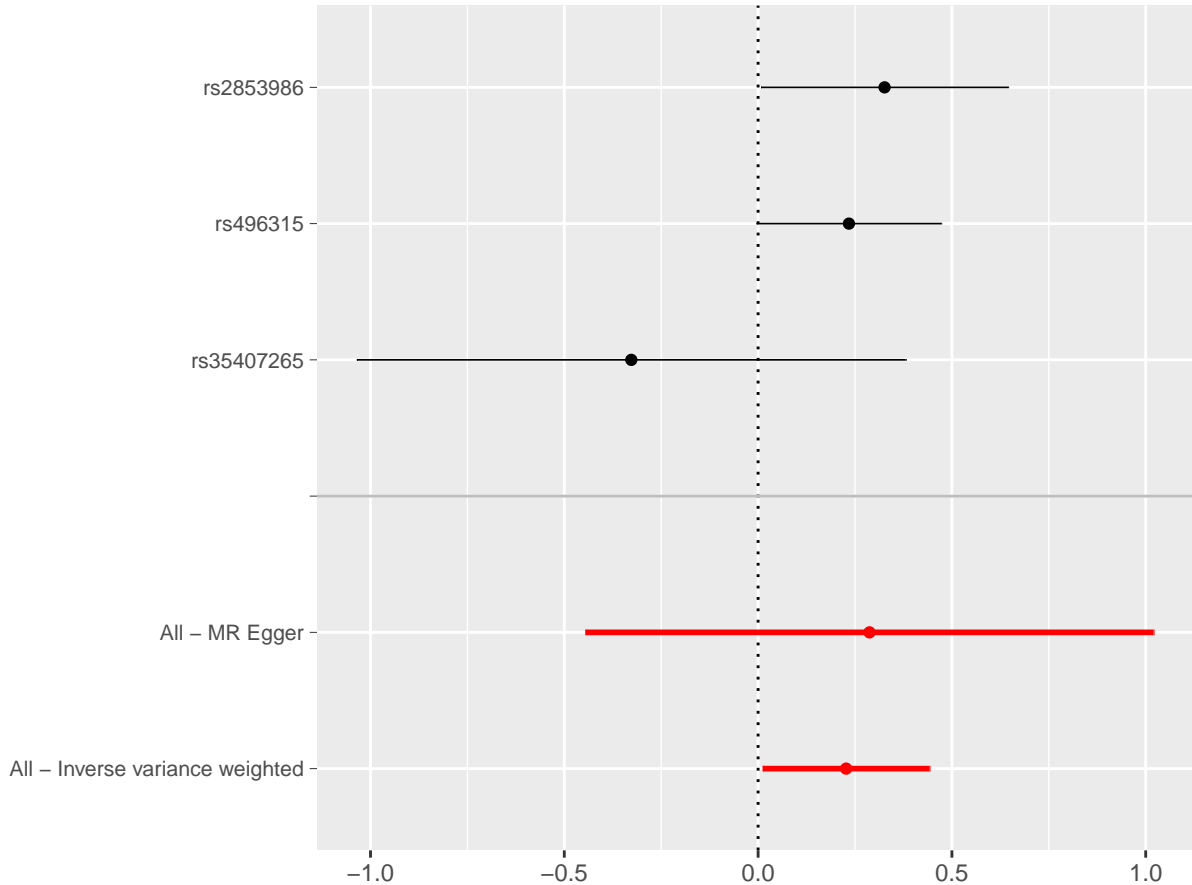

MR effect size for  
'Sicca syndrome [Sj<c2><9a>gren] || id:finn-b-M13\_SJOGREN' on 'CD39 on CD39+ CD4+ T cell || id:ebi-a-G

Supplement: Supplementary file 1 [file Data_Sheet_1.ZIP › CD39 on CD39+ CD4+ T cell.forest.pdf]

# MR Method

- Inverse variance weighted
- MR Egger

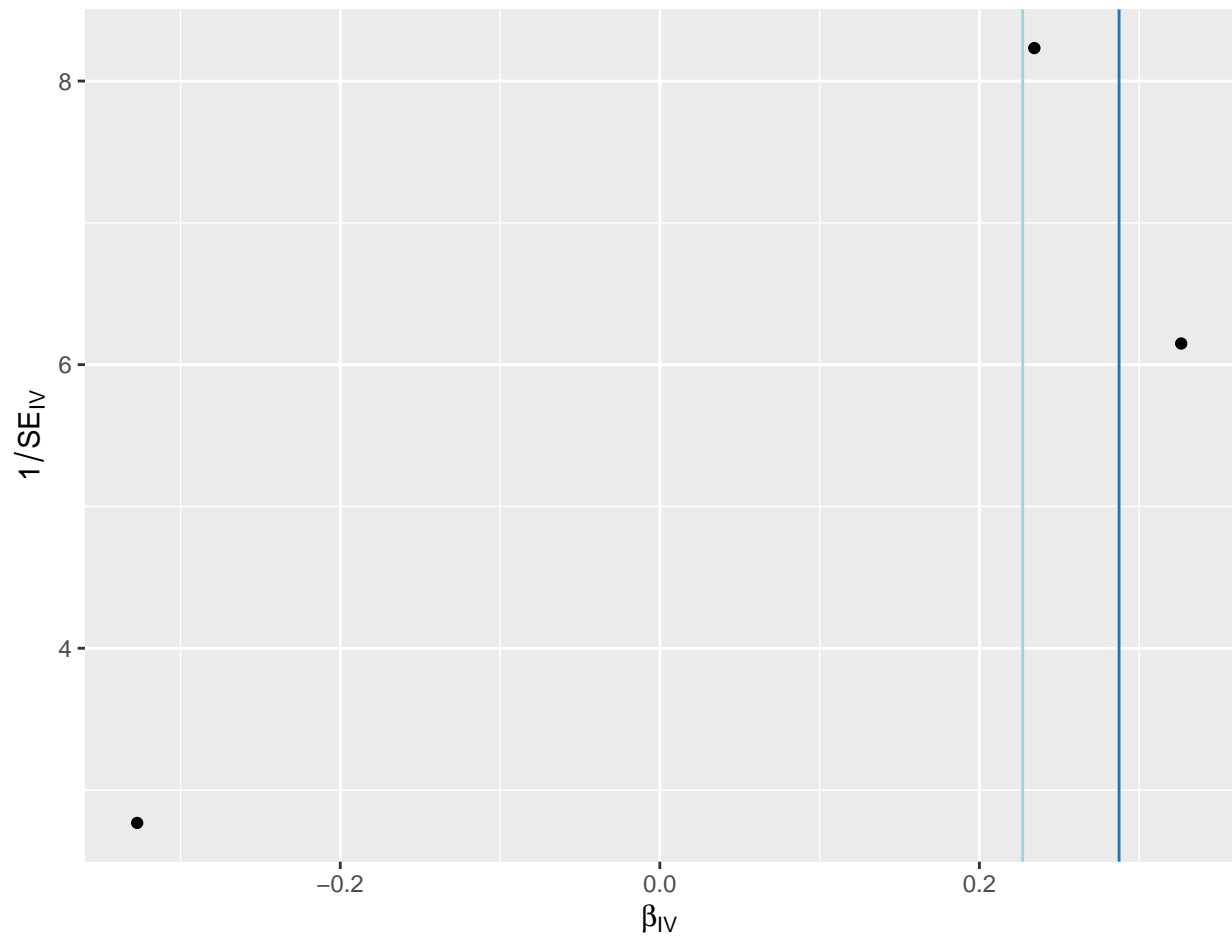

Supplement: Supplementary file 1 [file Data_Sheet_1.ZIP › CD39 on CD39+ CD4+ T cell.funnel_plot.pdf]

# MR Test

- Inverse variance weighted
- MR Egger
- Simple mode
- Weighted median
- Weighted mode

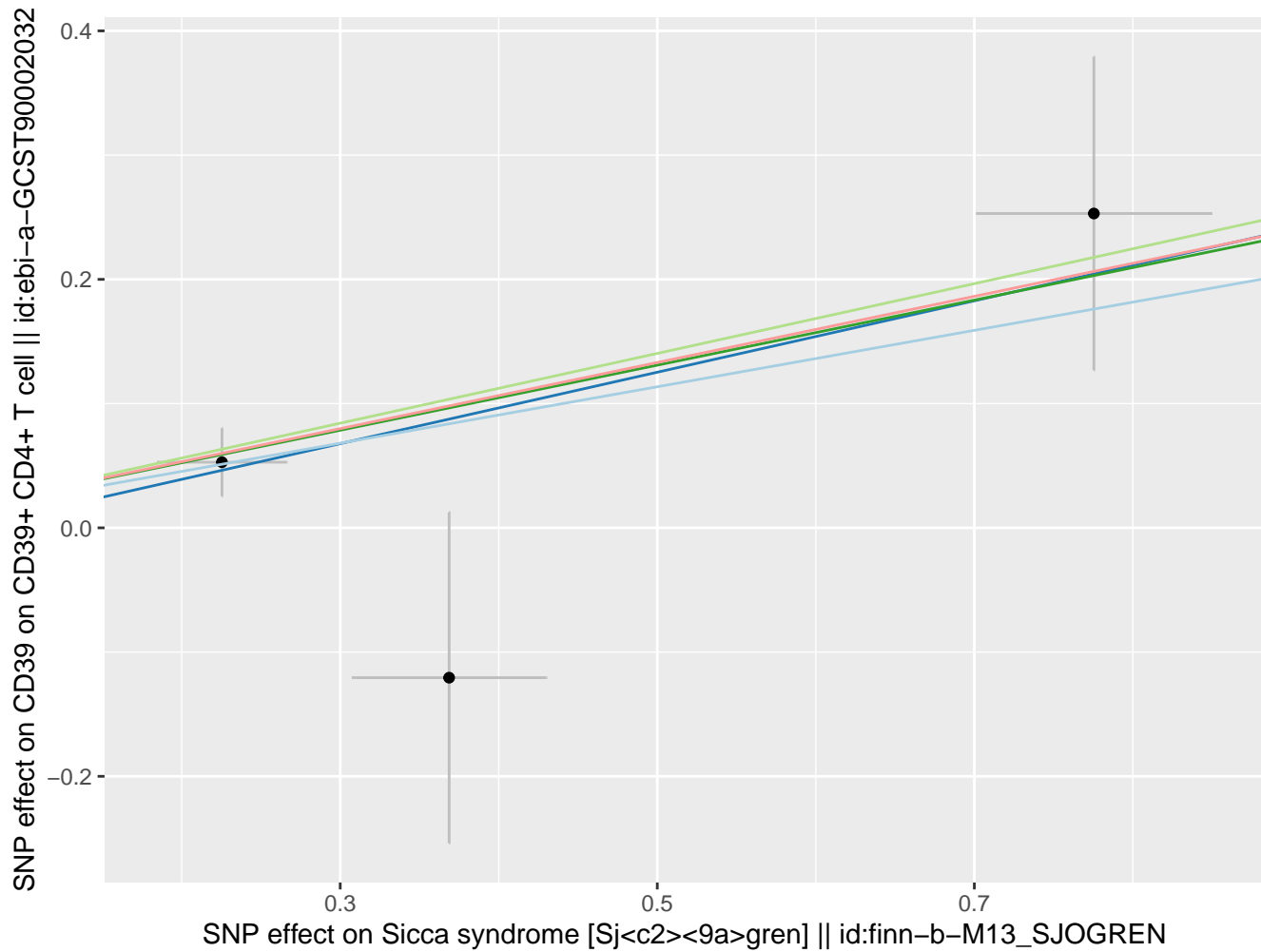

Supplement: Supplementary file 1 [file Data_Sheet_1.ZIP › CD39 on CD39+ CD4+ T cell.scatter_plot.pdf]

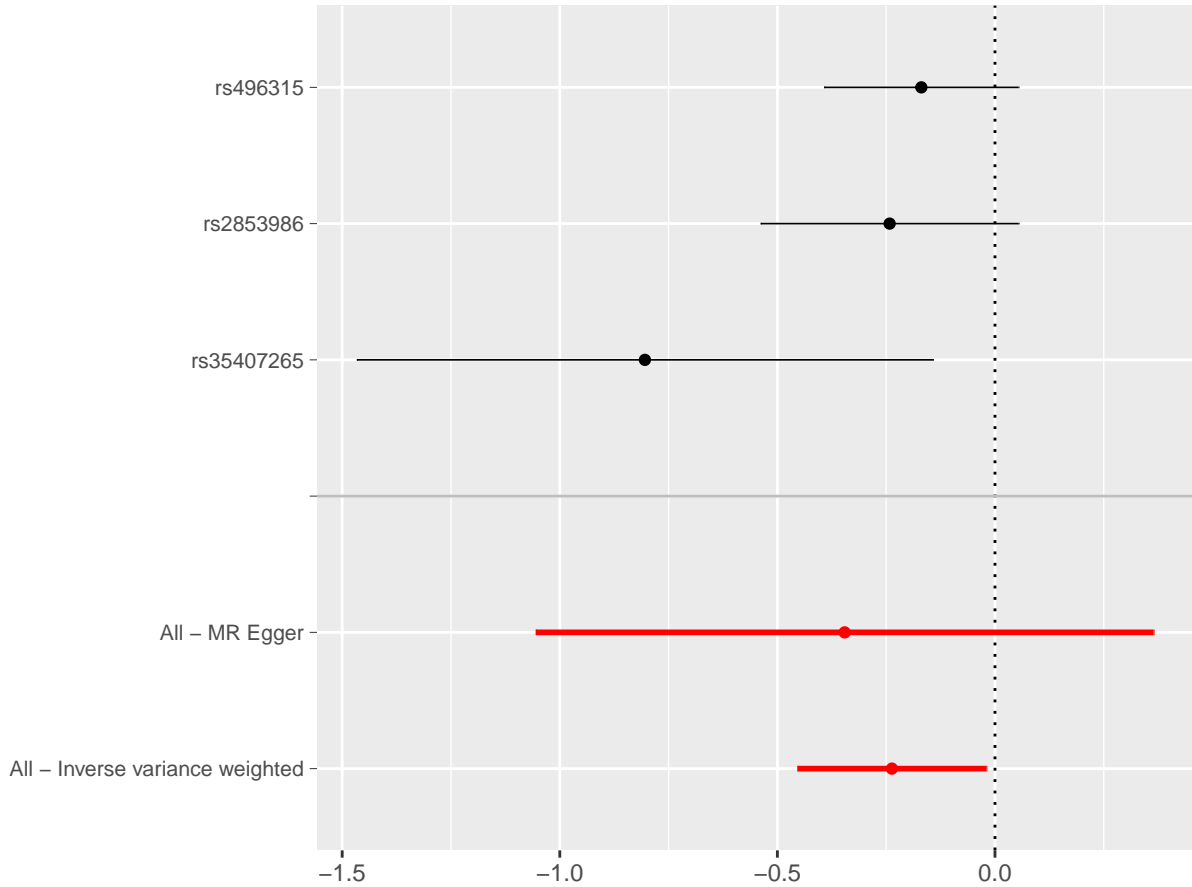

MR effect size for  
'Sicca syndrome [Sj<c2><9a>gren] || id:finn-b-M13\_SJOGREN' on 'CD45RA on naive CD4+ T cell || id:ebi-a-G

Supplement: Supplementary file 1 [file Data_Sheet_1.ZIP › CD45RA on naive CD4+ T cell.forest.pdf]

# MR Method

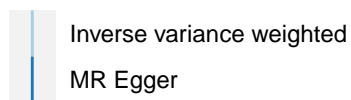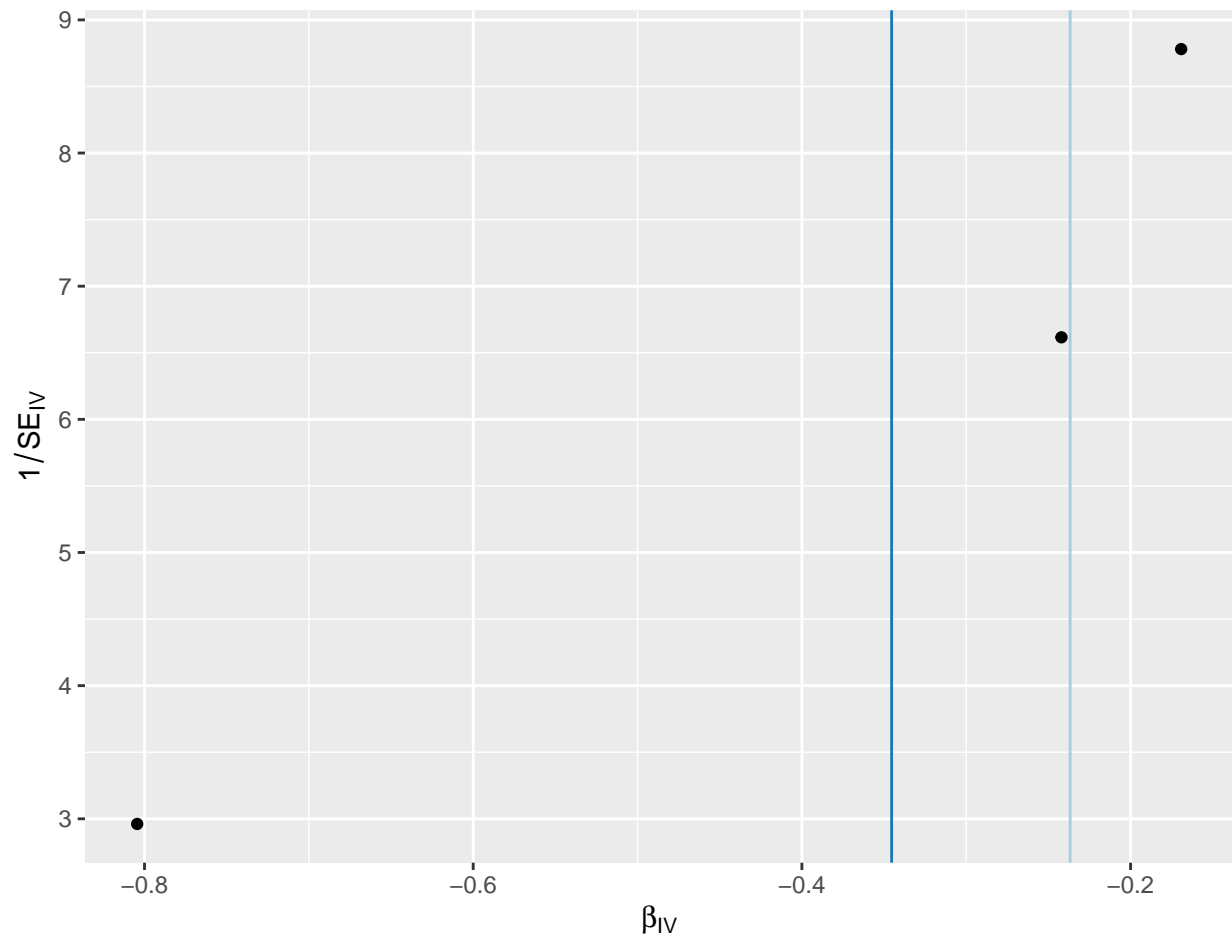

Supplement: Supplementary file 1 [file Data_Sheet_1.ZIP › CD45RA on naive CD4+ T cell.funnel_plot.pdf]

# MR Method

- Inverse variance weighted
- MR Egger

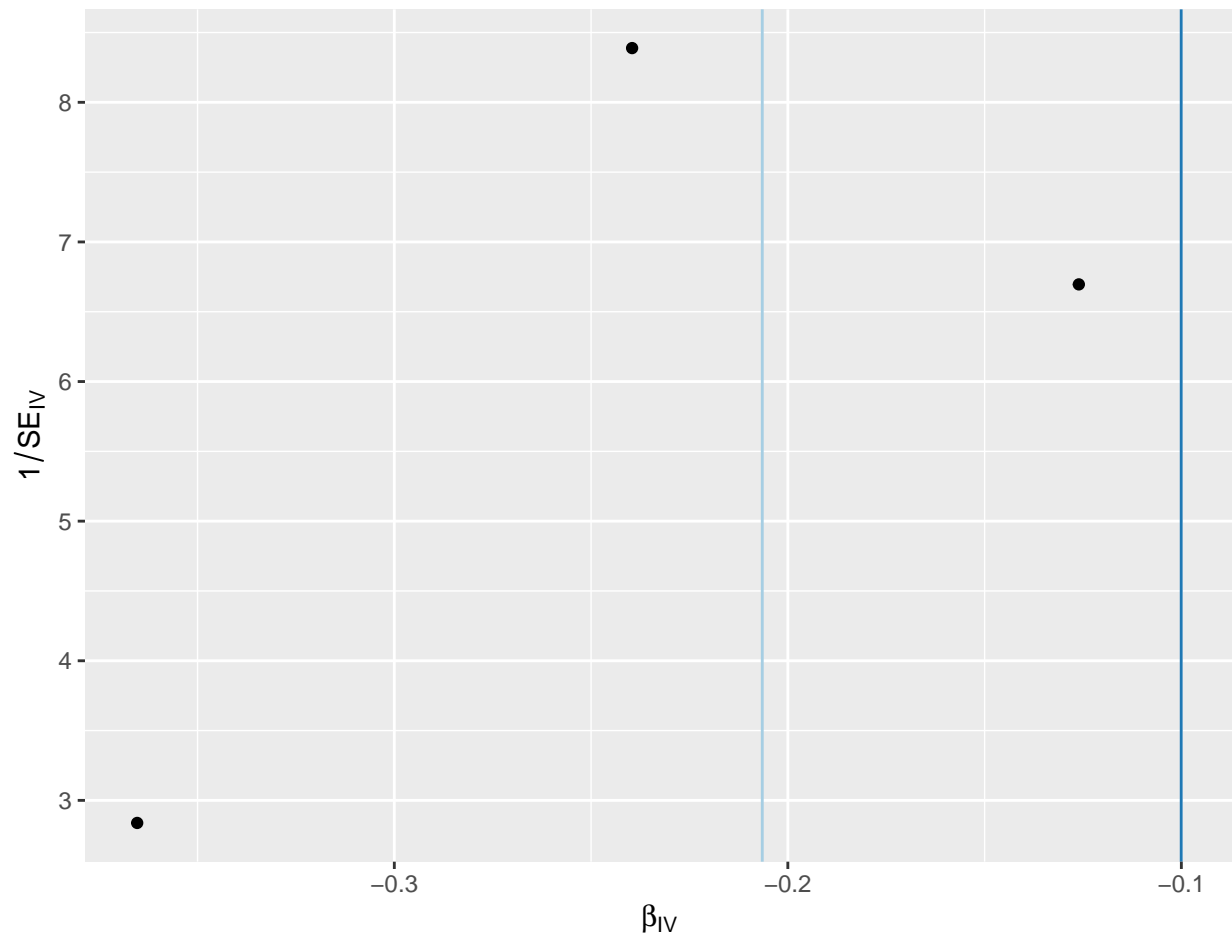

Supplement: Supplementary file 1 [file Data_Sheet_1.ZIP › CD4RA on Terminally Differentiated CD4+ T cell.funnel_plot.pdf]

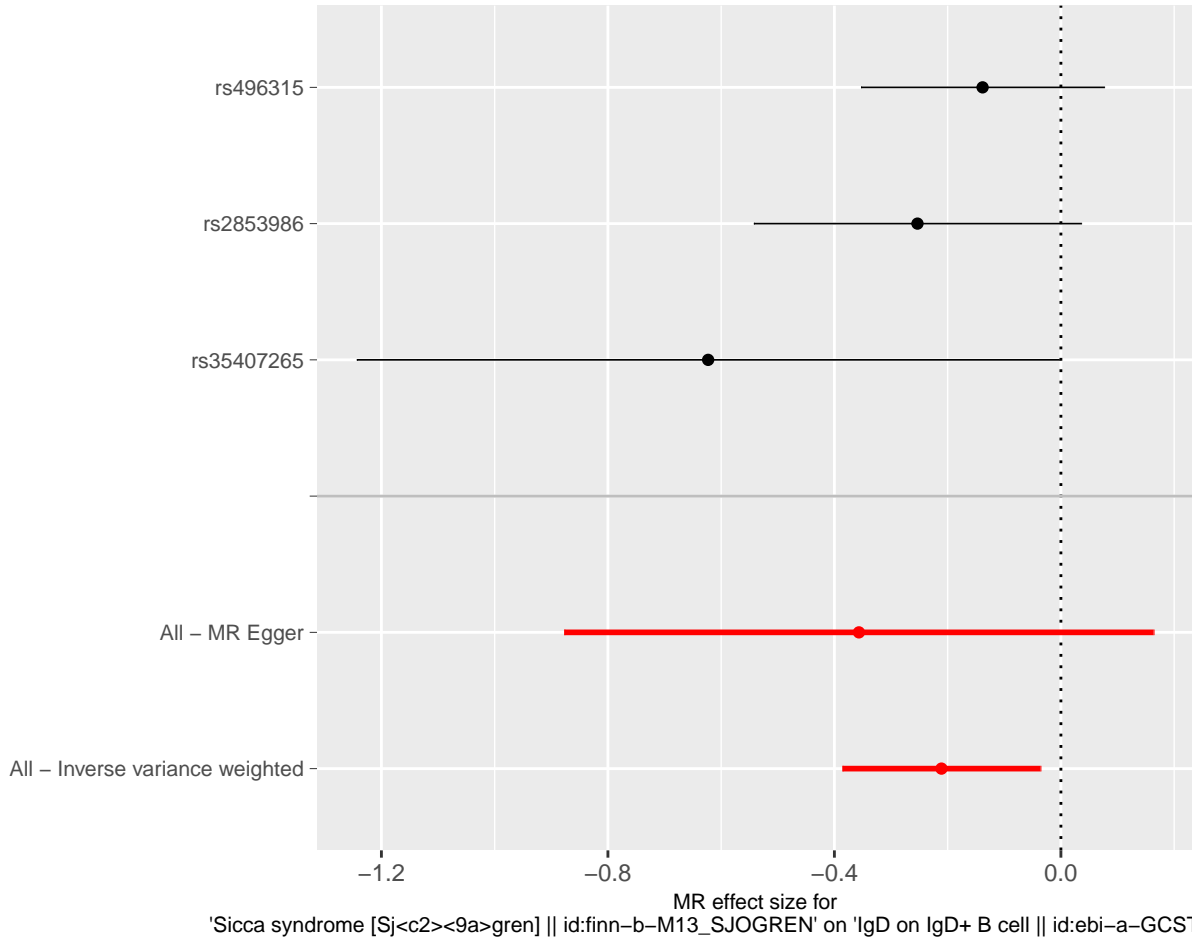

Supplement: Supplementary file 1 [file Data_Sheet_1.ZIP › IgD on IgD+ B cell.forest.pdf]

# MR Method

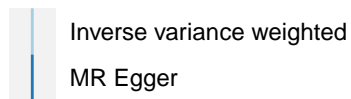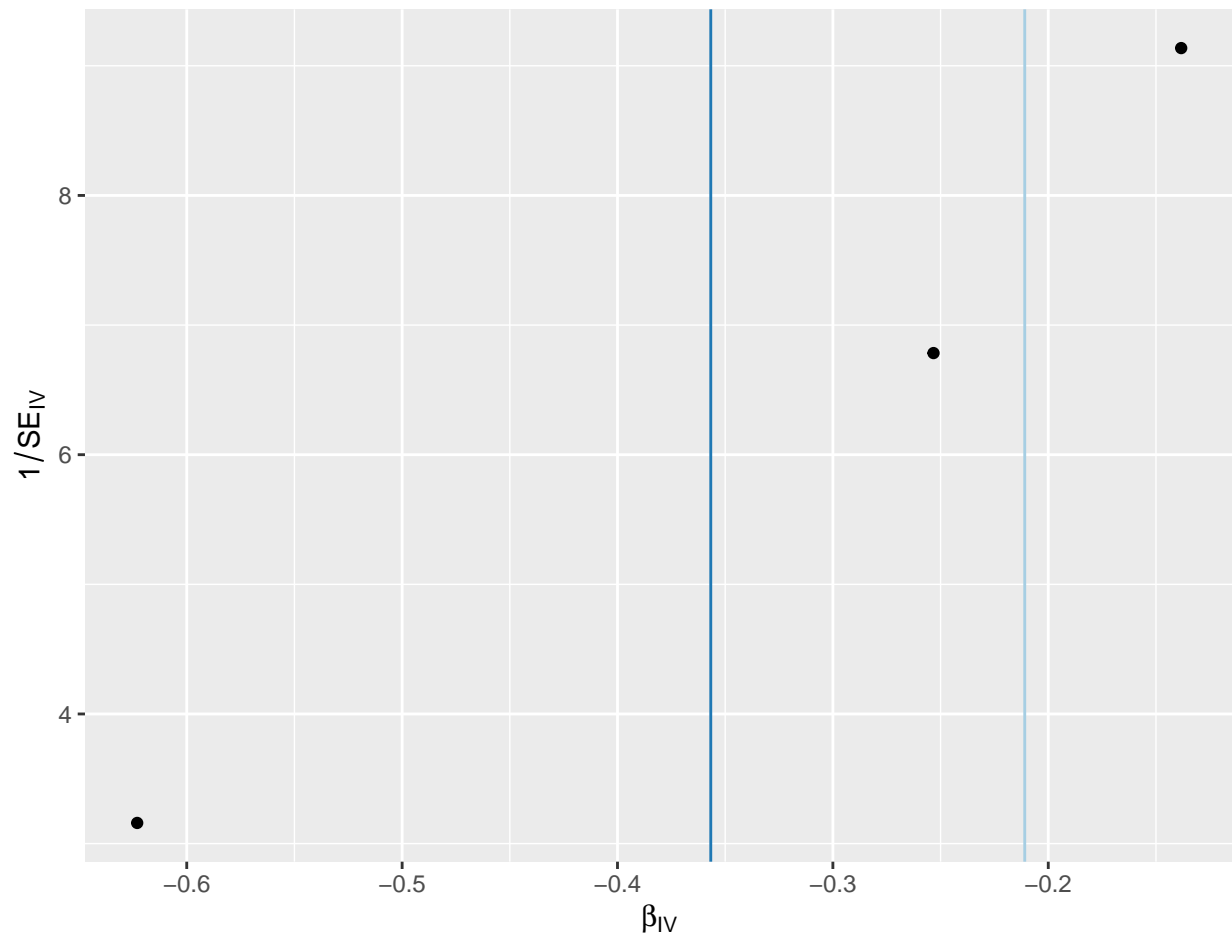

Supplement: Supplementary file 1 [file Data_Sheet_1.ZIP › IgD on IgD+ B cell.funnel_plot.pdf]

# MR Test

- Inverse variance weighted
- MR Egger
- Simple mode
- Weighted median
- Weighted mode

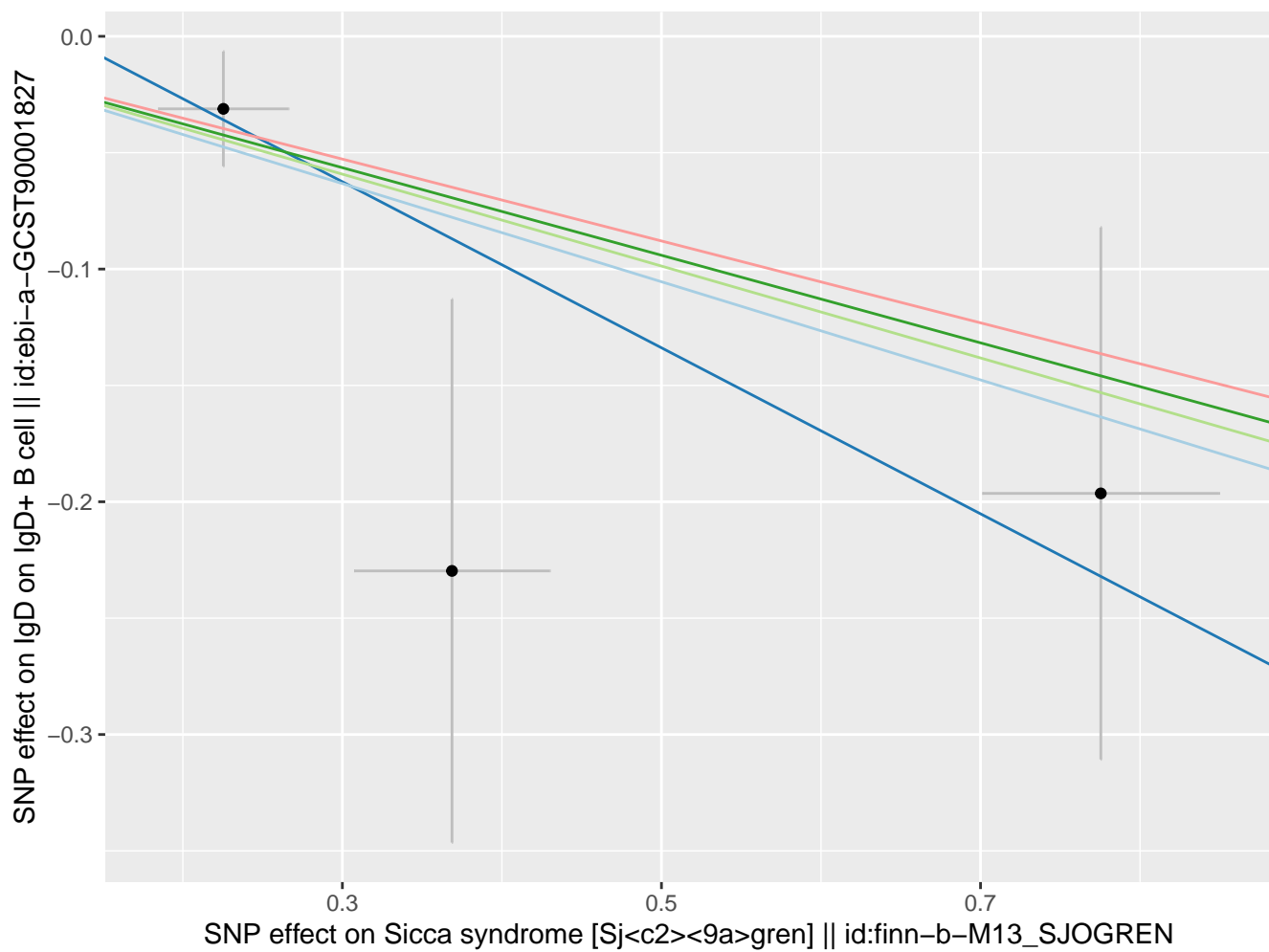

Supplement: Supplementary file 1 [file Data_Sheet_1.ZIP › IgD on IgD+ B cell.scatter_plot.pdf]

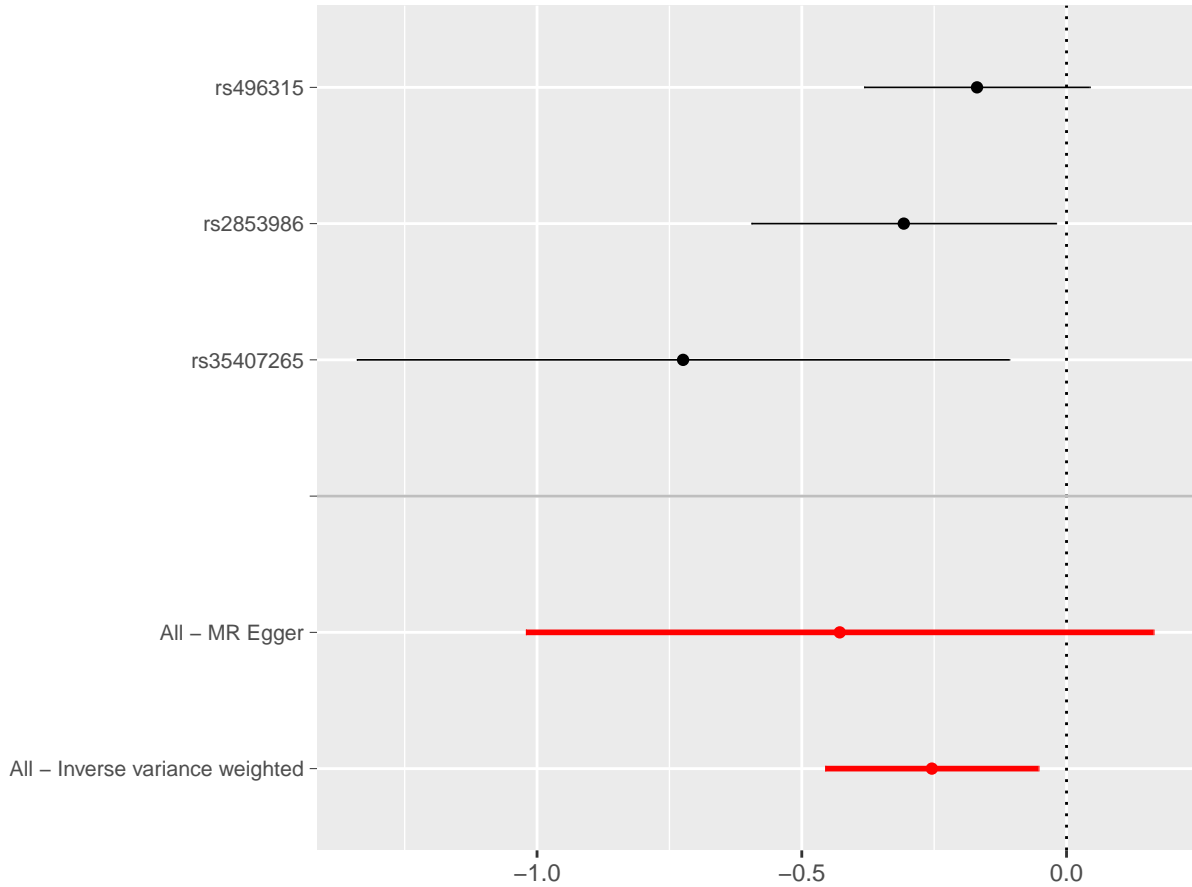

MR effect size for  
'Sicca syndrome [Sj<c2><9a>gren] || id:finn-b-M13\_SJOGREN' on 'IgD on IgD+ CD24- B cell || id:ebi-a-GC

Supplement: Supplementary file 1 [file Data_Sheet_1.ZIP › IgD on IgD+ CD24- B cell.forest.pdf]

# MR Method

- Inverse variance weighted
- MR Egger

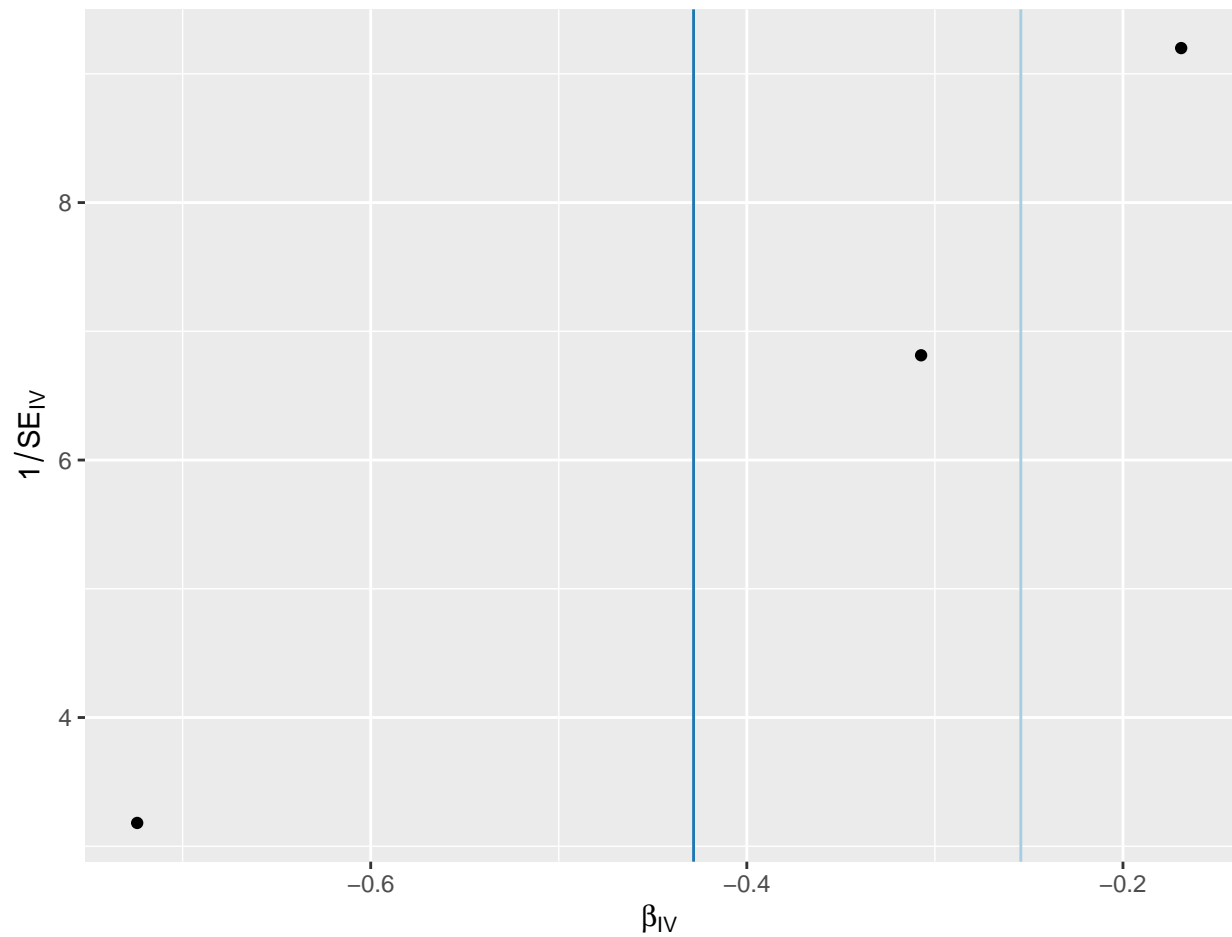

Supplement: Supplementary file 1 [file Data_Sheet_1.ZIP › IgD on IgD+ CD24- B cell.funnel_plot.pdf]

# MR Test

- Inverse variance weighted
- MR Egger
- Simple mode
- Weighted median
- Weighted mode

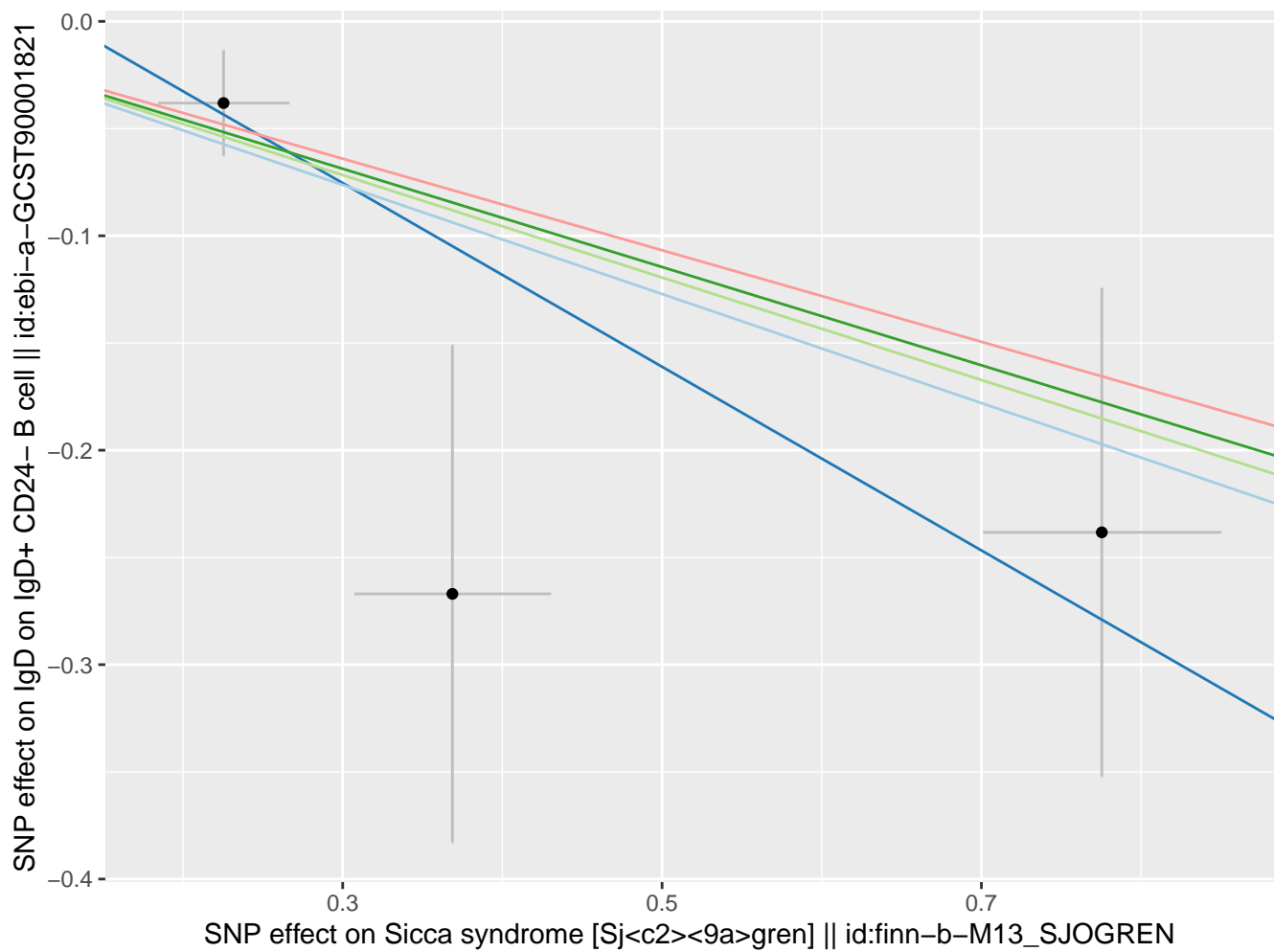

Supplement: Supplementary file 1 [file Data_Sheet_1.ZIP › IgD on IgD+ CD24- B cell.scatter_plot.pdf]

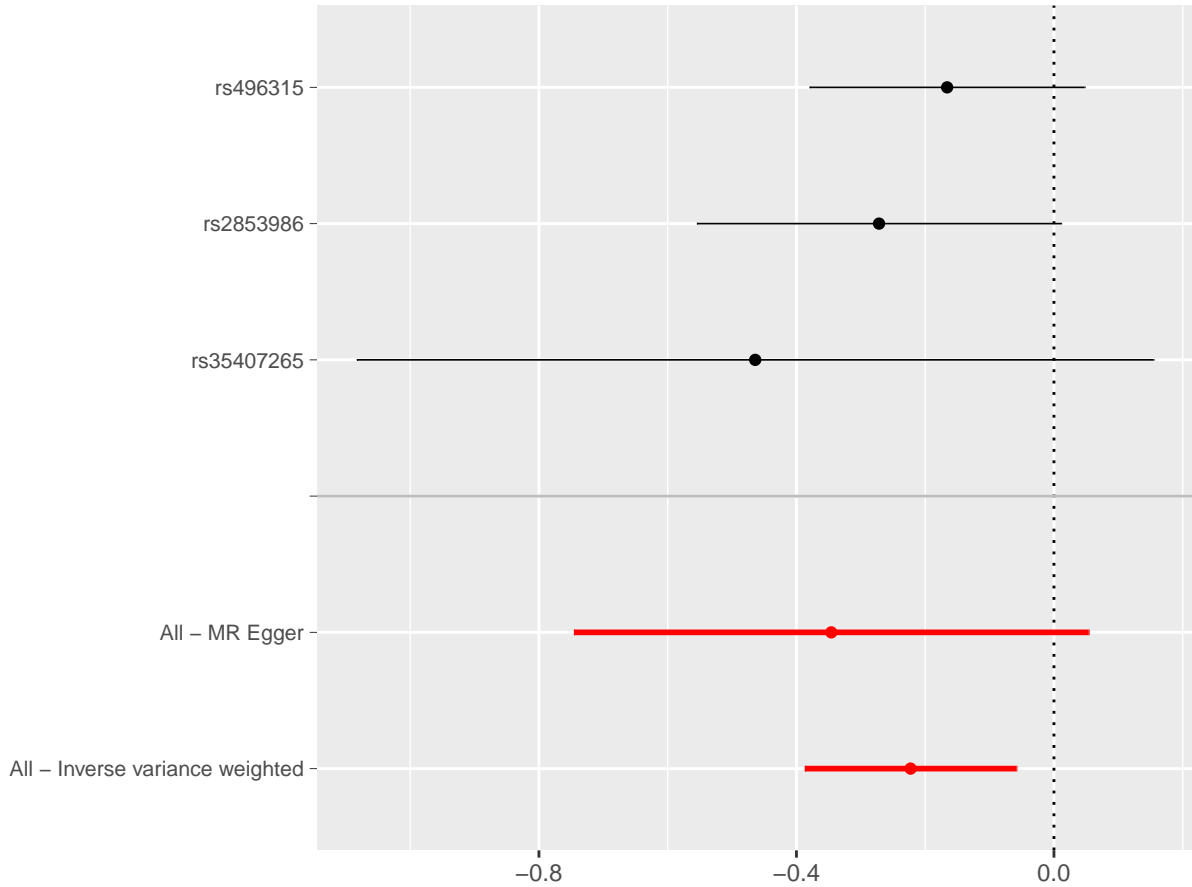

MR effect size for  
'Sicca syndrome [Sj<c2><9a>gren] || id:finn-b-M13\_SJOGREN' on 'IgD on IgD+ CD38- B cell || id:ebi-a-GC

Supplement: Supplementary file 1 [file Data_Sheet_1.ZIP › IgD on IgD+ CD38- B cell.forest.pdf]

# MR Method

- Inverse variance weighted
- MR Egger

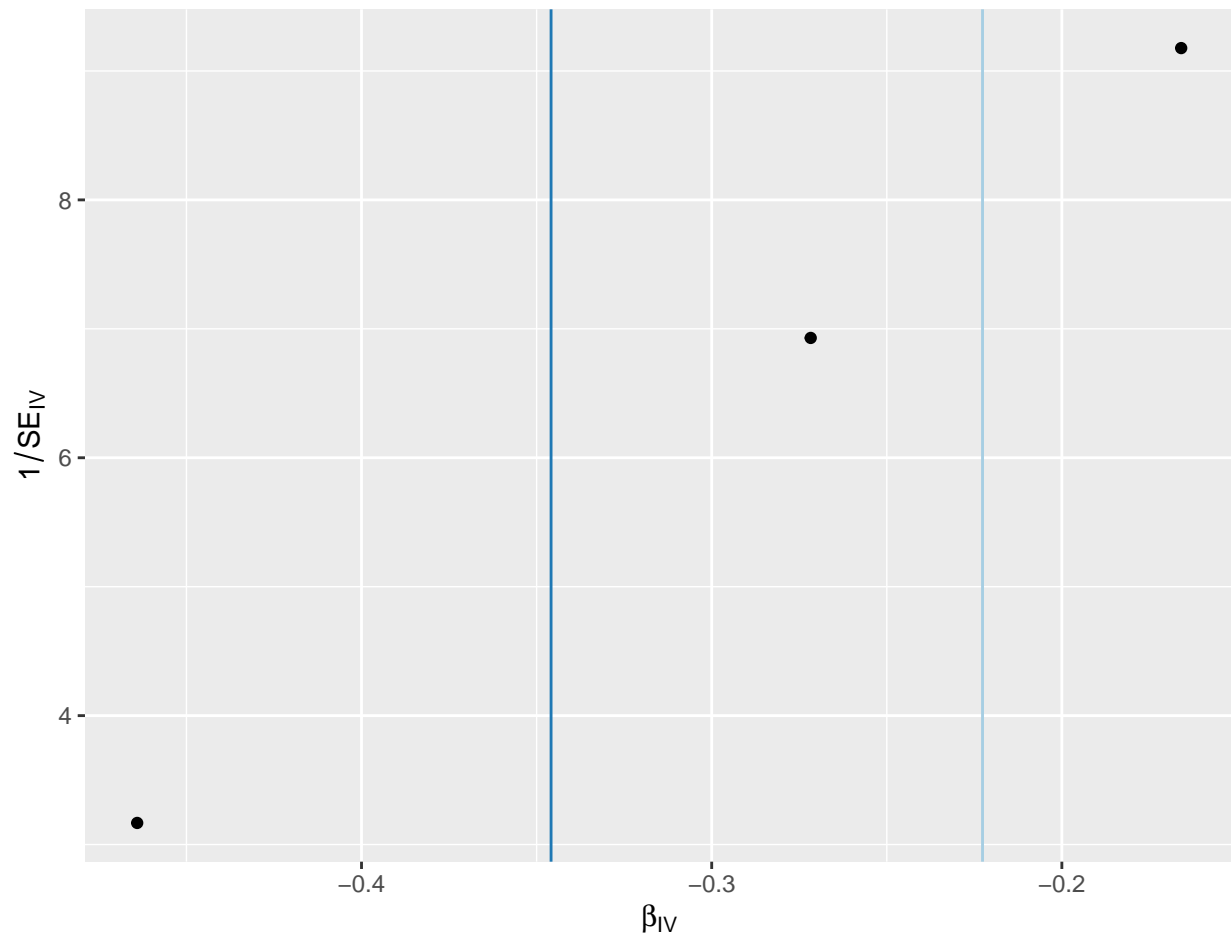

Supplement: Supplementary file 1 [file Data_Sheet_1.ZIP › IgD on IgD+ CD38- B cell.funnel_plot.pdf]

# MR Test

- Inverse variance weighted
- MR Egger
- Simple mode
- Weighted median
- Weighted mode

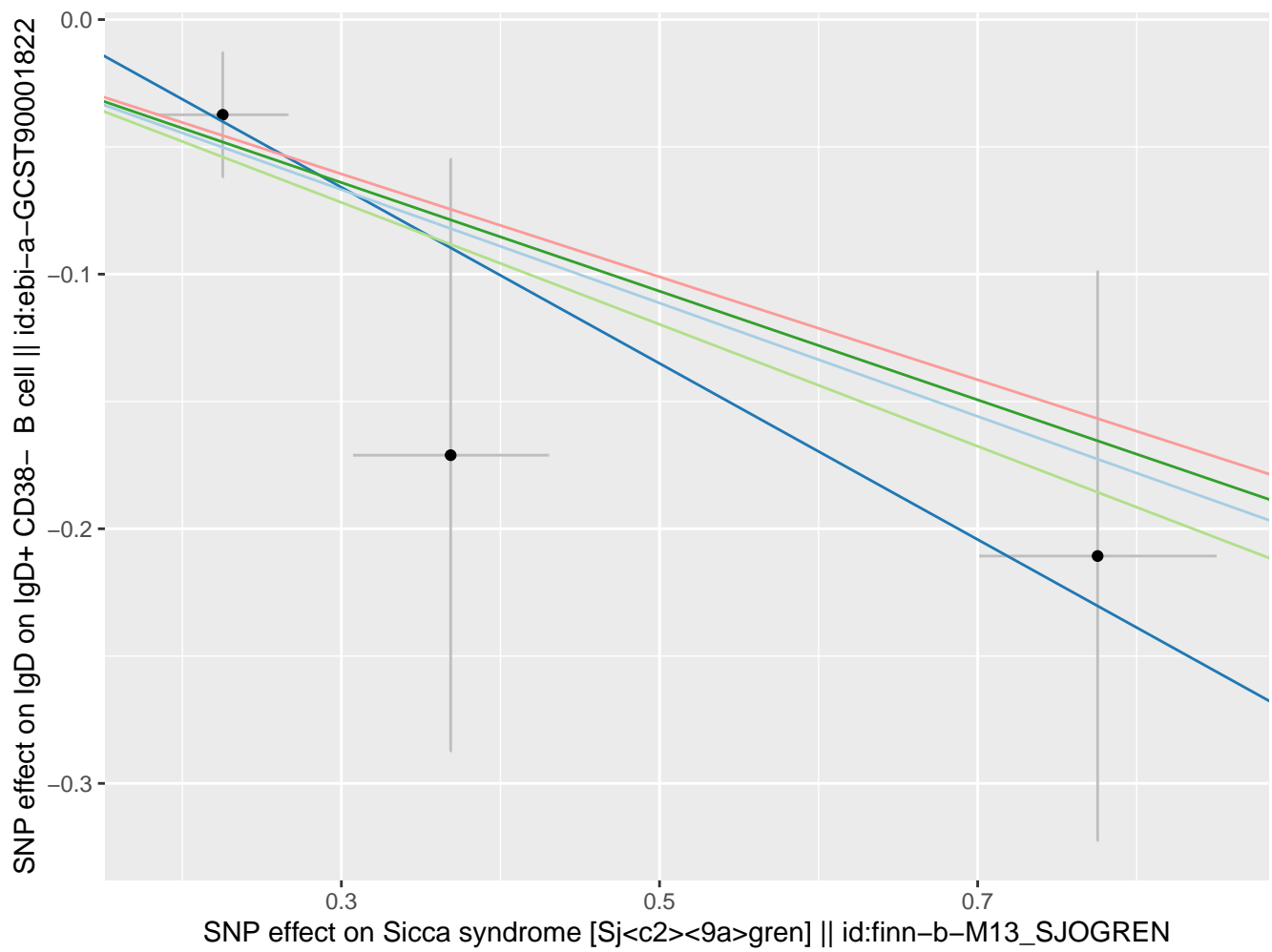

Supplement: Supplementary file 1 [file Data_Sheet_1.ZIP › IgD on IgD+ CD38- B cell.scatter_plot.pdf]

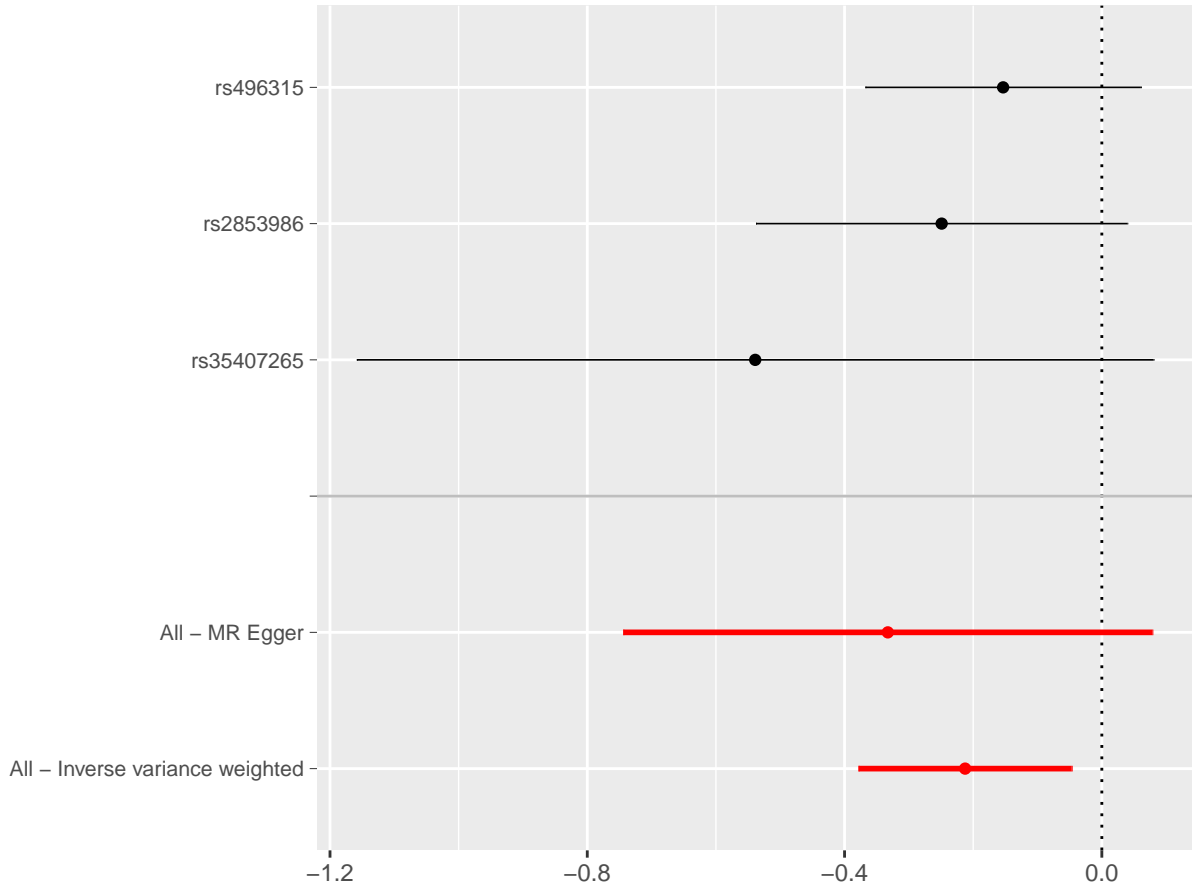

MR effect size for  
'Sicca syndrome [Sj<c2><9a>gren] || id:finn-b-M13\_SJOGREN' on 'IgD on IgD+ CD38dim B cell || id:ebi-a-G

Supplement: Supplementary file 1 [file Data_Sheet_1.ZIP › IgD on IgD+ CD38dim B cell.forest.pdf]

# MR Method

- Inverse variance weighted
- MR Egger

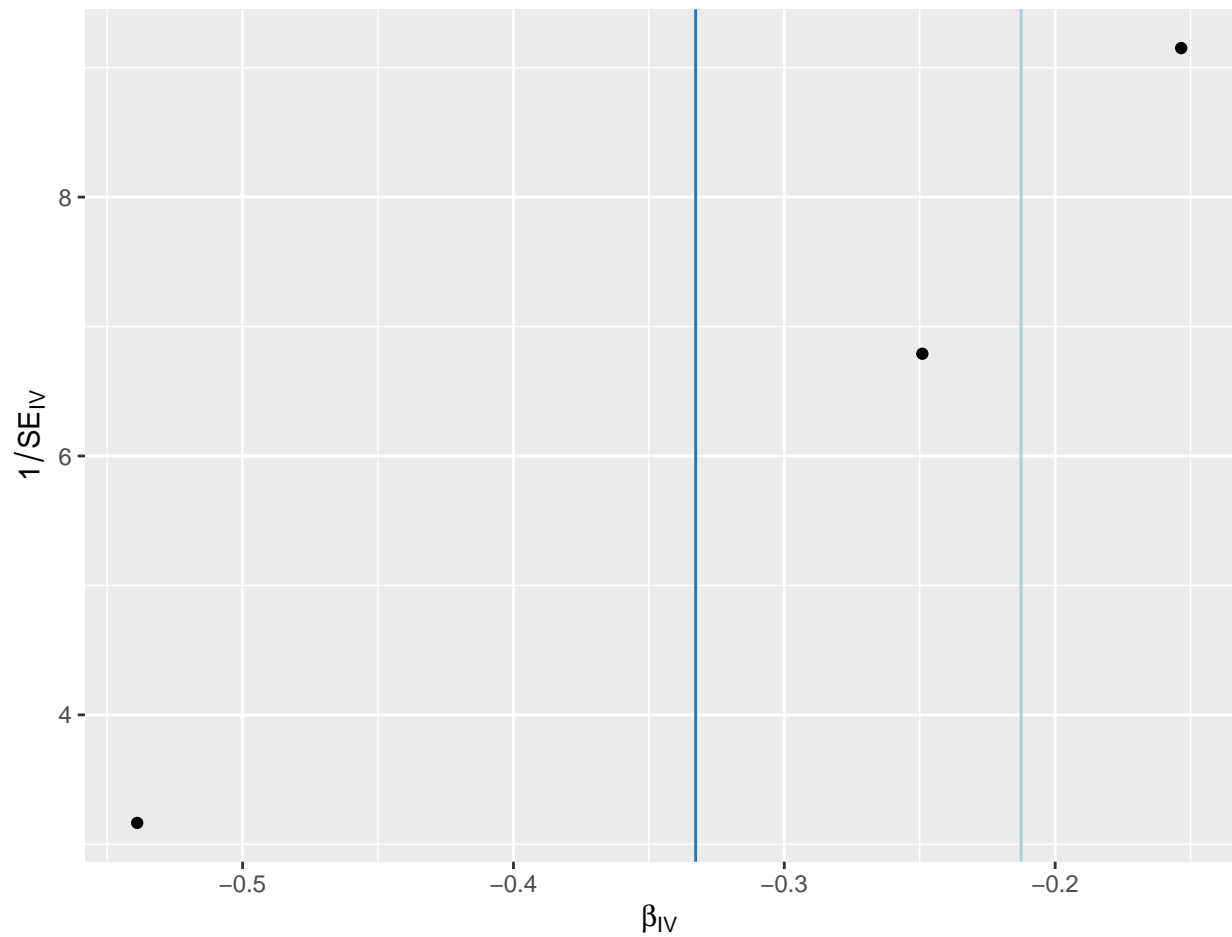

Supplement: Supplementary file 1 [file Data_Sheet_1.ZIP › IgD on IgD+ CD38dim B cell.funnel_plot.pdf]

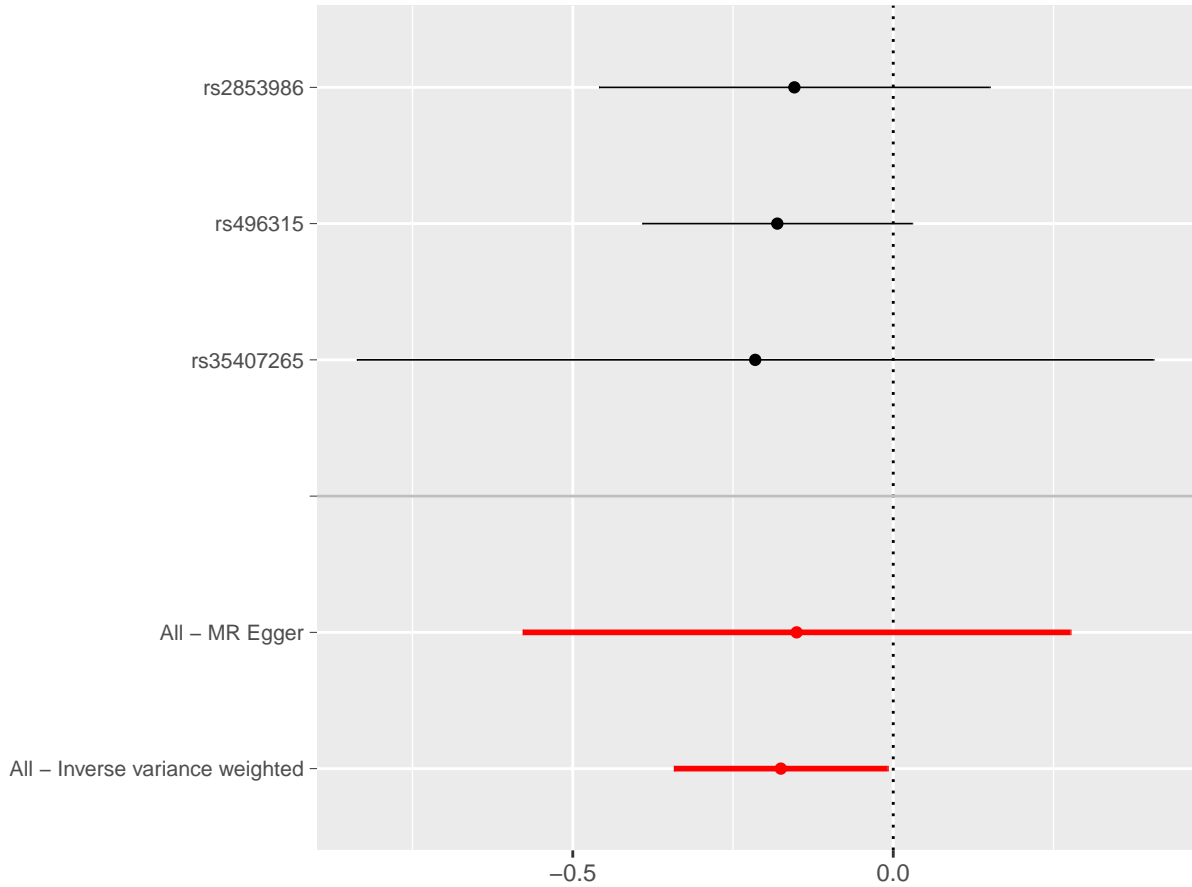

MR effect size for  
'Sicca syndrome [Sj<c2><9a>gren] || id:finn-b-M13\_SJOGREN' on 'lgD- CD27- B cell %lymphocyte || id:ebi-a-

Supplement: Supplementary file 1 [file Data_Sheet_1.ZIP › IgD- CD27- B cell %lymphocyte.forest.pdf]

# MR Method

- Inverse variance weighted
- MR Egger

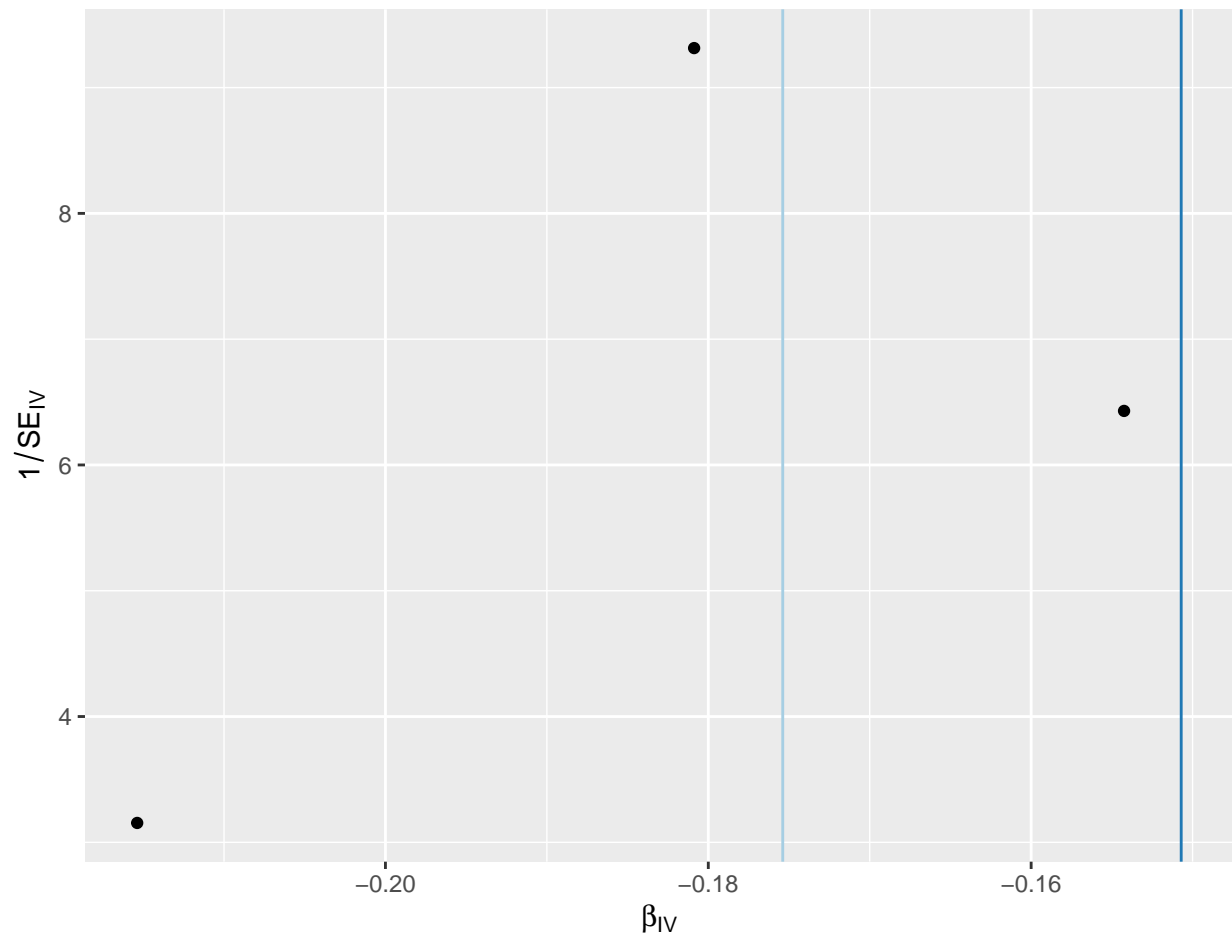

Supplement: Supplementary file 1 [file Data_Sheet_1.ZIP › IgD- CD27- B cell %lymphocyte.funnel_plot.pdf]

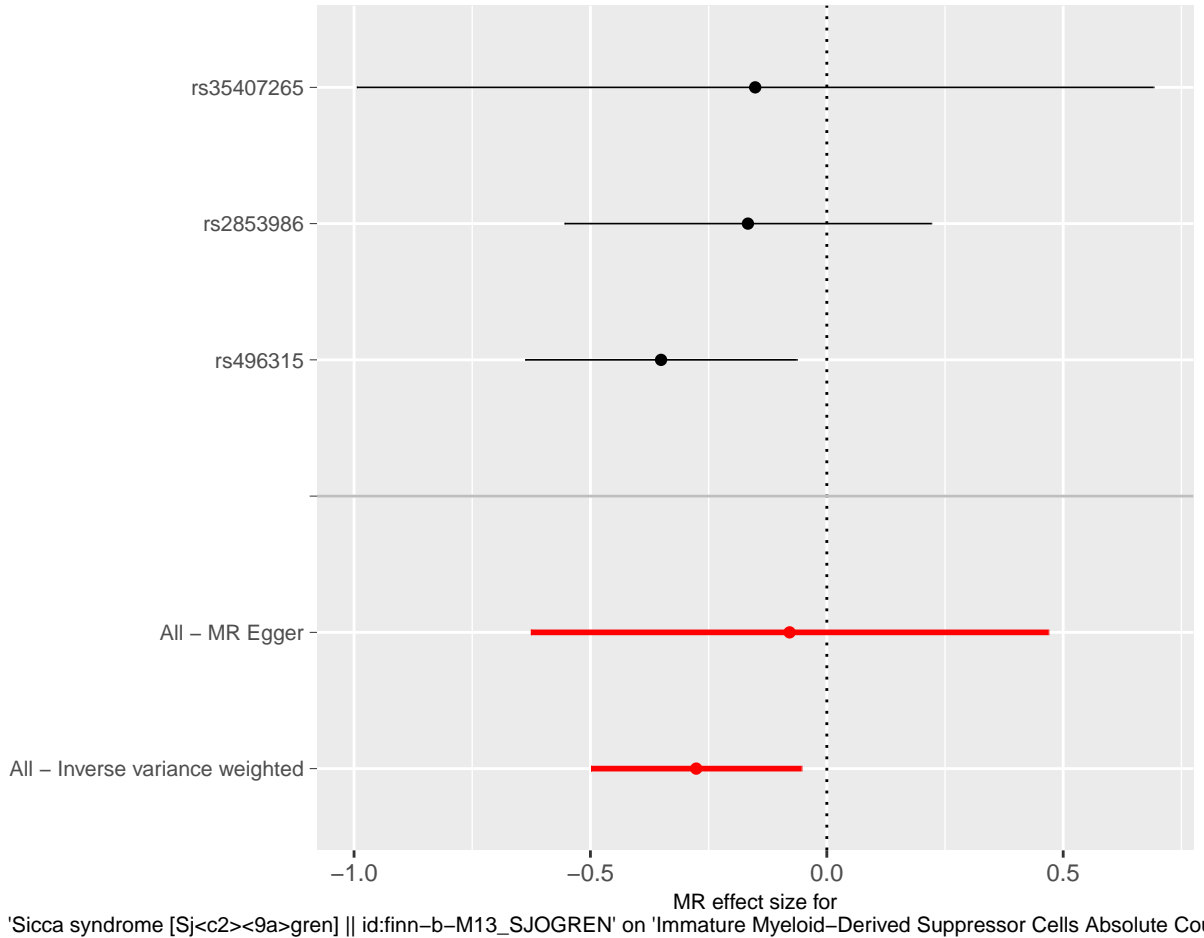

Supplement: Supplementary file 1 [file Data_Sheet_1.ZIP › Immature Myeloid-Derived Suppressor Cells Absolute Count.forest.pdf]

# MR Method

- Inverse variance weighted
- MR Egger

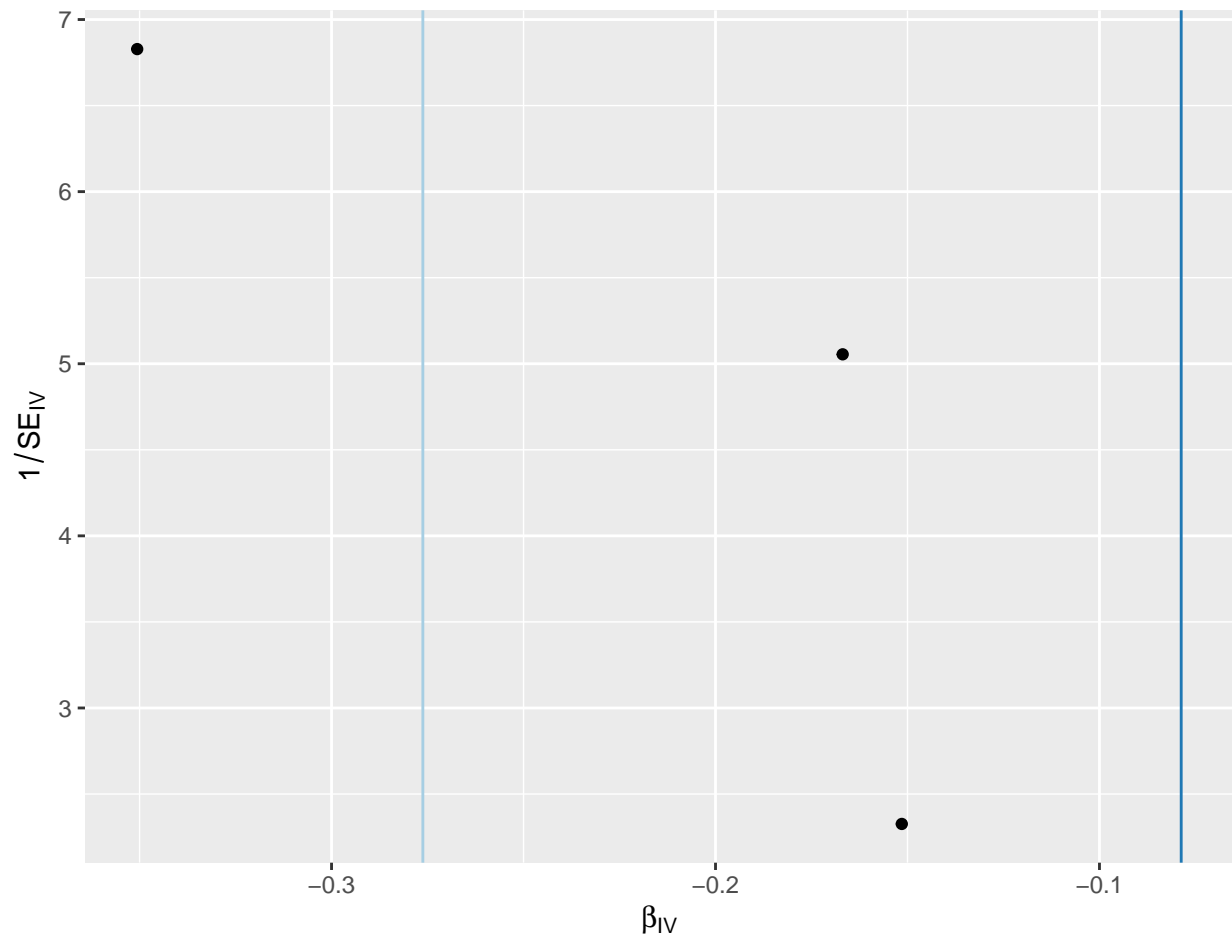

Supplement: Supplementary file 1 [file Data_Sheet_1.ZIP › Immature Myeloid-Derived Suppressor Cells Absolute Count.funnel_plot.pdf]

# MR Test

- Inverse variance weighted
- MR Egger
- Simple mode
- Weighted median
- Weighted mode

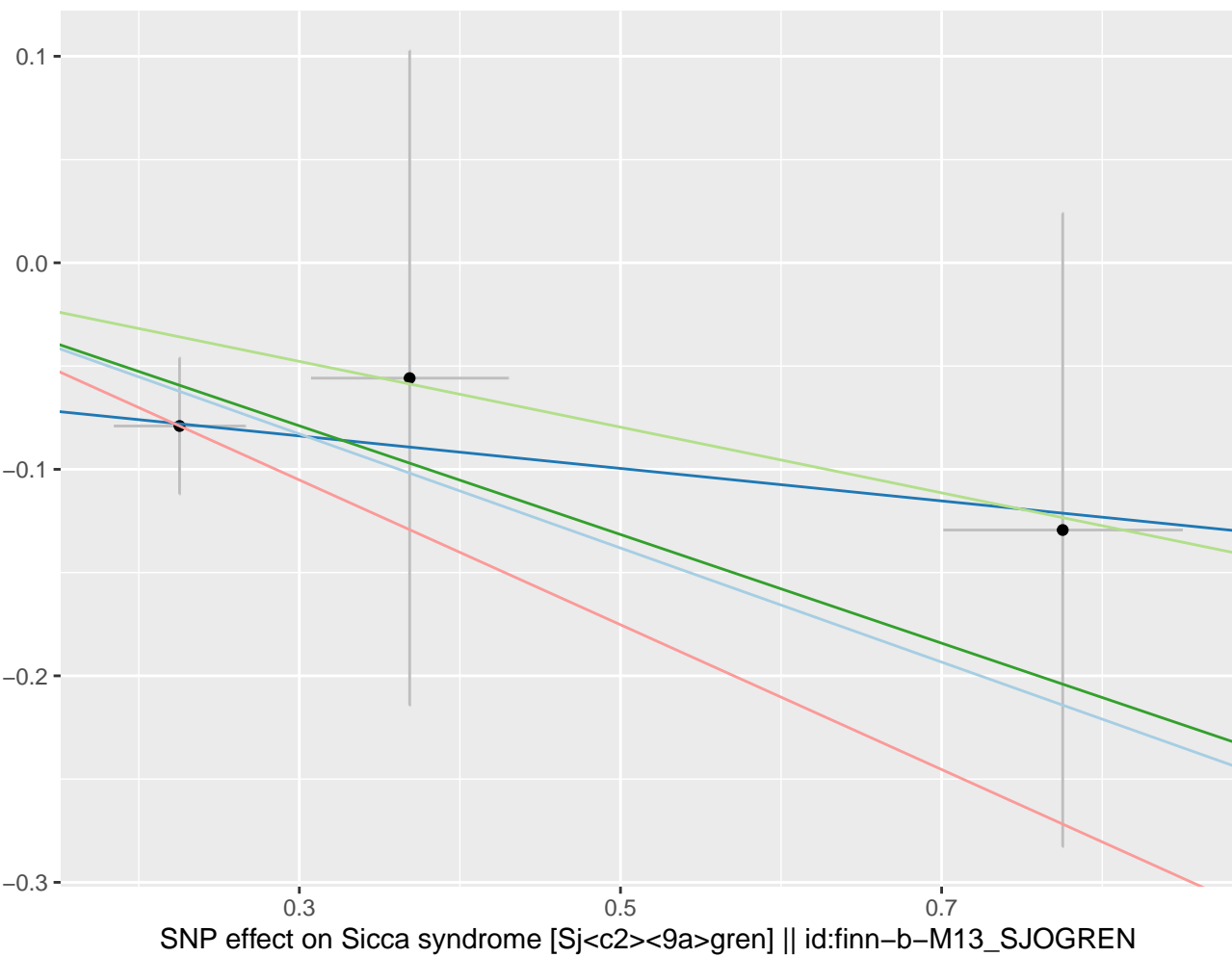

Supplement: Supplementary file 1 [file Data_Sheet_1.ZIP › Immature Myeloid-Derived Suppressor Cells Absolute Count.scatter_plot.pdf]

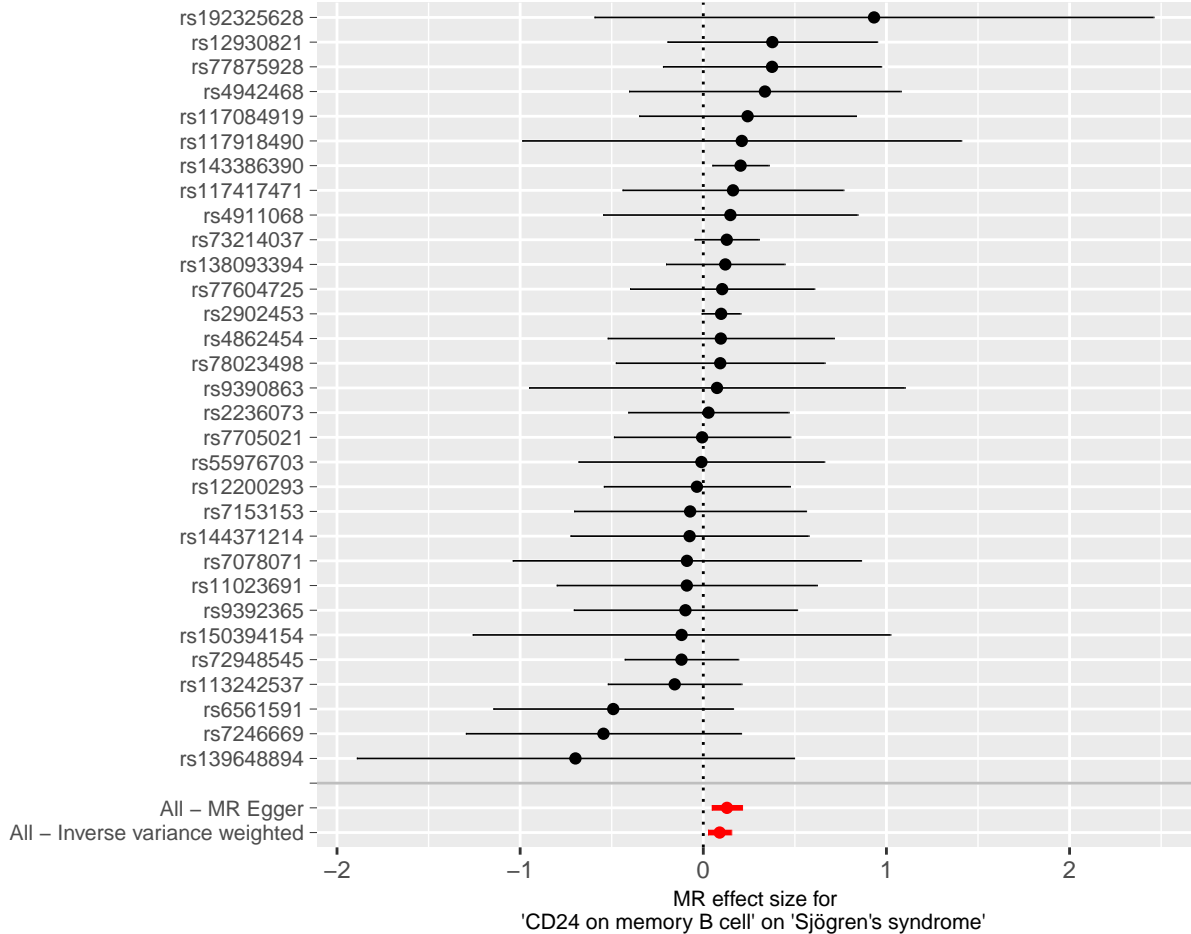

Supplement: Supplementary file 2 [file Data_Sheet_2.ZIP › CD24 on memory B cell.forest.pdf]

# MR Method

- Inverse variance weighted
- MR Egger

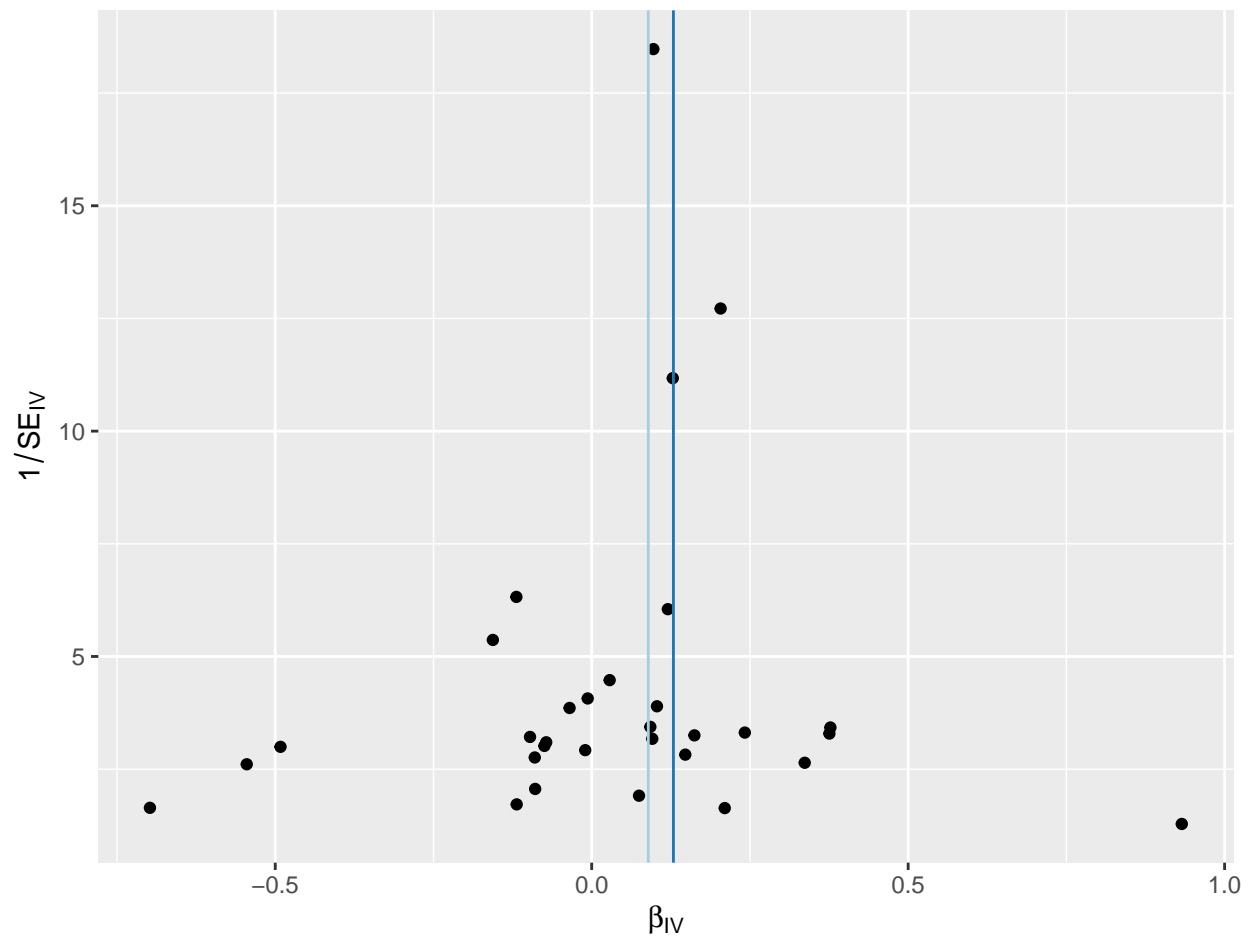

Supplement: Supplementary file 2 [file Data_Sheet_2.ZIP › CD24 on memory B cell.funnel_plot.pdf]

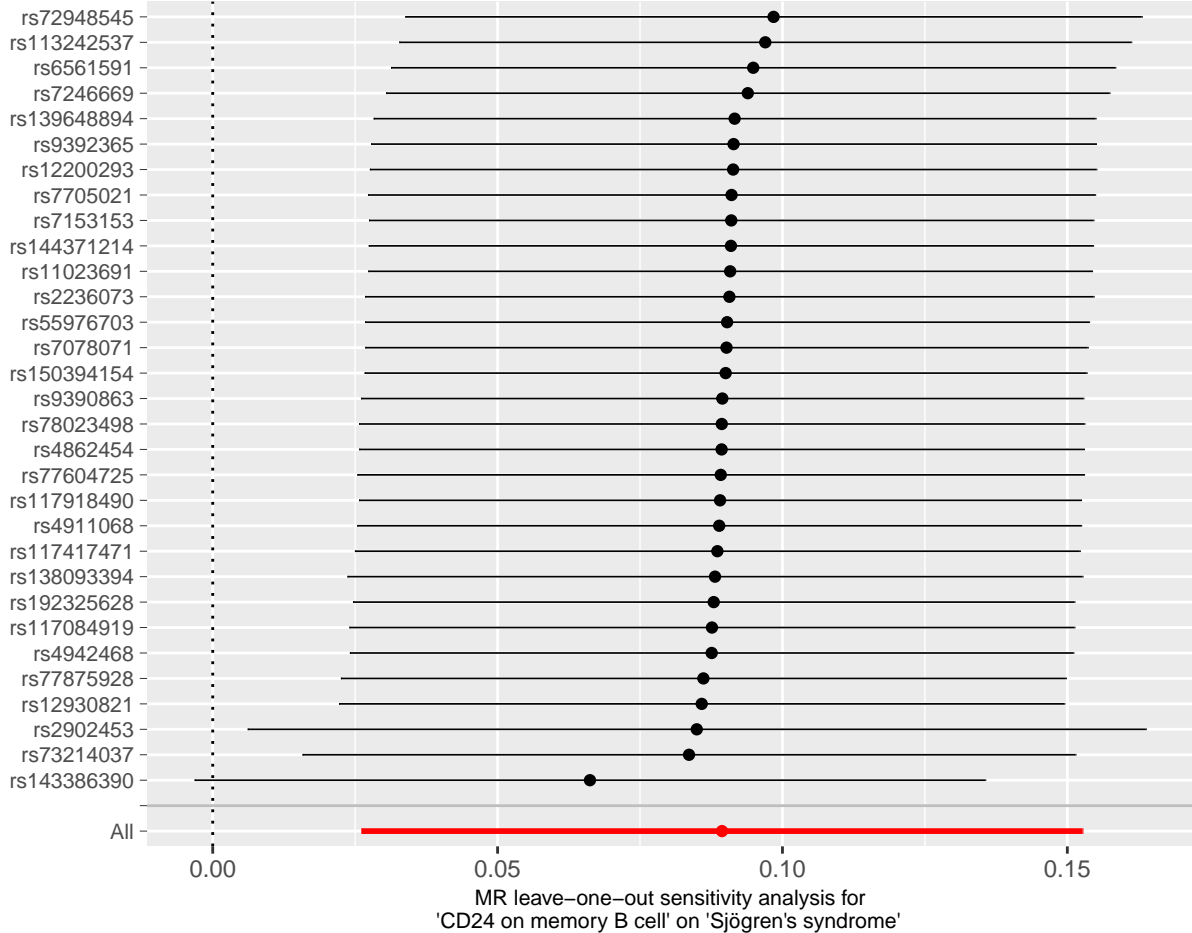

Supplement: Supplementary file 2 [file Data_Sheet_2.ZIP › CD24 on memory B cell.leaveoneout.pdf]

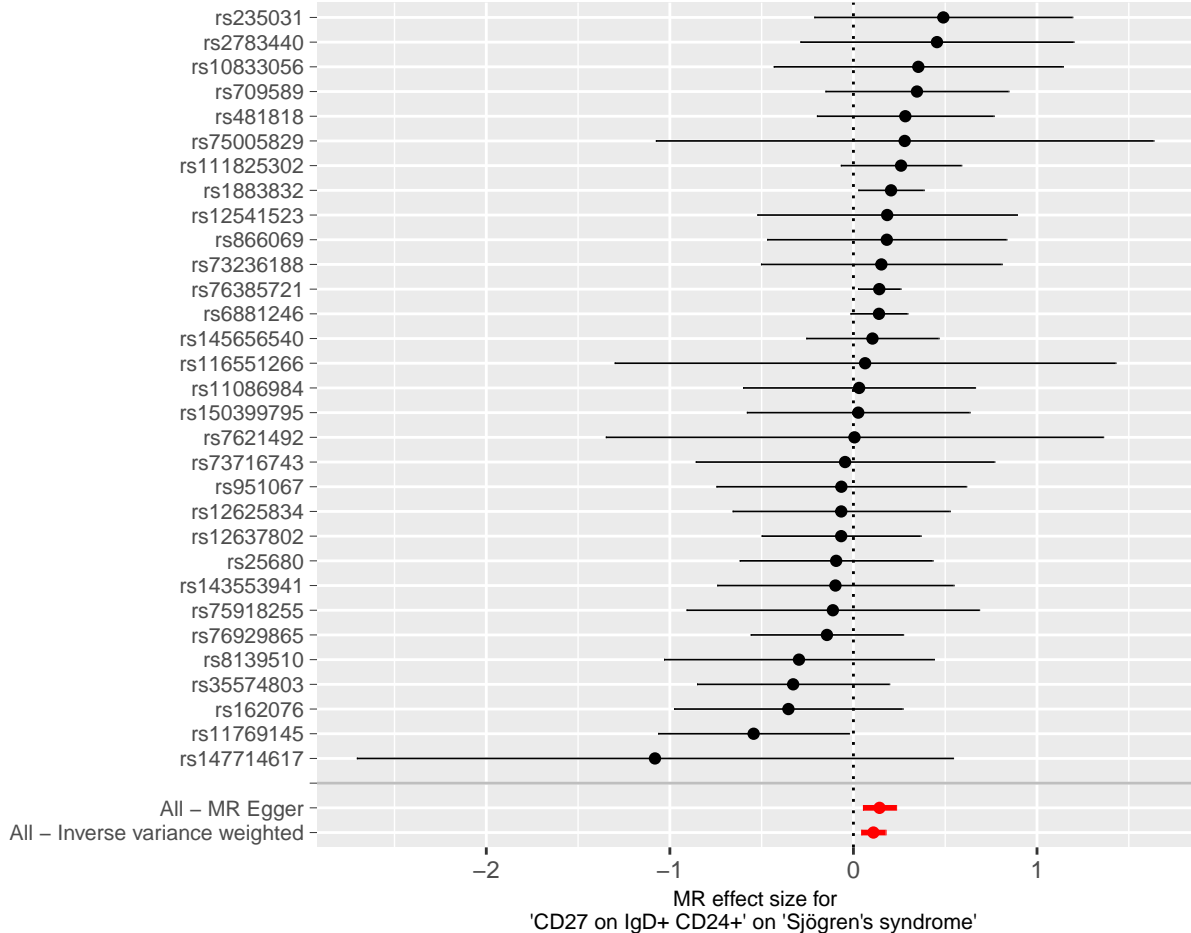

Supplement: Supplementary file 2 [file Data_Sheet_2.ZIP › CD27 on IgD+ CD24+ B cell.forest.pdf]

# MR Method

- Inverse variance weighted
- MR Egger

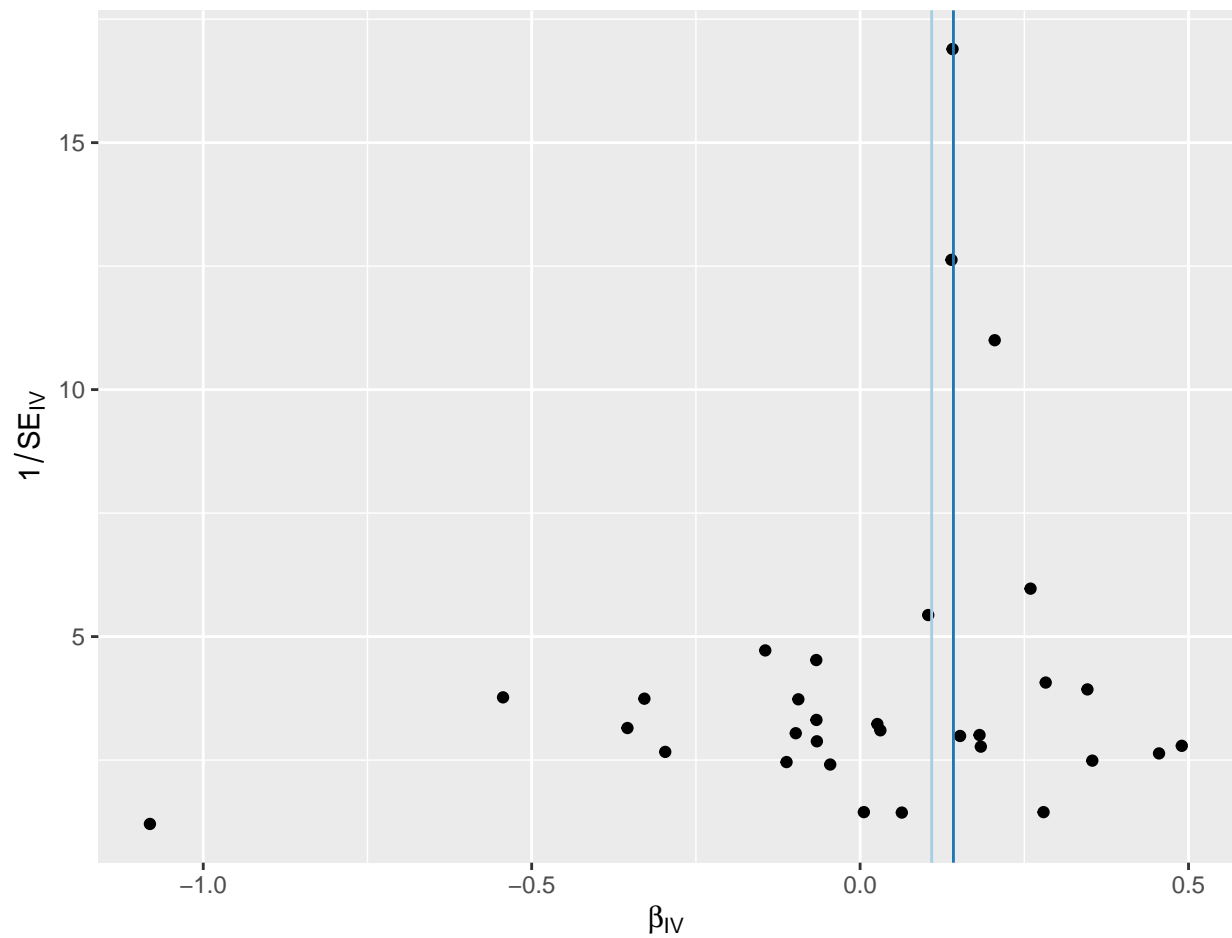

Supplement: Supplementary file 2 [file Data_Sheet_2.ZIP › CD27 on IgD+ CD24+ B cell.funnel_plot.pdf]

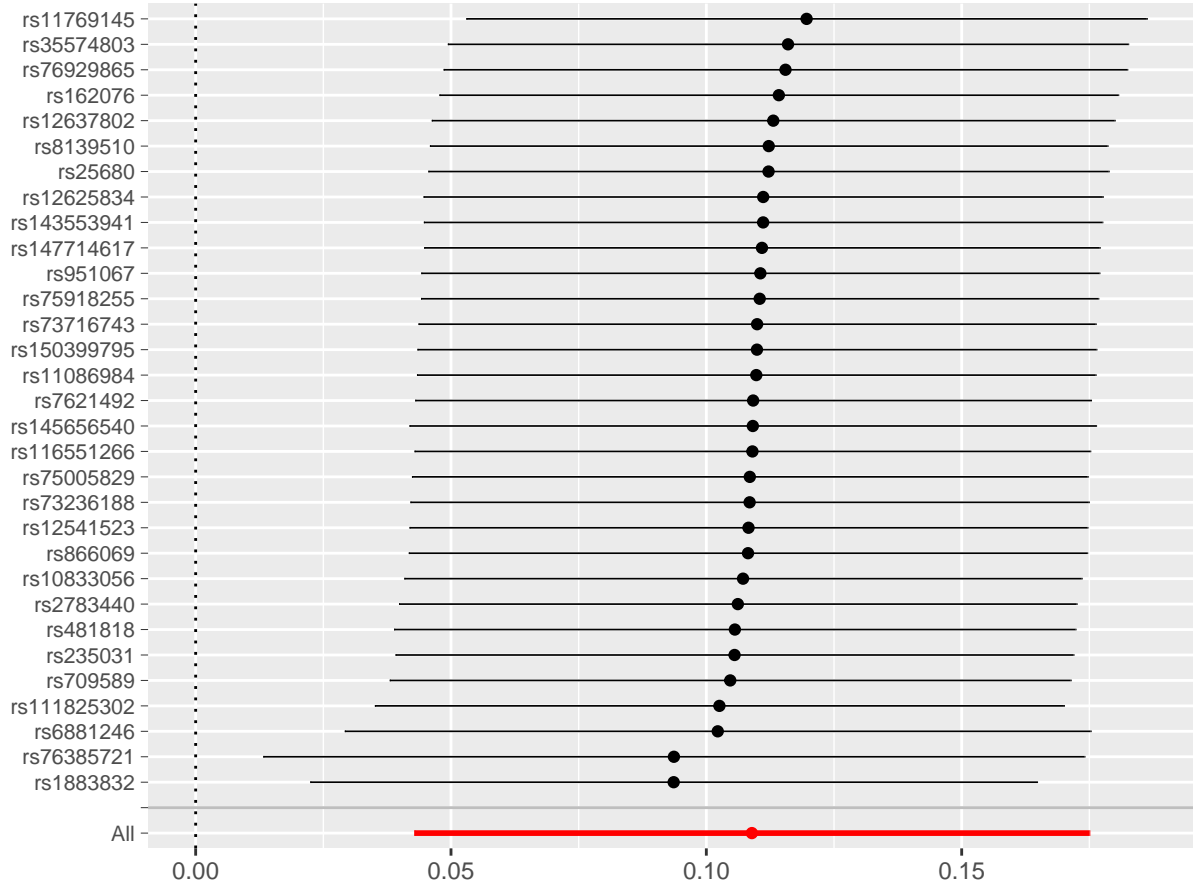

MR leave-one-out sensitivity analysis for  
'CD27 on IgD+ CD24+' on 'Sjögren's syndrome'

Supplement: Supplementary file 2 [file Data_Sheet_2.ZIP › CD27 on IgD+ CD24+ B cell.leaveoneout.pdf]

# MR Test

- Inverse variance weighted
- MR Egger
- Simple mode
- Weighted median
- Weighted mode

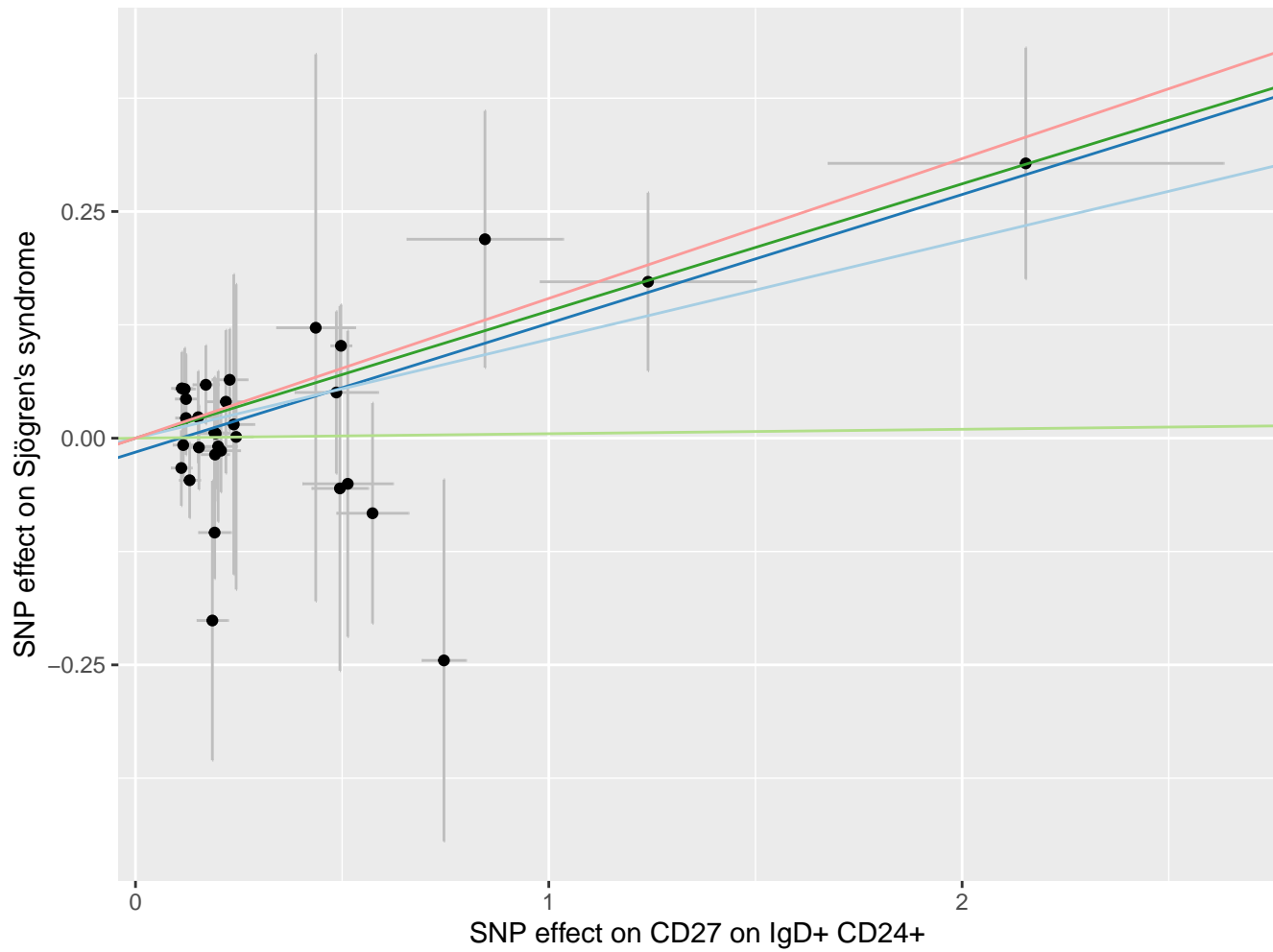

Supplement: Supplementary file 2 [file Data_Sheet_2.ZIP › CD27 on IgD+ CD24+ B cell.scatter_plot.pdf]

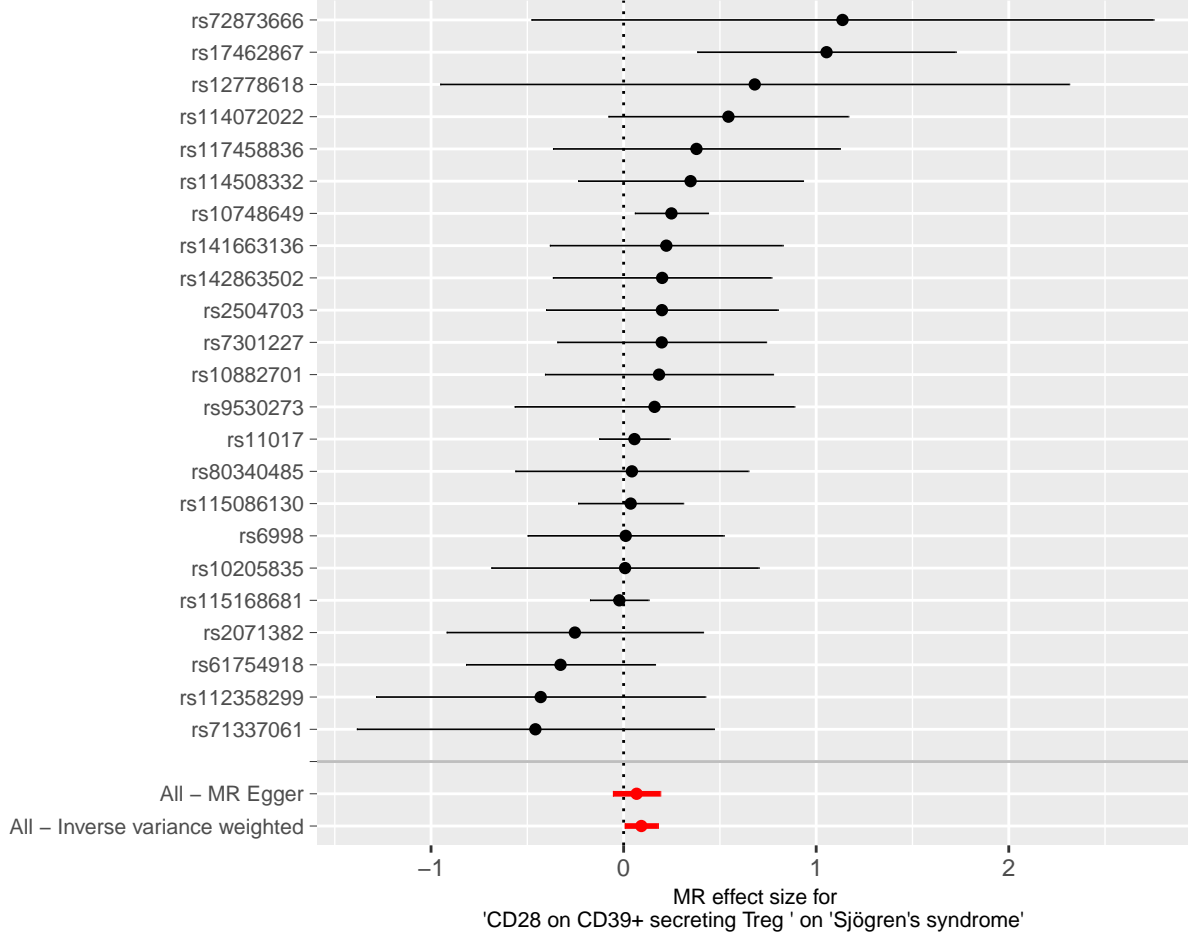

Supplement: Supplementary file 2 [file Data_Sheet_2.ZIP › CD28 on CD39+ secreting CD4 regulatory T cell.forest.pdf]

# MR Method

- Inverse variance weighted
- MR Egger

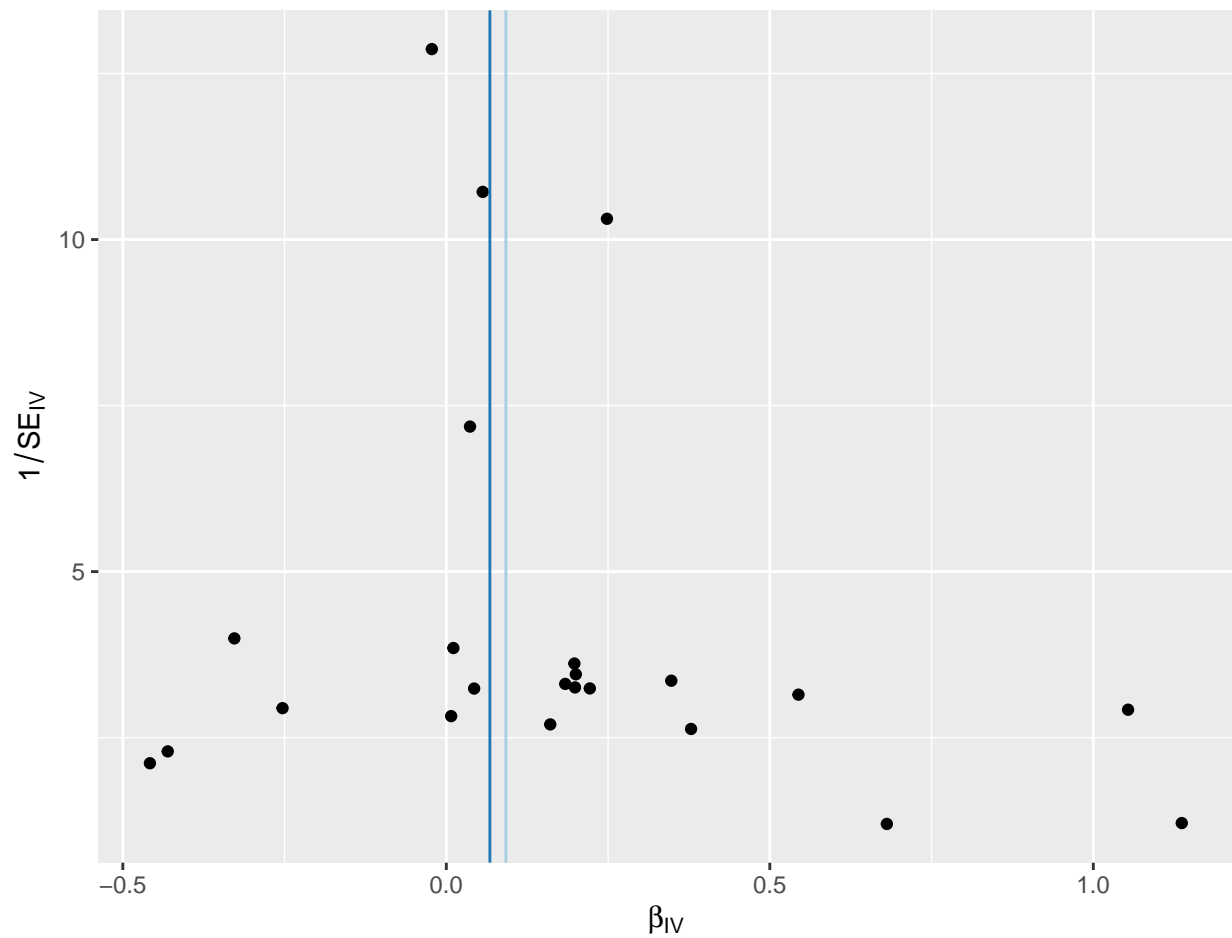

Supplement: Supplementary file 2 [file Data_Sheet_2.ZIP › CD28 on CD39+ secreting CD4 regulatory T cell.funnel_plot.pdf]

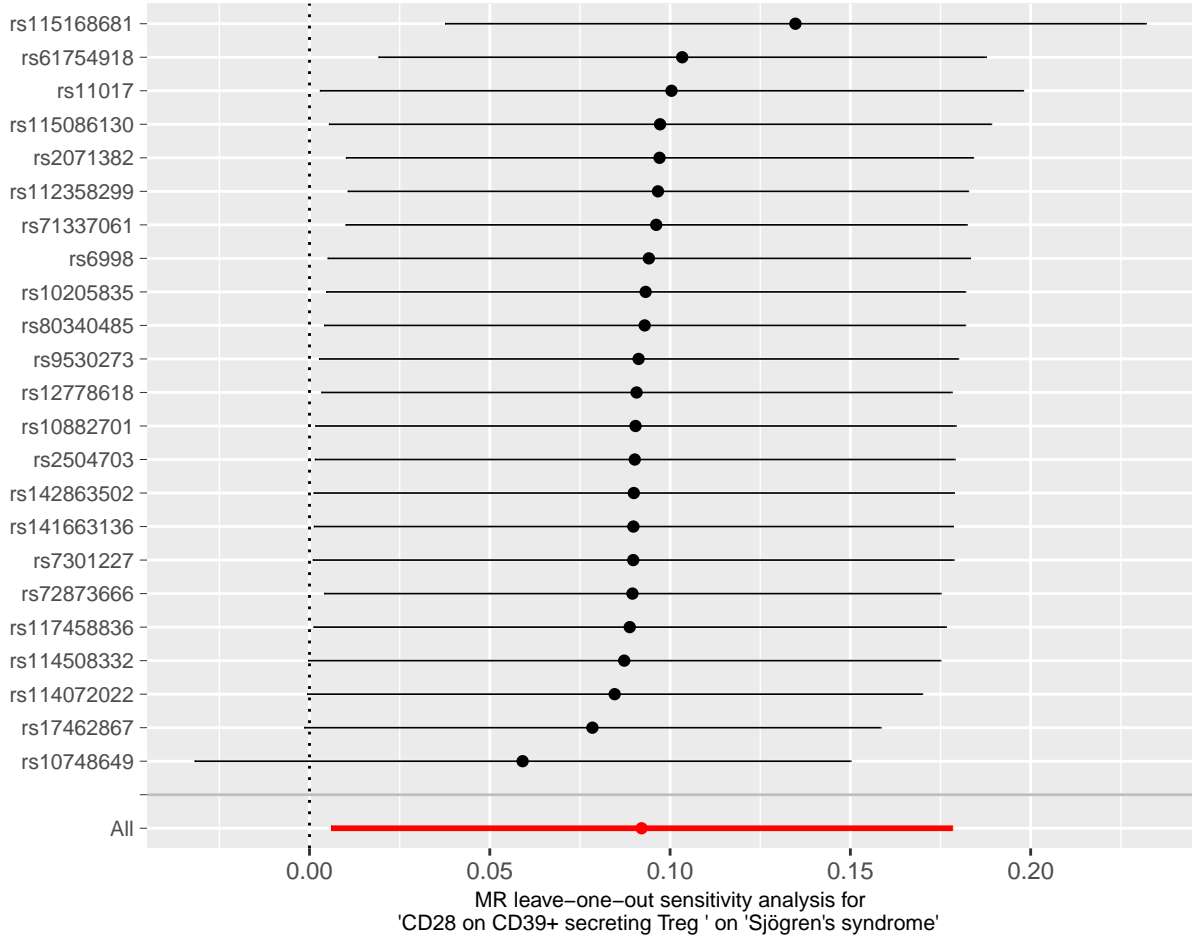

Supplement: Supplementary file 2 [file Data_Sheet_2.ZIP › CD28 on CD39+ secreting CD4 regulatory T cell.leaveoneout.pdf]

# MR Test

- Inverse variance weighted
- MR Egger
- Simple mode
- Weighted median
- Weighted mode

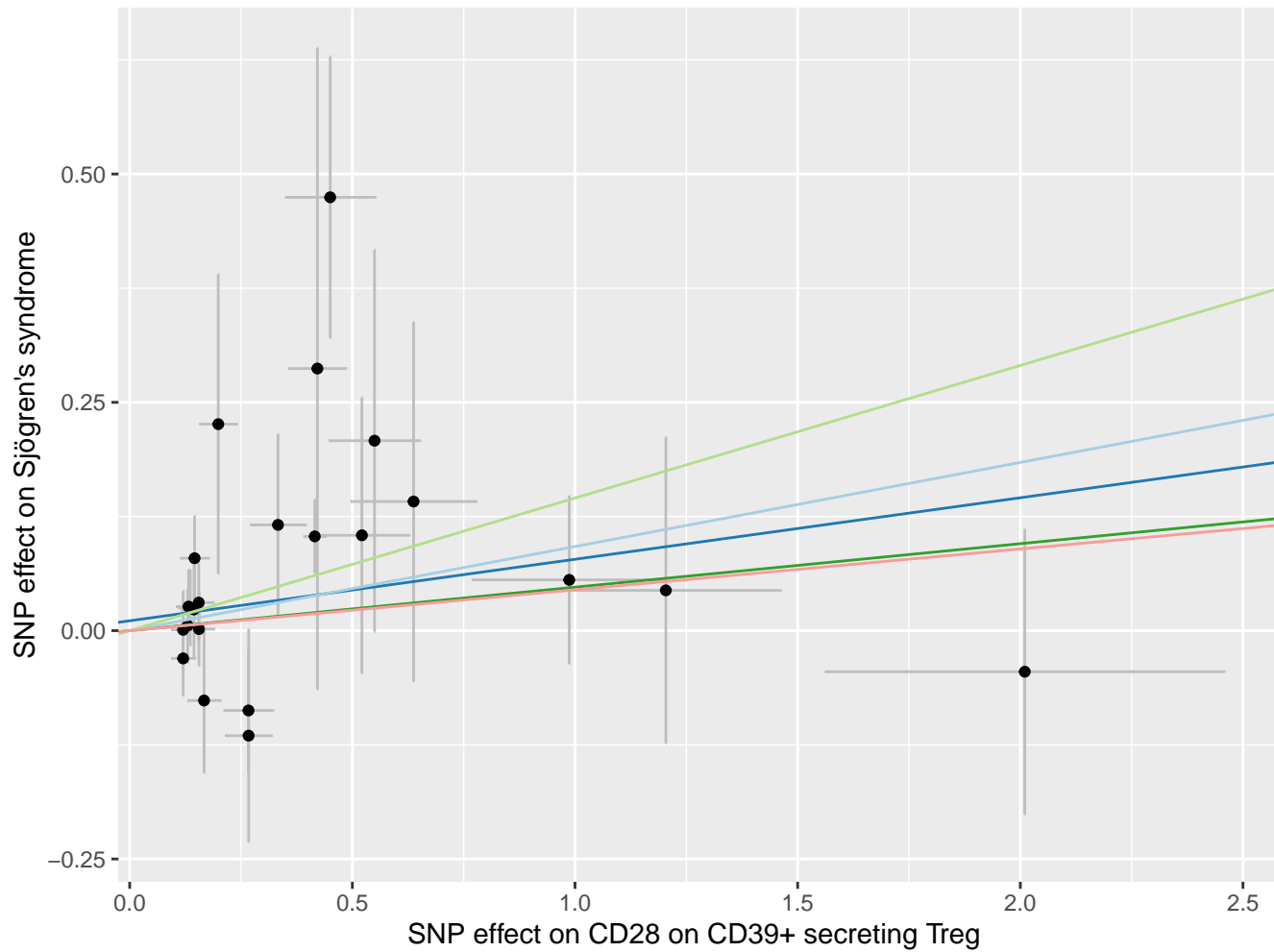

Supplement: Supplementary file 2 [file Data_Sheet_2.ZIP › CD28 on CD39+ secreting CD4 regulatory T cell.scatter_plot.pdf]

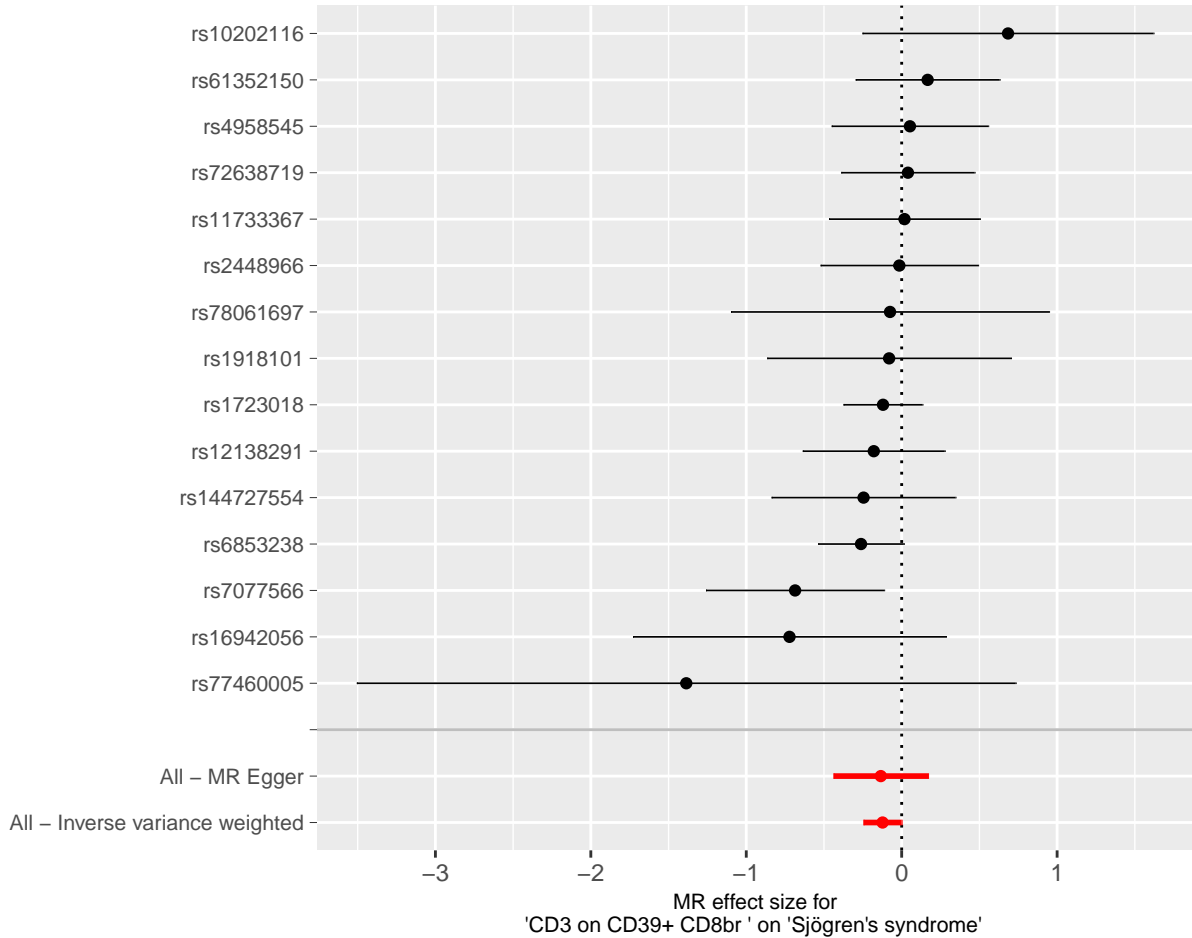

Supplement: Supplementary file 2 [file Data_Sheet_2.ZIP › CD3 on CD39+ CD8+ T cell.forest.pdf]

# MR Method

- Inverse variance weighted
- MR Egger

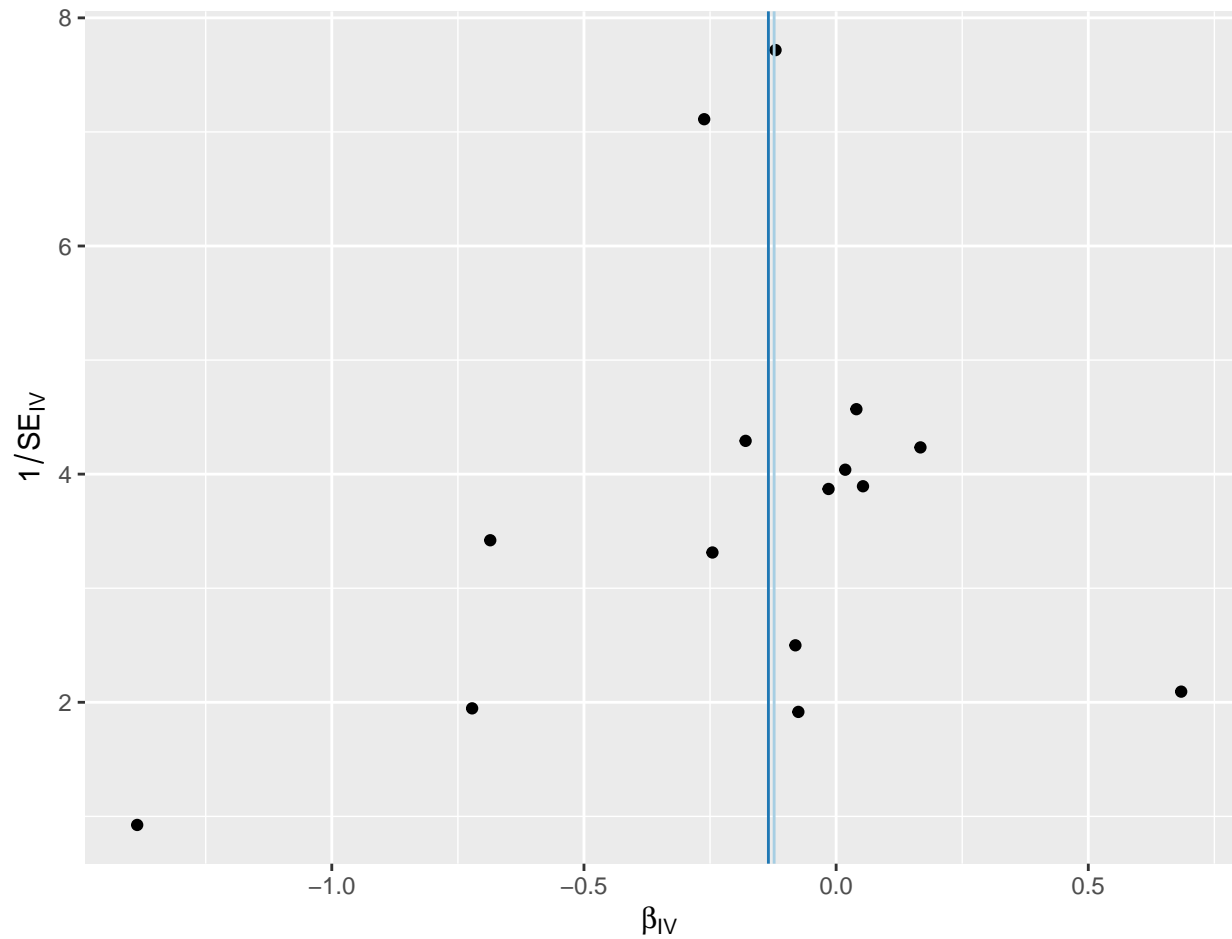

Supplement: Supplementary file 2 [file Data_Sheet_2.ZIP › CD3 on CD39+ CD8+ T cell.funnel_plot.pdf]

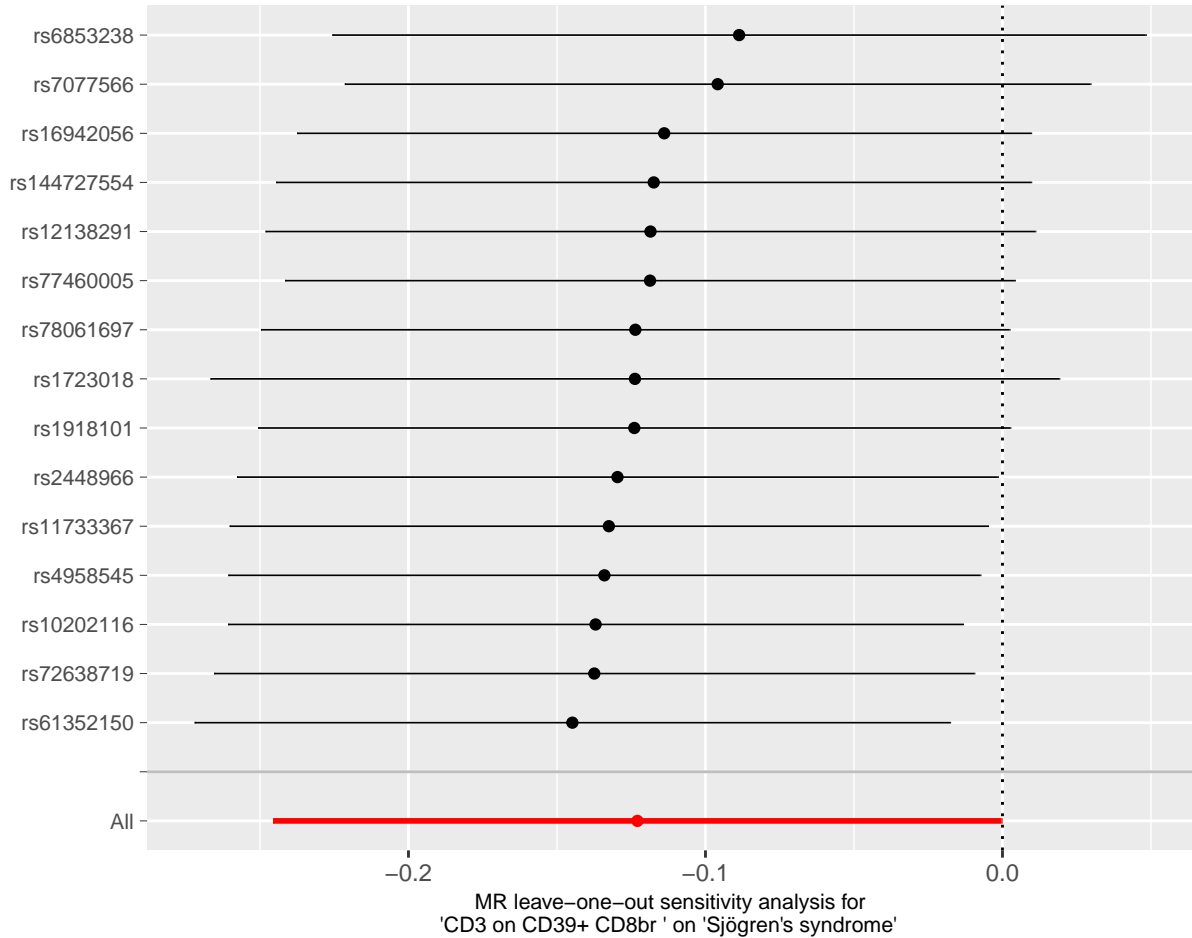

Supplement: Supplementary file 2 [file Data_Sheet_2.ZIP › CD3 on CD39+ CD8+ T cell.leaveoneout.pdf]

# MR Test

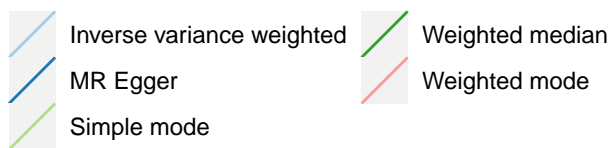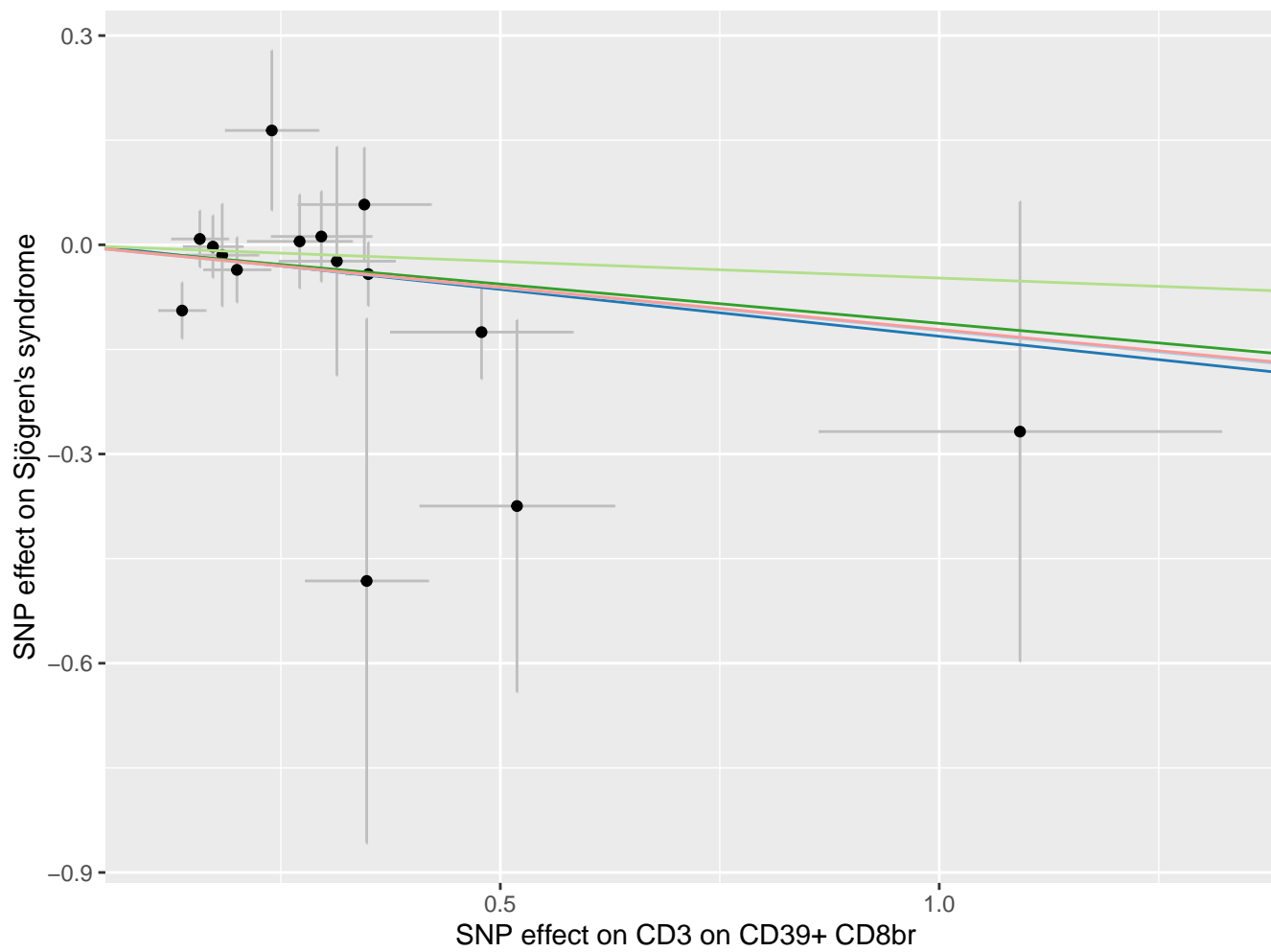

Supplement: Supplementary file 2 [file Data_Sheet_2.ZIP › CD3 on CD39+ CD8+ T cell.scatter_plot.pdf]

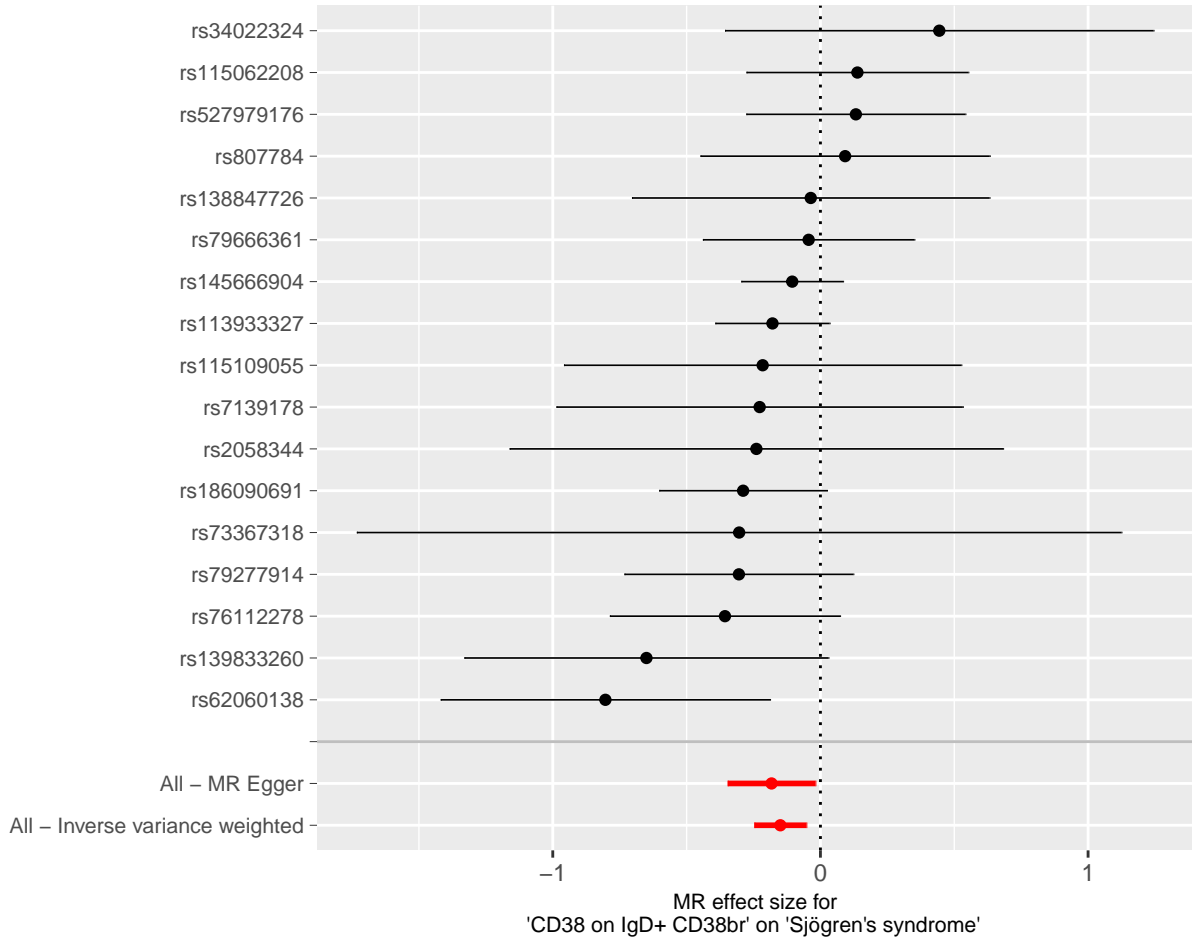

Supplement: Supplementary file 2 [file Data_Sheet_2.ZIP › CD38 on IgD+ CD38 B cell.forest.pdf]

# MR Method

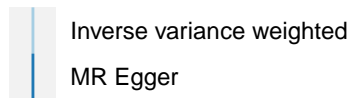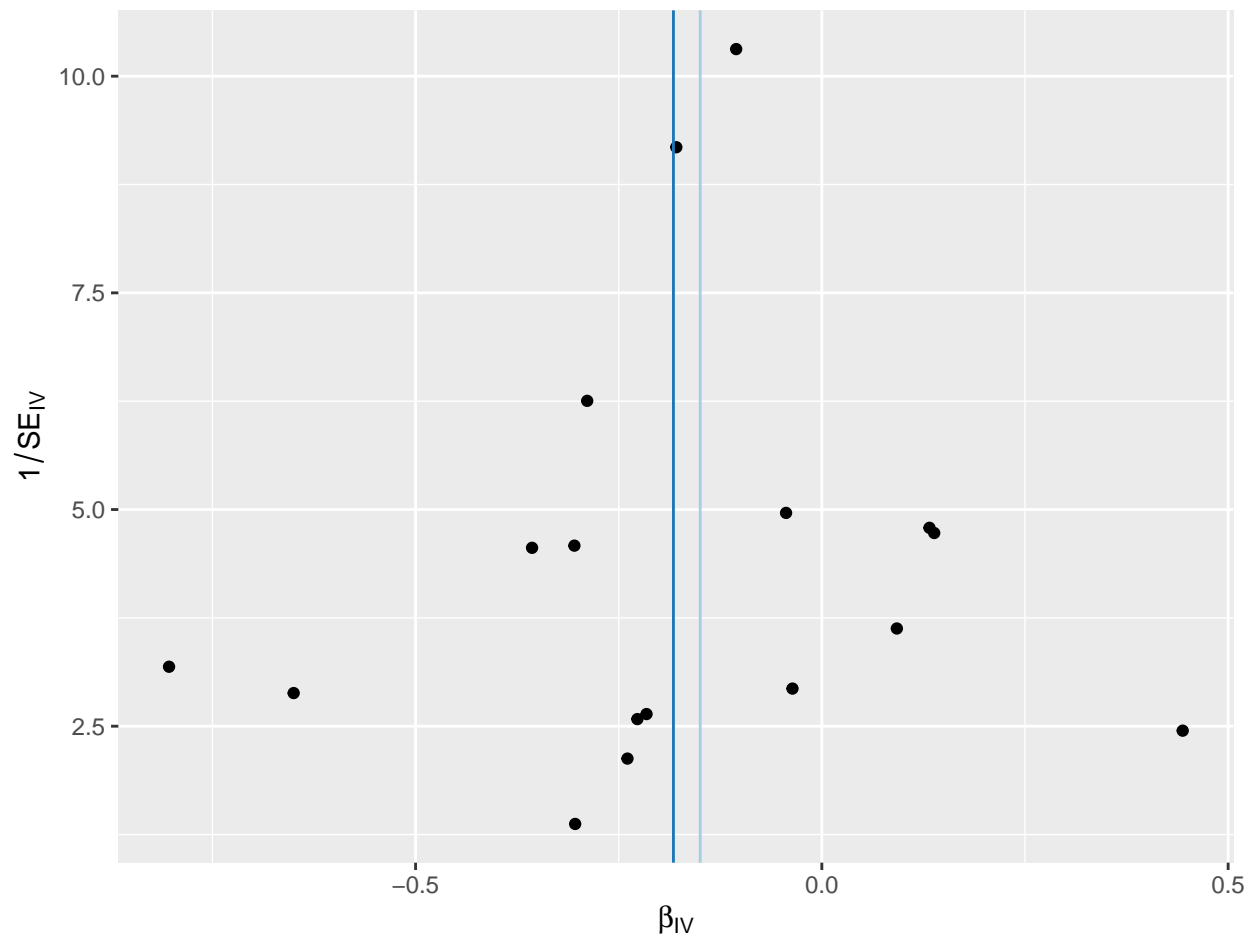

Supplement: Supplementary file 2 [file Data_Sheet_2.ZIP › CD38 on IgD+ CD38 B cell.funnel_plot.pdf]

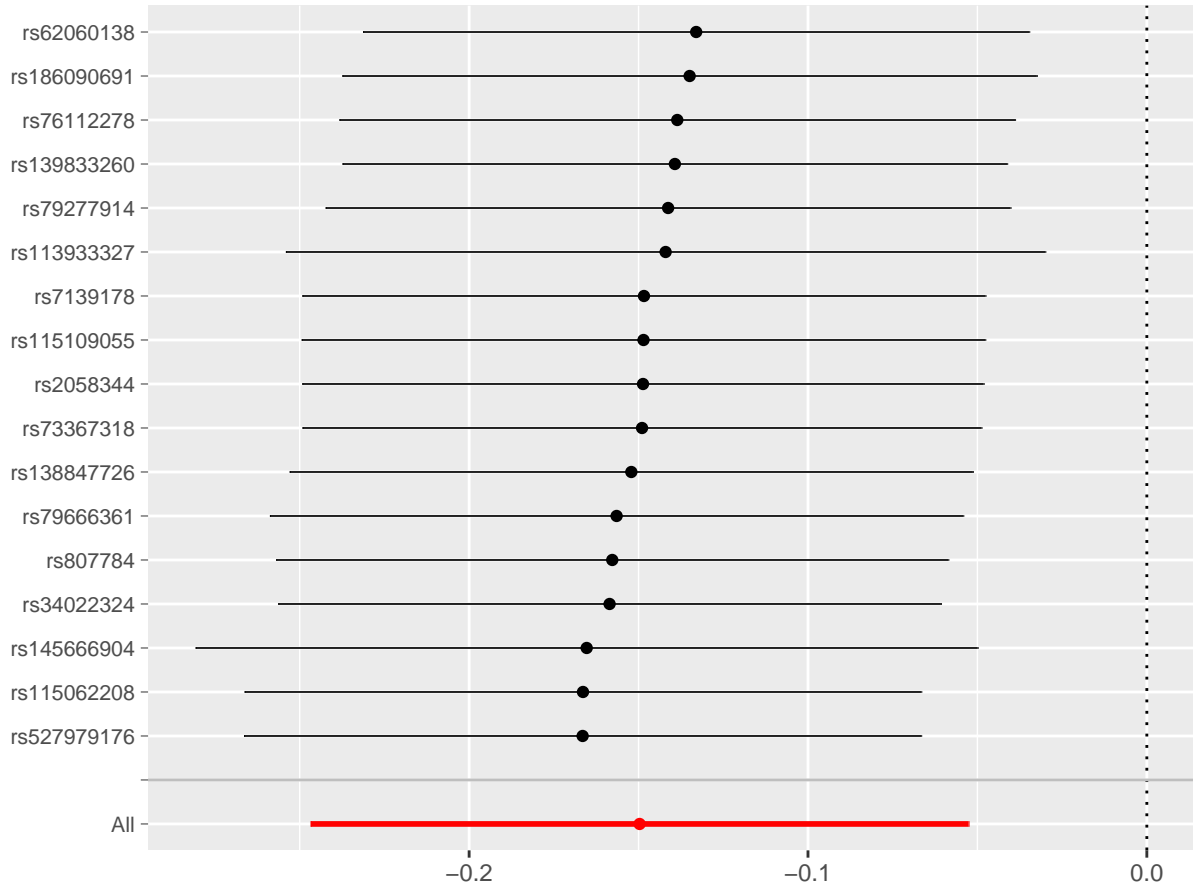

MR leave-one-out sensitivity analysis for  
'CD38 on IgD+ CD38br' on 'Sjögren's syndrome'

Supplement: Supplementary file 2 [file Data_Sheet_2.ZIP › CD38 on IgD+ CD38 B cell.leaveoneout.pdf]

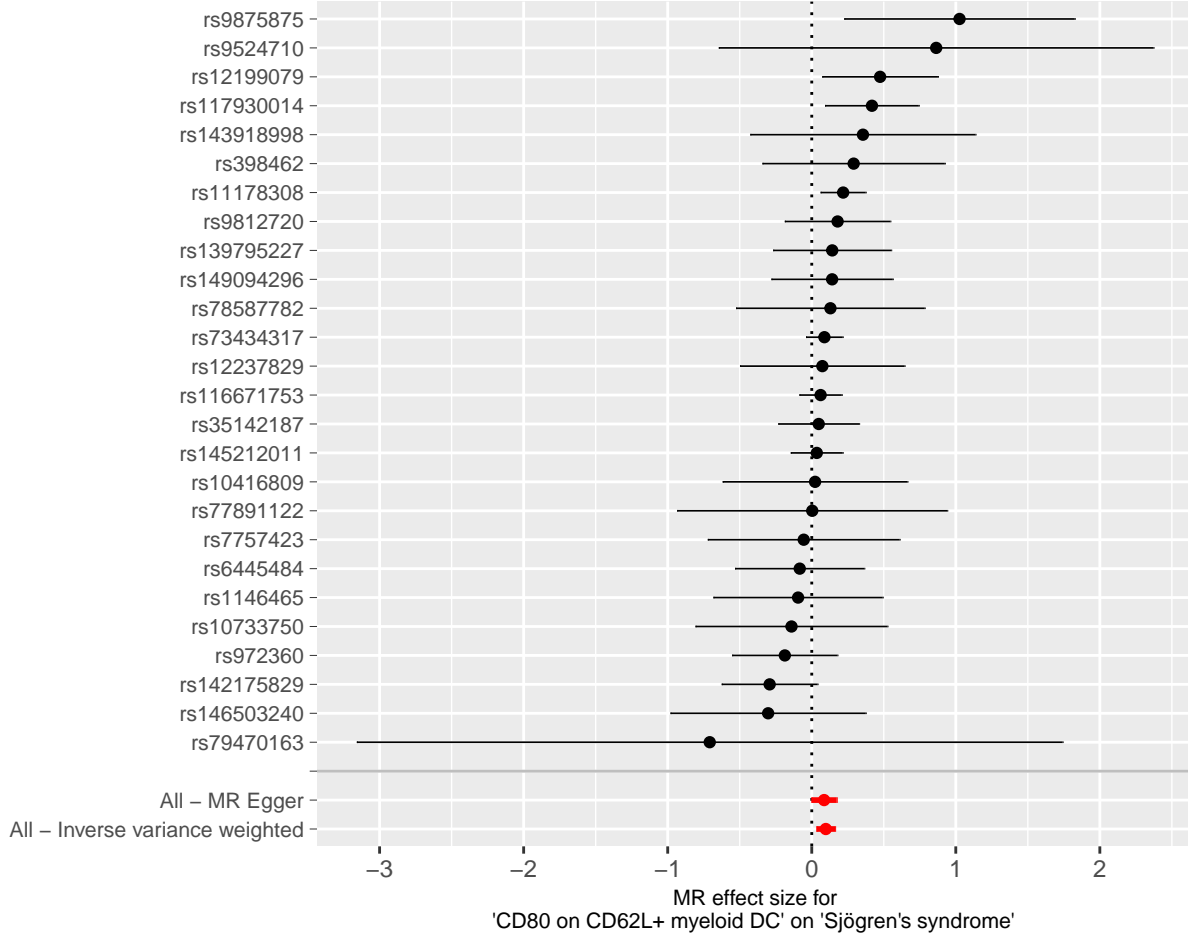

Supplement: Supplementary file 2 [file Data_Sheet_2.ZIP › CD80 on CD62L+ myeloid Dendritic Cell.forest.pdf]

# MR Method

- Inverse variance weighted
- MR Egger

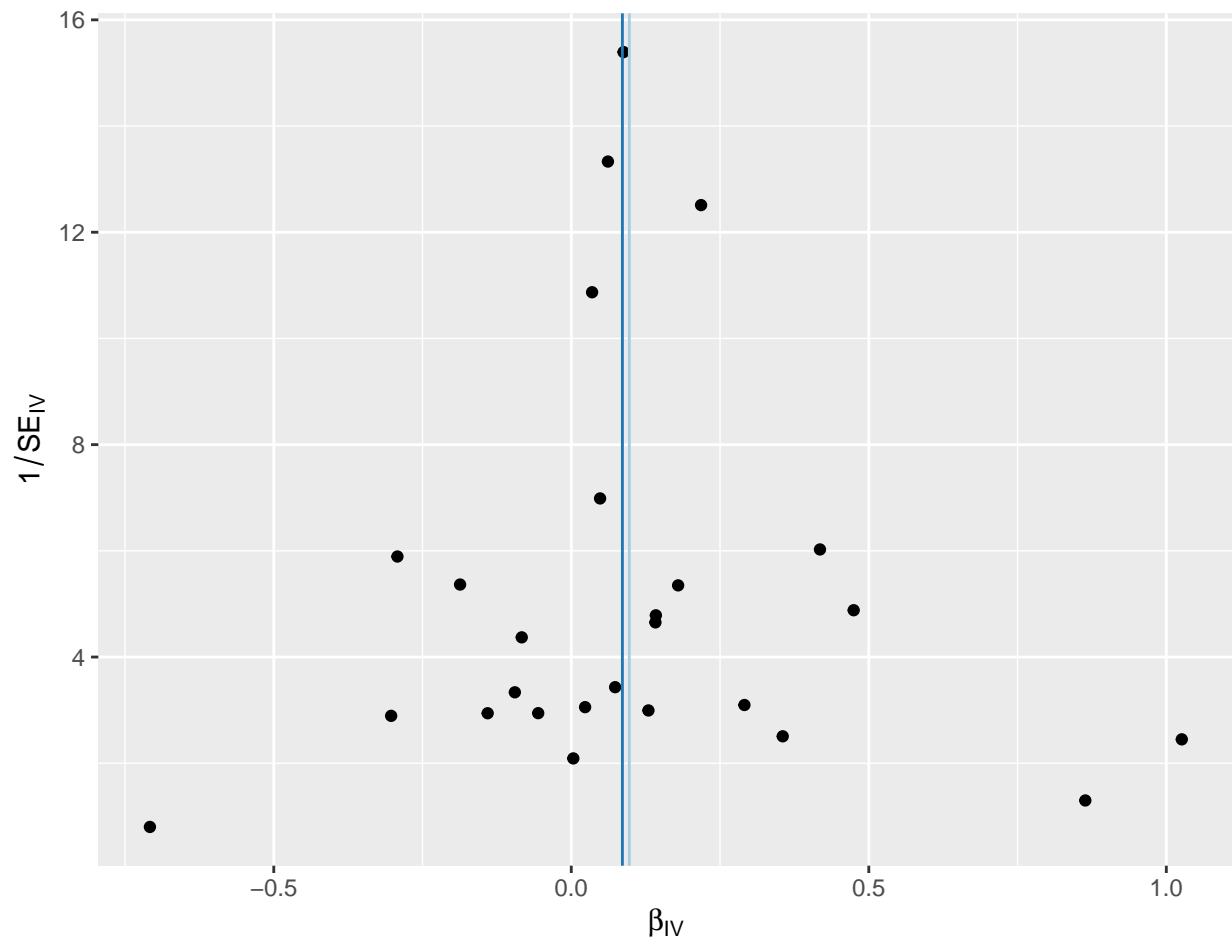

Supplement: Supplementary file 2 [file Data_Sheet_2.ZIP › CD80 on CD62L+ myeloid Dendritic Cell.funnel_plot.pdf]

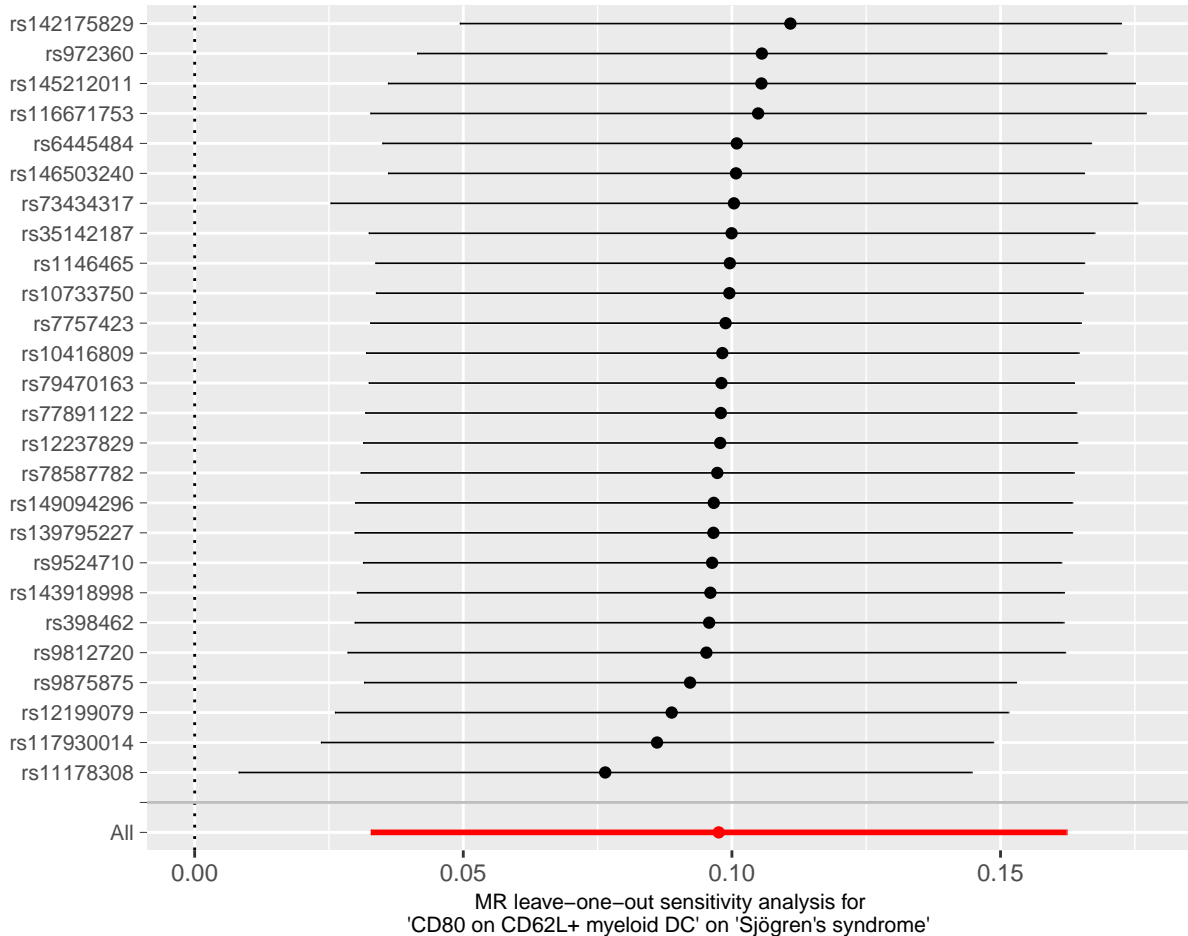

Supplement: Supplementary file 2 [file Data_Sheet_2.ZIP › CD80 on CD62L+ myeloid Dendritic Cell.leaveoneout.pdf]

# MR Test

- Inverse variance weighted
- MR Egger
- Simple mode
- Weighted median
- Weighted mode

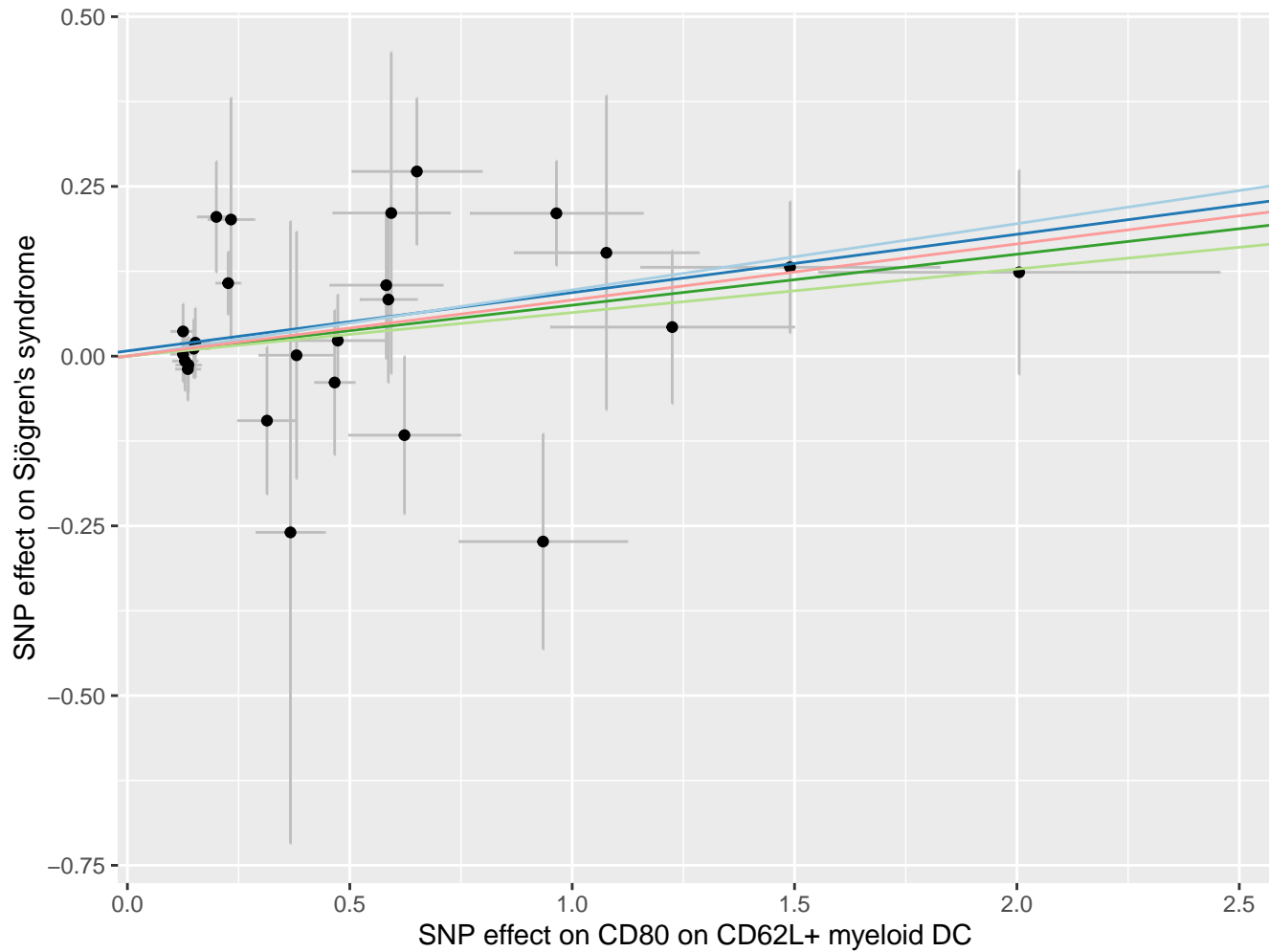

Supplement: Supplementary file 2 [file Data_Sheet_2.ZIP › CD80 on CD62L+ myeloid Dendritic Cell.scatter_plot.pdf]
